# Viscoelasticity, Like Forces, Plays a Role in Mechanotransduction

**DOI:** 10.3389/fcell.2022.789841

**Published:** 2022-02-09

**Authors:** Claudia Tanja Mierke

**Affiliations:** Faculty of Physics and Earth Science, Peter Debye Institute of Soft Matter Physics, Biological Physics Division, University of Leipzig, Leipzig, Germany

**Keywords:** cell mechanics, extracellular matrix constraints, organelles, viscoelasticity, cancer cells, stiffness, biophysical techniques, hallmarks of migration and invasion

## Abstract

Viscoelasticity and its alteration in time and space has turned out to act as a key element in fundamental biological processes in living systems, such as morphogenesis and motility. Based on experimental and theoretical findings it can be proposed that viscoelasticity of cells, spheroids and tissues seems to be a collective characteristic that demands macromolecular, intracellular component and intercellular interactions. A major challenge is to couple the alterations in the macroscopic structural or material characteristics of cells, spheroids and tissues, such as cell and tissue phase transitions, to the microscopic interferences of their elements. Therefore, the biophysical technologies need to be improved, advanced and connected to classical biological assays. In this review, the viscoelastic nature of cytoskeletal, extracellular and cellular networks is presented and discussed. Viscoelasticity is conceptualized as a major contributor to cell migration and invasion and it is discussed whether it can serve as a biomarker for the cells’ migratory capacity in several biological contexts. It can be hypothesized that the statistical mechanics of intra- and extracellular networks may be applied in the future as a powerful tool to explore quantitatively the biomechanical foundation of viscoelasticity over a broad range of time and length scales. Finally, the importance of the cellular viscoelasticity is illustrated in identifying and characterizing multiple disorders, such as cancer, tissue injuries, acute or chronic inflammations or fibrotic diseases.

## 1 Introduction to the Phenomenon of Viscoelasticity in Cells

The phenomenon of viscoelasticity can be found in nature in several kinds of material, such as in the most prominent example, rubber, and can be employed in engineering of synthetic or biological materials. A material behaves elastically according to Hooke’s law if the applied stress is proportional to the strain generated in the material, which is true up to a certain level of stress. No material behaves exclusively in a purely elastic manner, but many of these materials can be modeled as one elastic material, which is especially the case when the strain is small. A key assumption in an exclusive elastic material is that the energy required for deformation is stored in the material and is released with its entire efficiency when the material returns to its initial state after deformation. Viscoelasticity is by definition a state of thickness or toughness based on the internal structure of the material. In viscous materials, which include liquids, this means that the energy required to deform it, is partly converted as heat, which is related to internal structural losses. In viscous materials, the force is proportional to the amount of change in deformation. It is generally accepted that some materials can be assumed to be purely elastic or purely viscous. Viscoelasticity can therefore be defined as a property of a material that has both an elastic and a viscous component.

In biology, viscoelasticity refers to the property of living matter, including cells, spheroids, and tissues, that manifest both viscous and elastic properties when subjected to deformation. In the past, it was often assumed that cells behave in a purely elastic way, but this often turned out to be a too simplistic view of things. However, although cells are viscoelastic, it is not always straightforward to evaluate or simulate viscoelasticity, and adopting a simplified elastic behavior of cells for this purpose may be a reasonable alternative. The viscoelastic or material characteristics of cells, spheroids and tissues act as major regulators of cell and tissue growth, cell motility, and tissue homeostasis (Barriga and Mayor, 2019; Burla et al., 2019; Petridou and Heisenberg, 2019; Chaudhuri et al., 2020). Biological systems, including active matter, exhibit viscoelasticity, which allows them to maintain a fundamental architectural structure owing to their solid-like nature, but at the selfsame time to dynamically rearrange themselves into various conformations and modes owing to their viscous nature (Lecuit et al., 2011; Clément et al., 2017; Pegoraro et al., 2017; Petridou and Heisenberg, 2019). At the cellular length scale, viscoelasticity affects various single-cell features such as conformation, division, and movement, and is primarily governed by the physical characteristics of the supporting cytoskeletal systems (Pegoraro et al., 2017). Viscoelasticity at the tissue length scale has been shown to play an integral role in collective morphogenetic events such as tissue involution, spreading, injury repair, and migratory processes, and is primarily governed by the concert of cell-cell and/or cell-extracellular space interfaces (Petridou et al., 2017; Barriga and Mayor, 2019; Chaudhuri et al., 2020).

Cell, spheroids and tissues possess a multitude of inelastic characteristics, among them are viscoelasticity, plasticity and fracture. When mechanically deformed, it is usually impossible to avoid unfolding protein domains, unbinding cytoskeletal crosslinkers, dividing organelle assemblies, breaking weak sacrificial bonds, disrupting transient cell-matrix and cell-cell adhesions and inducing gene expression through translocation of transcription factors into the nucleus. All of which are regarded as inelastic. These structural transformations are mostly reversible and consequently not plastic in the strict meaning of the word, however they dissipate significant quantities of elastic energy through structural attenuation (Gralka and Kroy, 2015). The inelastic reaction can be observed as gradual or partial recovery of the material after elimination of the forces that caused the deformation (Gavara et al., 2008). Besides, it is rational that the deformation is considered to be a function of the history of the exerted forces. Viscoelastic materials display three prominent characteristics: stress relaxation, which is the stress decreases with time (a response of a viscoelastic material to a constant strain step), creep (a constant stress with a decrease in strain with time), and hysteresis (an incongruence between loading and unloading processes) (Banks et al., 2011). The term “viscoelasticity” combines both types of mechanical response, the response of elastic solids and viscous fluids. Consequently, not merely solids, but also liquids are susceptible of displaying such a property. Yet the manner in which they react to a mechanical cue differs widely. The response of a fluid to a given deformation under any two conditions would be identical, whereas a solid, for example, would respond differently in its original form and after a deformation. More generally, for solids, pure strains can affect the reaction of the material, while rotations can have no effect (Truesdell et al., 2004). When addressing viscoelasticity, it has to be taken into account that the inelastic response is also present (Trepat et al., 2007), even though the response may not really be permanent or irreversible and can also be reversible plastic (Gralka and Kroy, 2015). Moreover, viscoelastic characteristics of cells have been proposed to play a key role in the regulation of cellular functions, such as motility (Barriga and Mayor, 2019; Burla et al., 2019; Petridou and Heisenberg, 2019; Chaudhuri et al., 2020). Viscoelastic characteristics of cells are proposed to act as critical new biomarkers of disease status and advancement (Bao and Suresh, 2003). The most straightforward attempt to specify the viscoelastic characteristics of cells focuses on two variables: Stiffness and viscosity, which typify the elastic and dissipative elements of a cell’s reaction to stress (Moeendarbary and Harris, 2014). Elastic reaction has been widely adopted as a biomarker for cancer cells (Cross et al., 2007) or metastatic capacity (Xu et al., 2012). In addition, elasticity has been implicated in cell migration patterns occurring in embryogenesis (Barriga et al., 2018). Cell viscosity has been associated with numerous biological events, including diffusion (Wojcieszyn et al., 1981), porous traffic and deformability of erythrocytes (Lim et al., 2006) and the state of cellular disease (Eze, 1992; Zakim et al., 1992). Therefore, it can be hypothesized that the viscoelastic properties of cells may serve as a novel biomarker for cellular motility and thus for the progression of diseases such as cancer metastasis, in which cell invasiveness plays a key role.

Following the introduction and explanation of the phenomenon of viscoelasticity, the review consists of presenting and discussing biophysical techniques for determining viscoelasticity. The review pursues a hierarchical structure that reflects the spatial scale, ranging from firstly molecular mechanosensing between cells and cell matrix, to secondly transcriptional regulation leading to thirdly changes in cell state, such as epithelial to mesenchymal transition (EMT), encompassing single cell migration, to fourthly multicellular processes, including collective migration, spheroid and tumeroid biology, and fifthly disease states such as cancer, fibrosis, and others.

## 2 Biophysical Techniques for Analyzing Viscoelasticity

### 2.1 Classical Biophysical Techniques to Analyze Viscoelasticity

**FIGURE 1:**
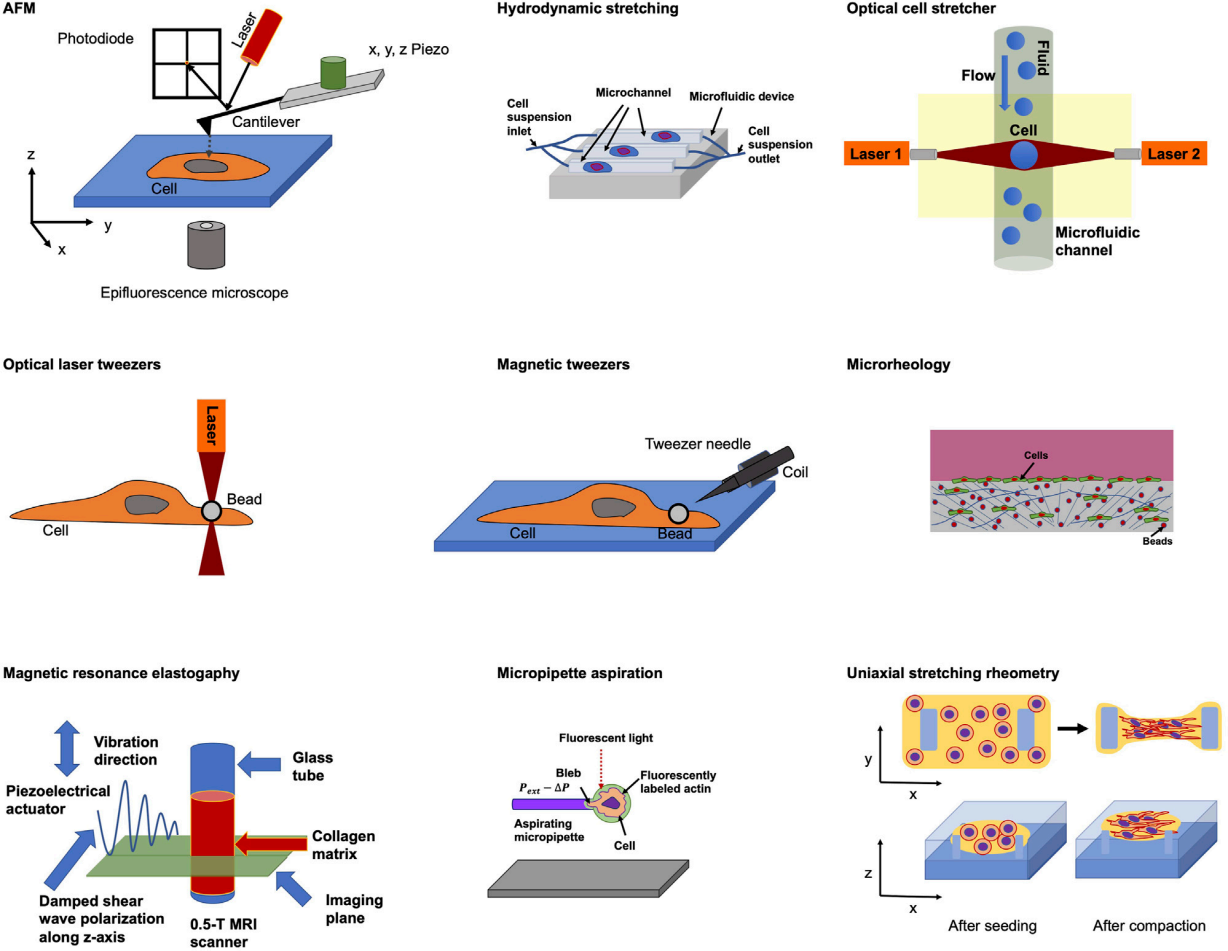
Selected biophysical techniques for probing cellular mechanical characteristics.

The majority of approaches to query the viscoelastic behavior of cells utilize forced induced deformations or probes (Wirtz, 2009) on relatively short time scales due to experimental requirement and feasibility. Efforts to gauge the elastic aspect of cell viscoelasticity involve atomic force microscopy (AFM) (Haase and Pelling, 2015; Fischer et al., 2017, Mierke et al., 2017, Fischer et al., 2020), hydrodynamic stretching (Gossett et al., 2012), optical cell stretcher (Kunschmann et al., 2017, Kunschmann et al., 2019; Mierke et al., 2020), optical laser tweezers (Lincoln et al., 2004), magnetic tweezers (Amblard et al., 1996; Aermes et al., 2020, Aermes et al., 2021), microrheology (Mason and Weitz, 1995; Crocker and Hoffman, 2007), magnetic resonance elastography (Muthupillai et al., 1995; Zhang et al., 2021), micropipette suction (Hochmuth, 2000) and uniaxial stretching rheometry (Desprat et al., 2005) (Figure 1). The viscous part of the reaction of cells to mechanical probing has been determined employing biophysical techniques encompassing microrheology (Mason and Weitz, 1995; Crocker and Hoffman, 2007), micropipette suction (Hochmuth, 2000), fluorescent rotor protein (Kuimova et al., 2008), AFM (Rebelo et al., 2013), electronic spin resonance (Mastro and Keith, 1984) and optical laser tweezers (Ługowski et al., 2002) (Table 1).

**TABLE 1:** Most important characteristics of specific biophysical techniques.

Biophysical technique	Characteristics	Advantages	Disadvantages	Test material	1D, 2D or 3D
Quantitative phase imaging (QPI)	Tracks	Probe- and contact-free	Excitation/emission are time limited (photobleaching), can reduce cell viability and stress the cell physiology (photoxicity)	SC	2D
	Displacement of cells due to shear flow to assess cell stiffness	Fluctuations		CC	3D
	Measurement of biomass movement	Unlabeled specimens		SP	
	Viscoelasticity	Low phototoxicity		OR	
	Contrast agents are inserted into the cell (dyes, nanoparticles) or induced via genetic mutation	No photobleaching		TS	
	Cell membrane fluctuations	Objective measure of morphology and dynamics, free of variability due to contrast agents			
Quantitative phase rheology (QPR)	Viscoelasticity	Probe- and contact-free	Flat substrate	SC	2D
	Shear stiffness measurement	Single-shot imaging of cellular rheologic properties		CC	3D
		Fluctuations			
Atomic force microscopy (AFM)	Indents cellular surface with a micron-sized bead attached to a cantilever while the motion of the cantilever is monitored by an optical sensor	Probing of specific receptors of cells with coated cantilevers	Low throughput	SC	2D
	Measurement of molecules, cell organoids and cells to whole tissue	Adherent and suspended cell measurements	Cells needs to be on the surface	CC	3D
	Elasticity	Relatively low throughput		SP	
	Viscoelasticity	1 cell per min		OR	
				TS	
Hydrodynamic stretching	Microfluidic chip in which optical fibers have been arranged in a post-processing step	High throughput	Without 3D matrix environment	SC	1D
	Microscopically observed cell transit through microchannels or pores	Capable of probing single-cell deformability at approximately 2,000 cells/s	Flow or liquid effect		
	Elasticity	Label-free	Cell size and adhesiveness can contribute to the measurement		
	Viscoelasticity	Computationally analyzed to extract quantitative parameters			
Optical cell stretcher	Optically deformation of the whole cell	Relatively high cell numbers	Single cells or small cell cluster without 3D matrix environment	SC	2D
	Viscoelasticity	1 cell per min	Rounded cells without protrusions can be analyzed	CC (small SP)	
		Label-free	Cells with dark granules, such as melanoma cells cannot be analyzed		
		Constant, gradient and oscillating forces	Heat may affect measurements		
Optical laser tweezers	Indentation using an optically trapped bead	Relatively high cell numbers	Single cells or small cell cluster without 3D matrix environment	SC	2D
	Elasticity		No generation of a 3D force trap	CC	3D
	Viscosity				
Magnetic tweezers		Relatively high cell numbers	Cells need to be on the surface	SC	2D
		0.5–1 cell per min	Bead internalization may impact measurements	CC	
		Live cells without resorting to intense irradiation, can be easily multiplexed	Cell movements not allowed during measurements		
Microrheology	Elasticity	Time-dependent	Limited by modeling accuracy	SC	2D
				CC	
	Viscosity	Frequency-dependent	Computationally expensive modeling	SP	3D
				OR	
				TS	
Micropipette suction	Suction is employed to draw a cell into a confined space while tracking its surface displacement	Low throughput	Without 3D matrix environment	SC	2D
			Low throughput	CC	
Uniaxial stretching rheometry	Creep function of a cell stretched between two glass plates is measured after exertion of a constant force step	Constant forces and oscillating forces	Cell may rearrange during measurement	SC	2D
				CC	
				SP	
Fluorescent rotor protein analysis	Non-mechanical determination of fluid viscosity	Molecule-specific	Fluorescence activation of these fluorescent probes is an irreversible process	SC	2D
				CC	3D
	Fluorescence lifetime imaging	Rapid live-cell imaging	Construct operative fluorescence-switchable probes for analysis inside living cells need to be designed	SP	
				OR	
	Viscosity			TS	
Electronic spin resonance	Characterizes the spectral shape change induced by shear stress	Fast	Au nanoparticles	SC	2D
				CC	3D
				SP	
	Ratio of the internal to the external viscosity, and elastic property of the cell membrane		Shear flow measurements	OR	
				TS	
Elastography/Viscoelastography	Elasticity	Time-dependent	Quantitative values of the storage and loss moduli and/or parameters of a fitted rheological model	OR	2D
Magnetic resonance elastography (MRE)	Viscosity	Frequency-dependent		SP	3D
	Measurement of tissues			TS	
	Actuation introduces disturbance to the tissue				

SC, single cells; CC, cell clusters; OR, organoids; SP, spheroids; TS, tissue specimen; 1D, one dimensional; 2D, two dimensional; 3D, three dimensional.

An overview of these selected biophysical techniques and their characteristics are provided in (Table 1), which also lists major advantages and disadvantages. The Table 1 also summarizes whether a specific technique has been applied to a 3D model, such as spheroids and organoids, or tissue specimen. Most of the biophysical techniques, including QPI, AFM, Microrheology, Fluorescent rotor protein analysis, Electronic spin resonance and MRE have been adapted to measure additionally 3D models, or tissues. In the following, selected prominent major biophysical techniques are briefly introduced and their potential for probing viscoelasticity is highlighted. Moreover, it is pointed out whether a biophysical technique is suitable for employment in a 1D, 2D, 3D model system and/or tissue specimen (Table 1). Specifically, Optical cell stretcher, Magnetic tweezers, Micropipette suction and Uniaxial stretching rheometry can solely been used in 2D model systems (Table 1) and are most often utilized to determine the mechanical characteristics of single cells, including viscoelasticity.

The measurement of the viscoelastic characteristics of active matter, such as cells, spheroids and tissues, has expanded in the recent years, since it has become a research focus to seek for a correlation of viscoelasticity and the state of disease, differentiation of cells, or the transformation of cells or collections of cells (Darling et al., 2008; Lekka et al., 2012; Rother et al., 2014; van Zwieten et al., 2014). Therefore, AFM turned out to be a suitable technique for probing cell mechanics, including viscoelasticity in 2D and 3D model systems. However, AFM indentation continues to be a favorite method for exploring the nanoscale characteristics of soft samples such as cells, spheroids and tissues (Hutter and Bechhoefer, 1993; Gibson et al., 2005; Engler et al., 2009; Gavara, 2016). In AFM, the elastic characteristics of living cells are generally assessed by force-displacement (F-Z) curves. The Hertz model or its variations are imposed on the approximation section of the F-Z curve to obtain the Young’s modulus, the elastic characteristic employed to define the mechanical performance of the specimen (Mahaffy et al., 2000; Pirzer and Hugel, 2009). Physical models such as these, however, hypothesize a purely elastic character of the specimen, whereas in fact the majority of biological specimens are viscoelastic. Viscoelasticity is evident in a marked hysteresis between the approach and retraction segments of the curves (Rebelo et al., 2013); the dependence of the indentation velocity on the E-Hertz values obtained from the force profiles with the Hertz model (Hertz, 1882); the force relaxation phenomena at constant indentation height and the creep at constant imposed force (Nawaz et al., 2012). If a standard F-Z curve might also be employed to quantify viscoelastic characteristics, it becomes feasible to adopt a standard method with properly quantified uncertainties (Sniadecki et al., 2007) for both viscoelasticity and elasticity determinations. Apparently, until now, this did not seem an option due to the absence of a mathematical/computational setting that enables post-processing of force-displacement measurements to exhaust the pertinent viscoelastic constitutive variables. A new methodology has been presented to derive the viscoelastic characteristics of soft specimens including cells, spheroids and tissues directly from standard AFM F-Z curves. This technique is founded on the well-established theoretical model pioneered by (Crick and Hughes, 1950) for the displacement constraint problem of a linear viscoelastic half-space through a rigid axisymmetric indenter under arbitrary load conditions. The Crick and Hughes model can be integrated with cognate numerical techniques to handle both approach and retraction phase datasets of the experimental F-Z curves (Efremov et al., 2017). The methodology is confirmed with finite element simulations, with experiments on polyacrylamide hydrogel specimens, and by benchmarking against in place AFM viscoelasticity measurement approaches.

Besides single biophysical technique approaches, a combined approach can be employed to further improve the mechanical characterization of cells. For instance, the pairing of an AFM with a confocal fluorescence lifetime imaging scanning microscope to examine the mechanical characteristics of individual adherent cells seems to be of high relevance in this context (Fuhrmann et al., 2011). As a result, force-indentation curves can be identified, and subsequently indentation depth-dependent elastic moduli have been obtained for several cell lines and different cell types. The stiffness tomograms show clear distinctions in the mechanical characteristics of the cell lines examined. This finding indicates that the microscopic interpretation of the enhanced compliance of cancer and pre-cancer cells might reside in their proneness to “crumble and yield” rather than in their capacity to “bend and flex.” Moreover, AFM is a high-performance method that can quantitatively map the mechanical viscoelastic characteristics in parallel with imaging the 3D topography of specimens. But it is restricted to adherent cells and can only measure from the upper surface of the cell. When oversimplified models are employed to derive an effective elastic modulus E_eff_, the complex geometry and characteristically very inhomogeneous nature of the cells are entirely discarded, and it is frequently challenging to reconcile the elastic response values obtained in this manner with those acquired in other kinds of measurements.

In addition to analyzing the mechanical properties of adhesive cells, certain biophysical techniques such as the optical cell stretcher technique can also be used to analyze the mechanical properties of non-adherent cells. Two decades ago, the optical cell stretcher was invented, in which two counter-propagating divergent laser beams are pointed at a cell (Guck et al., 2001, Guck et al., 2005). When both beams share the identical intensity, there is no net force exerted on the cell lengthwise down the optical axis. However, it emerges that in tandem with a lateral optical gradient force that holds the cell on the optical axis, a symmetrical pair of forces affects the cell surfaces that stretches the cell in the optical axis direction. The cells will be captured in the center of the stretcher, and by simply applying different laser powers, the forces applied to the cell surfaces resulting in longitudinal stretching can be adjusted. From such deformation profiles, an effective cellular compliance J can be deduced, including time-dependent characteristics. In view of viscous and elastic inputs, such measurements can be investigated using power law rheology models, a standard linear solid model, or a Burger model (Ekpenyong et al., 2012). Among the main benefits of the optical stretcher is the capability to couple it with microfluidic instruments and perform high-throughput measurements of cell compliance. Nevertheless, the distributions of optical deformability appeared to be so widespread that it was not practical to uniquely classify single cells (Guck et al., 2005). However this method is also suitable for a multitude of physiologically adherent and non-adherent cells, which can be performed on the one hand with the exclusion or on the other hand with the admixture of biochemical drugs that interfere with cell mechanics (Chan et al., 2015; Kunschmann et al., 2017, Kunschmann et al., 2019; Mierke et al., 2017, Mierke et al., 2020). In addition to the optical deformation of a cell, an important thermal impact on the specimen also arises, which permits the induction of fast temperature variations (Kießling et al., 2013). Leveraging this thermal phenomenon, optical stretching consequently facilitates a new category of investigations referred to as “thermorheology,” which extends beyond simple mechanical stretching.

Another biophysical technique to probe the mechanical properties of adhesive cells, spheroids and tissues is magnetic tweezers. A totally different geometric attempt to investigate cells physically also leverages the interplay of magnetic particles with an external magnetic field. Perhaps the earliest record of the basic principle of magnetic tweezers brought to bear on biological systems was a 1950 experiment by Crick and Hughes, which it then referred to as the magnetic particle method (Crick and Hughes, 1950). In specific, cultivated chicken fibroblasts have been induced to phagocytose ferromagnetic particles. After imposing a magnetic field, three different types of movements of these particles could be perceived as twisting, pulling and pushing. These results indicate that the cytosol of cells is not only viscous but also possesses elastic characteristics. A crucial element in these experiments is the accurate calibration of the forces exerted on the typically superparamagnetic beads in the external rather less homogeneous magnetic field. Accurately monitored forces of up to 10 nN on 4.5 μm sized beads have been achieved and the mechanical response of adherent cells to the magnetic stimulation of the beads bound to plasma membranes has been determined (Bausch et al., 1998, Bausch et al., 1999).

Subsequently, superparamagnetic beads coated with integrin ligands have been employed to mechanically explore the coupling strength between the extracellular matrix scaffold and the cytoskeleton (Kollmannsberger and Fabry, 2007; Kollmannsberger et al., 2011; Mierke et al., 2017). Viscoelastic behavior can be determined by performing these creep experiments. However, the majority of experiments have been performed under 2D settings, where the cells adhered to a flat substrate, which can be coating with extracellular matrix protein for supporting cell adhesion and spreading. It is crucial in this experiment, however, to bring the magnet extremely close to the probe, which can be difficult for cells in 3D microenvironments or for tissues. Another disadvantage is that only adhesive cells can be measured and non-adhesive cells are generally excluded.

Nevertheless, it has been observed that these biophysical analyses may be heavily biased through the precise region of a cell being probed (Haga et al., 2000), changes in the cytoskeletal framework due to an imposed stress (Reed et al., 2008) or cell specific interactions occurring to a probe (Squires and Mason, 2010). All these interferences can distort determinations of cell viscoelasticity. Therefore, it was of great interest to develop other biophysical techniques that circumvent these shortcomings. A novel approach for developing such as biophysical measurement technique is presented in the following subsection.

### 2.2 Novel Contact Free Measurement Technique of Viscoelasticity

To determine stiffness, standard techniques (Table 1) involve the employment of cell contact or invasive probing and are therefore low throughput, work demanding, and constrained by placement of the sensor. Based on these findings, it seems to be obvious that another high throughput, less work load-based and sensor-independent technique needs to be engaged. Quantitative phase imaging (QPI) provided this, offering a probe- and contact-free approach to quantify variations, such as fluctuations, in the viscoelasticity of cells. In specific, QPI readings exhibit a pronounced underdamped reaction to temporal variations in the allocation of cell biomass (Nguyen et al., 2020). The effective stiffness and viscosity data obtained from these oscillations of the cell biomass mass distributions are related to the effective cell stiffness and viscosity determined with AFM. This finding is consistently true for different cell types with varying levels of cytoskeletal perturbation and throughout the epithelial to mesenchymal transition (EMT) of individual cells. Consequently, the QPI can be used to reliably and quantitatively determine the viscoelasticity of cells (Nguyen et al., 2020).

A QPI-based approach to accurately determine the viscoelastic properties of cells that is non-contact and non-invasive has been developed as a new technique referred to as quantitative phase rheology (QPR). Specifically, QPI (Popescu and Park, 2015) generally represents a microscopic approach to assess the phase shift or delay of light resulting from its interference with the relative dry matter or non-aqueous matter of a cell’s biomass (Zangle and Teitell, 2014). By means of an experimentally ascertained cell-average specific refractive index, the phase shift of the light can be correlated with the biomass of the cell (Barer, 1952; Davies and Wilkins, 1952). The growth of cells (Mir et al., 2011), cell death (Pavillon et al., 2012), and reactions to growth impairment through chemotherapeutics or targeted inhibitory substances of classical biological processes (Reed et al., 2011; Mir et al., 2014; Hejna et al., 2017) have been analyzed with QPI.

Moreover, previously QPR has been applied to determine the viscoelastic properties of the membrane of enucleated erythrocytes, that incorporated the design of an analytical model connecting the vibrational modes monitored with the viscoelastic characteristics by autocorrelation of the quantitative phase data (Popescu et al., 2008; Park et al., 2010). Since this model has been developed for enucleated cells, it may not be directly transferable toward the intricate complex nucleated cells. In applying the model to nucleated cells, spatial and temporal autocorrelations of quantitative phase data derived from colonies of human pluripotent stem cells evidenced both a more substantial amount of spatial coordination and a quicker rate of temporal decorrelation for pluripotent cells when compared to their more differentiated progeny (Zangle et al., 2013). More recently, it has been found that spatial autocorrelations of quantitative phase data provide an index of intracellular disarrangement of cells, a factor that is linked to cell stiffness induced in reaction to deformation caused by fluid shear (Eldridge et al., 2017). Still more work on QPR highlights that the temporal autocorrelation of quantitative phase data is associated with cellular transport behavior, encompassing diffusion (Wang et al., 2011; Ceballos et al., 2015; Kandel et al., 2017), and indicates a correlative association with cellular stiffness (Ma et al., 2016). At present, however, there is to my knowledge no other QPR technique (Nguyen et al., 2020) that can simultaneously model and accurately measure both the elastic and viscous moieties of cell viscoelasticity. More specifically, the autocovariance in time of the quantitative phase data for cells in the interphase of the cell cycle has been revealed to behave in a manner similar to a mass-spring-damper system (Nguyen et al., 2020). The elastic and viscous parts that characterize this behavior are linked to the viscous and elastic stiffness elements of interphase cells, which have been revealed through AFM analyses. To determine whether the mechanical properties rely on the actin cytoskeleton, the stiffness of three different cell types has been altered through addition of a pharmacological drug, such as cytochalasin B (Petersen et al., 1982), which impairs the polymerization of actin. Indeed, there is a high degree of correspondence between QPR outcomes and AFM viscoelasticity readings. To confirm that stiffness has a physiological function in cells of the same genetic origin during a cell state transition, QPR is applied to a cellular model of EMT (see also below) (Zhang et al., 2014). The hypothesis is supported by a novel EMT-based gene signature, encompassing ITGAV, DAB2, SERPINE1, MATN3, and PLOD2, that has been identified recently for gastric cancer (Dai et al., 2021). In line with expectations, the QPR measurements of stiffness and viscosity are related to EMT status (Nguyen et al., 2020). In addition, the results indicate that label-free QPR can be utilized to evaluate cell stiffness and viscosity, which confers an advantage over conventional biophysical techniques for examining the mechanical characteristics of cells and thus greatly broadens the use of QPI to monitor cell performance (Table 1).

Specifically, the locomotion of the cell biomass, when quantified as the autocovariance of the quantitative phase image measurements, exhibits a harmonically oscillatory movement. The oscillation and decay of the autocovariance can be addressed by employing a two-parameter viscoelastic model. The fitting of this model to empirical data permits the estimation of separate quantities for the effective stiffness and viscosity of a cell. However, earlier methods exist for measuring stiffness (Eldridge et al., 2017) using QPI datasets. The temporal measurements of the cell biomass movement (Nguyen et al., 2020) and other rheological features of a cell (Wang et al., 2011; Ceballos et al., 2015; Eldridge et al., 2017; Kandel et al., 2017) by QPI is what will be denoted as QPR.

To differentiate among various cell types, states, and operating conditions using the QPR measurements, a uniform stiffness over the measurement interval of approximately 5 h has been assumed. However, this hypothesis is not valid in mitosis, when the stiffness of the cell undergoes a fundamental alteration (Stewart et al., 2011). Hence, dividing cells need to be excluded from the analysis. In specific, an automated technique for identifying cell divisions has been developed to eliminate them from the quantitative phase image data (Nguyen et al., 2020). Thus, QPR can automatically handle QPI data from living cells and provide rheological characteristics of cells. Future efforts should be placed on the improvements in the spatial and temporal resolution of QPR that are necessary to assess the magnitude of viscoelastic alterations of cells throughout mitosis. These data may then aid to understand the functional role of cell mechanics in this specific process.

QPR and AFM measurements provide cellular viscoelasticity within a similar force and time regime. An interpretation of the experimental data can be provided by a model presented by Qian (Qian, 2000) that has been developed for the tracking of single particles located inside a Kelvin-Voigt material. Qian’s model offers comparable forecasts to the well-known classical series spring-damper-based Maxwell material model that can be performed on QPR measures. The Maxwell material model can be employed when the track shifts of small particles of cell biomass are effectively presumed when embedded in a Maxwell material. A moderate fit to AFM viscosity values (*R* ^2^ = 0.81) can be attained when applying the Kelvin-Voigt model to QPR data sets relative to the fitting of the Maxwell model (*R* ^2^ = 0.89). This implies that a Maxwell material model appears to be the more suitable two-parameter linear viscoelastic material model for the purpose of analyzing the QPR results. While this simple linear two-parameter model is a somewhat streamlined perspective on cell viscoelasticity, this model still accurately grasps the key attributes that have been delineated in the data.

Although this physical explanation of the mathematical model contains an inertia expression, it still reflects the response of a fluid exhibiting a low Reynolds number. This phenomenological hypothesis permits fitting a two-parameter viscoelastic model and deducing the rheological features of the cells based on the QPI data, which correspond to the AFM results. With respect to a possible physical significance of this expression, it has been demonstrated that inertia-like oscillations can arise in actively propelled, viscoelastic fluids (Berner et al., 2018). Since the cell remains an active composite (Chen et al., 2018), it can be hypothesized that the inertia-like performance exhibited in this system may result from a close association between viscoelastic material characteristics and active force generation due to cytoskeletal reorganizations. This points to the requirement for future models built on a superior cellular material model that can more fully accommodate these cellular mechanical characteristics.

Notwithstanding the fact that there is strong degree of correlation between AFM and QPR measures of stiffness and viscosity, a major disparity remains in the magnitude of these scores. Part of this discrepancy is accounted for by probe size disparities. Specifically, the AFM probe tip radius is 500 nm, while the effective probe for QPR involves the stuff inside the cytoplasm. The noted variation in the magnitude of the QPR stiffness in comparison to the AFM stiffness value is approximately 10^4^, which indicates a QPR probe dimension of approximately 5 nm. In addition, the cell can be modeled as a strictly linear viscoelastic material, but in broad terms, the cell rheology is a function of the length scale, strain rate, and amount of imposed force, which may vary between these two different kinds of approaches. In this way, AFM determines the viscosity from viscous displacement, while QPR provides an effective coefficient of friction that a particle experiences due to the viscosity of a cell. From a technical point of view, these are two distinct characteristics that are intimately linked by viscosity. The QPR approach is closest to passive particle tracking in microrheology (Weihs et al., 2006), which yields a stiffness value derived from the anticipated response to passive particle motion. The basic equations for the input and output factors of these three biophysical techniques are discussed briefly in the following. Fluorescently labeled sensor beads are embedded in a material, such as cells, and their Brownian motion is recorded with video microscopy. To link this particle movement to the rheological characteristics of the material, each sensor particle is monitored. After recording the movement of the sensor particles, the locations of the particles in each image are mapped according to their brightness-weighted centroid and then associated to form trajectories. The composite-averaged mean-squared displacement (MSD, $\langle \Delta r^{2}\left(\tau \right)\rangle$ is computed based on these particle trajectories. The MSD averaged over the entire composite provides a quantification of the particle movement and therefore reveals insight into the rheology and physical condition of the material, such as a cell. The particle movement is relatable to rheological characteristics, for example the creep compliance J(τ) , applying the generalized Stokes-Einstein relationship (GSER). The GSER is given in Eq. 1:

$$\langle \Delta r^{2}\left(\tau \right)=\;\frac{k_{B}T}{\pi a}\;J\left(\tau \right)$$ (1)

where *τ* stands for the lag time, *k*_*B*_ *T* denotes the thermal energy, and *a* indicates the radius of the particle.

The GSER is applicable in cases where several conditions are fulfilled: firstly, the length of the probe particle is much longer than the characteristic length span of the material, secondly, effects of inertia on the sensor probe and the fluid are ignorable, and thirdly, the length compression factor of the fluid is ignorable (Squires and Mason, 2010; McGlynn et al., 2020). AFM techniques evidenced that the elastic modulus of the lower invasive epithelial bladder cancer cells RT112 displayed a plateau modulus at the slower frequencies, which is not the case for the two other stronger invasive epithelial bladder cancer cells T24 and J82, implying that the invasiveness renders the cells to be less elastic (Abidine et al., 2021).

When the applied force is less than 3 nN to stay in the linear elastic range, the Hertzian model can be employed. When the tip of the cantilever comes into physical contact with the specimen, the force imposed on the cantilever rises to a pre-selected nominal value $F_{0}$ , that is equivalent to an initial indentation $\delta_{0}$ . The correlation is provided by Sneddon’s modification of the Hertzian model of contact mechanics, as indicated in Eq. 2 below:

$$F_{0}=\;\frac{3E\tan\text{Θ}}{4\left(1-\nu^{2}\right)}\;\delta_{0}^{2}$$ (2)

*E* denotes the Young’s modulus of the cell, ν its Poisson ratio of approximately 0.5 and Θ=20° represents the half pyramid angle. $\delta_{0}$ is selected in such a way that the indentation depth of the tip into the specimen is sufficiently deep to have a suitable contact area and never too deep to stay within the range of linear elasticity.

Moreover, microrheology data (Wirtz, 2009) compared to AFM data (López-Fagundo et al., 2016) on mouse embryonic fibroblasts reveal large discrepancies in the magnitudes measured: 14 Pa for microrheology stiffness compared to 7.7 kPa for AFM stiffness. A similar mismatch between AFM stiffness and microrheology stiffness values has also been identified for MCF10A mammary epithelial cells (Li et al., 2009).

Collectively, these findings demonstrate the inherent capability of a label-free and non-contact technique that can accurately gauge the rheological behavior of cells. As a transmitted light microscopy approach, QPI is non-invasive and therefore it minimizes the interfering impact of probes in the examination of biological events in living cells. QPI can be deployed to obtain a measure of the biomass distribution of cells over time. Notably, the autocovariance of this biomass distribution over time, referred to as Cφφ, is calculated to quantify variations in the biomass distribution driven by the movement of cell structures or compartments, such as organelles.

To assess the resemblance of the quantitative phase data over time, an unbiased estimation of the autocovariance of the phase shift signal can be employed, representing an autocorrelation of the data that have been subtracted from the mean. The temporal autocovariance is standardized to the total amount of data points taken in each autocovariance window, with respect to the end of the time shift window $t_{0}$ , and defined in Eq. 3:

$$C_{\varphi \varphi }\left(x,y,t_{0},\tau \right)=\;\frac{w\sum_{j=0}^{w-\tau /\text{Δ}t}\left(\varphi \left(x,y,t_{o}-j\text{Δ}t\right)-\varphi \left(x,y,t_{0}\right)\right)\;\left(\varphi \left(x,y,t_{0}-j\text{Δ}t-\tau \right)-\varphi \left(x,y,t_{0}\right)\right)}{\left(w-\frac{\tau }{\text{Δ}t}\right)\sum_{j=0}^{w-\tau /\text{Δ}t}{\left(\varphi \left(x,y,t_{0}-j\text{Δ}t\right)-\varphi \left(x,y,t_{0}\right)\right)}^{2}}$$ (3)

Additionally, under the assumption of damped oscillations that rely on a series of harmonics a and b can be described as in Eqs 4, 5, respectively:

$$a=\;\frac{k}{2\mu }$$ (4)

$$b={\left(\frac{k}{m}\right)}^{1/2}{\left(1-\frac{km}{4\mu^{2}}\right)}^{1/2}$$ (5)

wherein *k* denotes the effective spring constant of the cell sensing the particle over the measurement interval, *μ* stands for the effective damping coefficient resulting from the viscous forces of the cell sensing the particle, and m represents the average biomass of the particles in the system. The autocovariance equation can be broken down and consequently, the effective stiffness can be presented by the relation of the fitting coefficients as denotes in Eq. 6:

$$\frac{k}{m}=\;a^{2}+\;b^{2}$$ (6)

Since QPR relies on an established quantitative phase imaging workflow, QPR can be amenable to full incorporation into other types of measurements that are prevalent in quantitative phase techniques, including cell biomass or biomass accumulation rates.

## 3 Molecular Mechanosensory Behavior

The molecular mechanosensory behavior of cells relies firstly on their interplay with neighboring cells and secondly on their interaction with the extracellular matrix environment. Cells must perceive their environment and be able to react to changes. Thereby, the cells can even adapt their cellular functions, such as their adhesion and migration capacity. Mechanical cues can be perceived by cells through a wide variety of membrane-anchored receptors, comprising stretch-activated ion channels, integrins, cell membrane-spanning G-protein-coupled receptors and cadherins (Paluch et al., 2015). The integrin- and cadherin-based adhesion complexes form at the contact interfaces between cells and extracellular matrix and between cells and cells, accordingly. Both involve proteins that respond to alterations in tensile forces and adjust their molecular composition and dynamics in reaction to these forces, leading to biochemical signaling effects that relay the mechanical input (Han and de Rooij, 2016; Martino et al., 2018). Multiple integrins join together to form adhesion complexes known as focal adhesions, which transfer mechanical forces bidirectionally across the extracellular matrix and the intracellular actomyosin cytoskeleton (Geiger and Yamada, 2011; Jansen et al., 2017). The involvement of integrins (particularly integrin α5β1) in focal adhesions during mechanotransduction is clearly evident (Mierke et al., 2011; Sun et al., 2016; Jansen et al., 2017; Elosegui-Artola et al., 2018). Moreover, these mechanical signals govern a variety of cellular processes that exploit a repertoire of mechanosensors converting forces into biochemical pathways with mechanotransduction (Box 1).

BOX 1 **Definitions and Terminology.**

**Cytoskeleton** = A scaffold of biopolymer fibers that fill the entire cell. It is the main contributor of the material reaction of the cell that is deformed or under stress.
**Compliance (J)** = The relative degree to which a body yields to deformation by a force, typically sampled as time-dependent strain divided by constant stress.
**Deformation (of cells)** = Cells possess the ability to change their shape in response to mechanical stress by remodelling their shape.
**Durotaxis** = refers to the directional cell movement in which cells detect a gradient of varying stiffness (rigidity) in their microenvironment and preferentially migrate toward the stiffer extracellular matrix.
**Extracellular matrix scaffold** = The extracellular matrix represents a non-cellular moiety that forms the material support backbone for cellular elements. Beyond its structural nature, it has an important, active function in morphogenesis, differentiation, and homeostasis.
**Elastic modulus** = Otherwise referred to as the Young’s modulus, the E value provides a quantification of the strain reaction to a uniaxial stress in the direction of that stress in the linear range.
**Elasticity** = The characteristic of a material to deform to a certain degree under the action of a force and to recover its initial shape after the force has been withdrawn. Elasticity in itself is not a measurement of stiffness; what is generally intended is the elastic modulus, the ratio of stress to strain for a completely elastic solid.
**Entanglement (of fibers)** = Polymeric fibers can be wrapped around one another without being physically connected.
**Hysteresis** = The term describes the dependence of the state of a system on its history.
**Jamming** = Material viscosity becomes more divergent as the particle density rises.
**Linear elasticity** = The Young’s modulus or shear modulus is constant over spectrum of strains; thus, the stress is proportional to the strain.
**Mechanotransduction** = It is a process in which cells sense and react to mechanical cues by converting them into biochemical cues that drive specific cellular reactions.
**Non-linear elasticity** = Young’s or shear modulus that alters due to strain.
**Phase transition** = Macroscopic alteration of the characteristics of a system (order parameter) when a parameter exceeds a specific critical level (control parameter), also known as a critical point.
**Poroelasticity** = Investigation of the interaction of a porous elastic array with an interpenetrating pore fluid in a poroelastic material.
**Strain** = The value denoted by γ quantifies the deformation of a body. Specifically, it provides a quantification of the relative displacements of components of the body that are not accounted for by the movements of the rigid body. (unitless parameter that quantifies the amount of deformation after the exertion of stresses).
**Strain (longitudinal)** = represents the fractional alteration in length or elongation: *ε* = *δ*/L
**Strain stiffening response** = The nonlinear reaction of multiple biomaterials is the rise in stiffness with the augmentation of strain.
**Stress** = Force is per unit area: *σ* = F/A, whereby the SI unit is N/m^2^.
**Tension** = is the pulling force transmitted axially by the means of a string or similar object.
**Viscoelasticity** = A combination of elastic and viscous responses to applied stress. Most biological materials are viscoelastic: when they are deformed, the degree of their resistance decays with time, usually to a stable baseline (viscoelastic solid) but sometimes to zero at long times (viscoelastic fluid).
**Viscosity** = The quantity denoted by η quantifies the flow of the material at a specific velocity under load (Measure of the resistance of a liquid to deformation as a reaction to a shear stress. Viscosity is the relationship of stress to strain rate of a fluid).
**Young**’**s modulus** = A constant that expresses the resistance of a material to deformation when stretched: E = *σ*/ε.

### 3.1 Molecular Mechanosensory Interplay Between Cells of the Same Cell-Type: Dynamics of Adherens Junctions and Cadherins

Within the major elements of adherens junctions and desmosomes are molecules of the cadherin family (Rübsam et al., 2018). Among them are cadherins of the classical-type, such as E-cadherin, desmoglein and desmocollin that can be found in epithelial cells (Neel et al., 2021) and clustered protocadherins with α , β  and γ subfamilies (Nicoludis et al., 2016). The latter play a role in neural adhesion and self-recognition. Classical cadherins, such as E-cadherin (epithelial cells) and VE-Cadherin (endothelialc cells) belong to transmembrane glycoproteins that possess an extracellular domain driving cell-cell adhesion through homophilic or heterophilic interference and an intracellular domain regulating signal transduction cascades associated in a variety of cellular functions and processes, encompassing polarity, and gene expression (Meng and Takeichi, 2009; Liu et al., 2010; Baum and Georgiou, 2011; Maruthamuthu et al., 2011; Leckband and de Rooij, 2014; Ravasio et al., 2015). The positioning, adhesion strength, and distribution of adherens junctions relies on the specific context, such as the specific cadherin related to a distinct cell type. For example, E-cadherin in adherens junctions of epithelial cells, VE-cadherin in adherens junctions of endothelial cells and N-cadherin in adherens junctions of multiple cells, including cardiac muscle cells and mesenchymal cells. The cell adhesion molecule N-cadherin represents a biomolecule marker for the EMT, which leads to the establishment of an aggressive and invasive cancer cell phenotype through a so-called cadherin-switch that is subsequently associated with a transformation of a non-motile (epithelial) to a motile (mesenchymal) phenotype (Wheelock et al., 2008; Gheldof and Berx, 2013). The earliest discovery of cadherins started in cultures of epithelial cells, where type-I cadherins, such as E-cadherin, exhibited cells of epithelial origin with strong adhesions and its expression has been governed by stalled rather than motile cellular phenotypes.

Due to the specific environment adherens junctions behave either highly dynamical or extremely stalled and these dynamical remodeling can be adapted at every single step of the assembly of adherens junctions. Adherens junctions assembly in three major steps. Firstly, cells attach to one another in an initial step where extracellular domains of cadherins are involved, with the type of cadherin being displayed by neighboring cells and facilitating the tethering strength of this particular step. A second step represents the lateral extension of a nascent adhesion that covers the additional engagement of cadherins to enlarge the interaction zone. A third step encompasses the stabilization of the adherens junctions, where the cytoskeletal activity of collectively migrating cells are tuned and guided (Cavey and Lecuit, 2009; Meng and Takeichi, 2009; Theveneau and Mayor, 2012; Barriga and Mayor, 2015). Whereas the type of cadherin, such as E-cadherin, VE-cadherin or N-Cadherin, exhibited by a specific cell type may be important in shaping the strength of adhesion in the initiation phase, the conversion of cadherins is essential in governing the dynamics and durability of lateral extension and stabilization of adherens junctions, and thus the lifetime and vigor of the connection. Cadherin levels, such as E-cadherin (epithelial state) and N-cadherin (mesenchymal state), can be regulated at the transcriptional scale through specific transcription factors, including members of the Snail, Twist, and ZEB families, as an element of a specific switching process referred to as EMT, and the conversion of these adherens junctions proteins is subject to post-translational modifications. Downregulation of E-cadherin results in loss of stable epithelial cell-cell adhesive junctions, such as adherens junctions, apico-basal cell polarity, and the architecture of epithelial tissue, which aids in the escape of cancer cells from the primary cancer hub (Perl et al., 1998; Kourtidis et al., 2017). In contrary to the anti-migration function of E-cadherin, N-cadherin confers increased migratory and invasive capacity toward cancer cells independent of E-cadherin expression (Hazan et al., 2004). Thus, it seems that the procurement of N-cadherin is a crucial pace in the metastasis of epithelial cancer and the advancement of the disease. In addition, the cadherin switch in collections of cells leads to an unjamming to jamming transition through the weakening of cell-cell interactions, such as adherens junctions (Ilina et al., 2020). There is also an E-cadherin-integrin crosstalk that govern the migratory capacity of cells, such as cancer cells (Canel et al., 2013), since the formation of an adhesome (Horton et al., 2015, Horton et al., 2016) may alter the E-cadherin-dependent cell-cell interaction and subsequently cellular locomotion. In addition to integrin-dependent migration, integrin-independent migration is also observed (Schmidt and Friedl, 2010). However, it can be assumed that both types of invasion are highly dependent on integrin-driven adhesion toward the extracellular matrix, whereas collective invasion also demands dynamically remodeled cell-cell adhesions, meaning that slackening of cell junctions is necessary for invasion to occur.

### 3.2 Molecular Mechanosensory Interaction Between Different Cell Types in Transmigration

#### 3.2.1 What Affects the Transmigration of Cells in a 3D System?

These materials are selected primarily for their semipermeable and size-excluding characteristics to limit or permit transmigration and cell-cell coupling (Casillo et al., 2017). Nevertheless, previous investigations have demonstrated that pore size, pitch, and orientation influence cell performance, encompassing extracellular matrix production and migration (Irvine et al., 2001). The extracellular physical arrangement of integrin ligands in patterns of clusters may aid in the bunching of bound integrins into clusters, thereby perhaps governing cellular responsiveness to a specific average quantity of a ligand within the extracellular environment (Irvine et al., 2002). The mechanism underlying this response is only partially identified (Allahyari et al., 2020). Specifically, the 3D scaffold of the extracellular matrix environment can affect the migratory capacity of cells, when the pore size, adhesiveness or matrix stiffness is altered independently of each other (Peyton et al., 2011). It has been found that the greatest chance of substantial cell migration through the pores appeared at an intermediate pore diameter, and not at the maximum pore diameter, when it surpassed the cell diameter. Importance of co-culture systems, barrier systems, and organ-on-a-chip investigations (Zervantonakis et al., 2012; Jeon et al., 2015) arises from their utility in disease models and drug discovery, and from insights into cell-cell interactions at tissue boundaries (Bhatia and Ingber, 2014; Chung et al., 2018). There are interactions of cells with their surrounding extracellular matrix that are based on the pure structure, but there are also indications that this is not enough, which means that 3D matrices are additionally crucial for cell sensing mechanisms and bidirectional cell-matrix interactions. In some investigations, the structural purpose has been focused. The purpose of one investigation has been to disentangle the action of pore edges and pore openings on a porous membrane using a non-soiling, microstructured substrate (Allahyari et al., 2020). Therefore, a non-fouling micropattern has been generated on a silicon dioxide (SiO_2_) substrate, which has a shape, dimension, and discontinuity profile similar to the pore openings of a porous membrane, but with no pore edges. The 3 μm diameter pore size has been selected since it is a frequently employed pore size for membranes in the barrier modeling and it is sufficiently sized to accommodate leukocyte transmigration (Allahyari et al., 2020), whereas it is sufficiently small to impede endothelial cell transmigration in principle (Kukulski et al., 2007; Prabhakarpandian et al., 2013; Takeshita et al., 2014; Casillo et al., 2017; Chung et al., 2018; Salminen et al., 2019). To generate the non-fouling regions in the patterned substrate, poly(L-lysine)-g-poly(ethylene glycol) (PLL-g-PEG) was used, which has been proven to be a proper candidate for producing these types of non-fouling patterns (Michel et al., 2002; Falconnet et al., 2004; Lussi et al., 2006; Marie et al., 2007; Azioune et al., 2010; Vignaud et al., 2012; Rothenberg et al., 2015; Liu and Yang, 2017). The PLL backbone of this polymer facilitates an efficacious adsorption on negatively charged surfaces such as SiO_2_, whereas the polyethylene glycol (PEG) branches impede cell adhesion to the coated substrate (Michel et al., 2002; Falconnet et al., 2004; Azioune et al., 2010). Microcontact stamplography and deep UV laser deposition are widely practiced chemical structuring techniques for the engineering of such a micropatterned surface substrate. However, as with any patterning technique, there are certain constraints, such as poor reproducibility due to stamp wear and poor resolution caused by imperfect polymer removal (Csucs et al., 2003; Azioune et al., 2009, Azioune et al., 2010; Musaev et al., 2011; Ligon et al., 2017; Tewary et al., 2019; Tenje et al., 2020). Photolithography and simple surface adsorption of the PLL-g-PEG polymer could be successfully coupled to fabricate a reproducible pattern with less steps and without the above-mentioned intricacies, thus providing high-resolution micropatterning (Casillo et al., 2017). The resulting microstructured substrate has been applied to examine the fibrillogenesis of fibronectin, migration characteristics, and spreading of endothelial cells. Endothelial cells have similar tendencies in fibrillogenesis of fibronectin and migration speed as previous results on porous substrates, but there were discrepancies in cell spreading and a smaller augmentation in migration speed on these substrates compared with previous findings on micropores. These results lead to the hypothesis that, in addition to the disruptive nature of the open pores, there are other physical drivers that lead to these slight variations in the behavior of the micropores.

#### 3.2.2 Effect of Interstitial Flow on Transmigration

Besides structural cues, also non-structural effects impact the migratory phenotype of cells. For instance, the interstitial flow can be mimicked within these microfluidic chambers that impact cellular mechanical properties and motility, such as the directional migration along the streamline (Polacheck et al., 2017). Apart from this tri-culture assay, solution and surface chemical gradients can be generated within microfluidic chambers (Jeon et al., 2000; Kim et al., 2010). These microfluidic chambers for co-culture of cancer cells with endothelial cells can also be utilized for tumor spheroids (Ko et al., 2019) and therefore represent an intricate experimental platform for analyzing the migratory capacity through extracellular matrix scaffolds and endothelial barriers. There are also organ-on-a-chip models that fully grasp the 3D microenvironment of cancer cells (Glaser et al., 2022). For example, the immune microenvironment of cancer cells has been analyzed employing these organ-on-a-chip technologies (Yoon et al., 2020). Long-term studies are possible without leading to an excess of cell death and less growth factors need to be supplemented. In addition to the aforementioned advantages of 3D cultures, one disadvantage of 3D cultures is that manual handling of the organoids or microtissues and culture medium can be difficult if the microtissues are free-floating, breakable, or when physical access to the tissues is impeded by ambient containers and engineered devices. Some engineered devices ease the management of spheroids by confining them in plugged conical cavities, in perfused compartments in organ-on-chip constructs, or by embedding magnetic nanoparticles (Haisler et al., 2013; Kim et al., 2015).

#### 3.2.3 Impact of Matrix-Mechanics on Transmigration and Cell-Cell Junctions

In a specific type of migration, which is referred to as extravasation (transmigration) of cells through an endothelial cell layer, a stiffer matrix, whatever the mechanism of stiffening, has been found to augment leukocyte-endothelial interactions in the inflammatory pathway. When endothelial cells are grown into a confluent monolayer on stiffer substrates, leukocytes can traverse the monolayer more efficiently than when the monolayer is built on a compliant medium (Huynh et al., 2011; Tao and Sun, 2015). The amplified transmigration has been extensively accounted for by a breakdown in endothelial cell-cell junctions rather than altered expression of inflammatory mediators in endothelial cells; but there is emerging indication that certain cells on stiffer substrates are more susceptible to inflammatory cytokines. Thus, endothelial cells exposed to tumor necrosis factor-α (TNF-α) and thrombin show a stronger rise in tensile forces on stiff matrices in comparison with more compliant matrices (Urbano et al., 2017). Similarly, fibroblasts on stiff substrates are also found to be more responsive to TNF-α (Liu et al., 2015b; Southern et al., 2016), which enhances their spreading and generation of tensile forces. The exact mechanism whereby such elevated sensitivity arises is not understood, although it is probably attributable to an interaction with integrin-related signaling pathways.

However, the stiffness range is the crucial factor. Endothelial cell monolayers constituted more mature cell-cell junctions on soft substrates relative to glass, reflecting enhanced retention of vinculin and F-actin. Endothelial cell monolayers aided transendothelial migration on soft matrices (Mierke et al., 2008). Specifically, the mode of transmigration, such as transmigration between two neighboring cells or transmigration through the cytoplasm of a single cell, is regulated by the stiffness of the substrate. For example, immune cells, comprising peripheral blood lymphocytes and natural killer cells, exhibited a declining incidence of paracellular (between two neighboring cells) transmigration events with reducing substrate stiffness, whereas the incidence of transcellular (through the cytosol of an individual cell) events among peripheral blood lymphocytes enhanced (Onken et al., 2014). In line with this, melanoma cancer cells exhibited elevated transmigration with lower stiffness. Whether the viscoelastic properties of the substrate and of the endothelial cells play an additional regulatory role still needs to be figured out.

When cells, such as cancer cells, transmigrate through the endothelial monolayer, they seem to exert forces on. It is know that force application to the endothelial adhesion molecule PECAM activates GEF-H1 and LARG (Collins et al., 2012), whereas force application to endothelial ICAM-1 activates only LARG (Lessey-Morillon et al., 2014). Additionally, tension on JAM-A in endothelial cells leads to the activation of GEF-H1 and p115RhoGEF, whereas LARG is not activated (Scott et al., 2016). Consequently, when force is applied to cells various cell adhesion molecules govern the activation of one or two of a group of GEFs, encompassing GEF-H1, LARG and p115RhoGEF. It remains to be determined whether the variations are attributable to distinct adhesion molecule composites or to variations across cell types and traction modes. However, it has been shown that the mechanical properties of endothelial cells, such as their stiffness and fluidity are altered (Burridge et al., 2019).

The formation and sustenance of the majority, perhaps all, of animal tissues and organs is governed to a certain degree through the action of mechanics (Engler et al., 2006; Felsenthal and Zelzer, 2017; Urner et al., 2018). The focal adhesion protein talin and integrins perform pivotal tasks in the perception of and reaction to mechanical forces. Specifically, cells feel the rigidity of the extracellular matrix and the tissue strain conveyed from the extracellular matrix through integrins (Elosegui-Artola et al., 2018; Sun et al., 2016). Both of these cases increase the tension on the extracellular matrix integrin-cytoskeleton connection, resulting in stronger integrin bundling and increased signaling performance. Comprehension of these events is imperfect, but the key general principle is that both strain and rigidity alter the amount of stress present within the integrin-cytoskeleton junction, thereby changing the conformations and interactions of the proteins concerned. The core of mechanotransduction is how mechanical force is translated into a biochemical alteration, for example, the level of an effector at the adhesion site or the post-translational alteration of proteins.

### 3.3 Molecular Mechanosensory Interaction of Cells and Their Matrix Environment

It is well acknowledged that 2D cultures suffer from multiple constraints, such as disruption of interactions between the cellular and extracellular surroundings, alterations in cell morphology, polarity, and division pattern (Weaver et al., 2002; Mseka et al., 2007; Kapałczyńska et al., 2016). All of these alterations seem to impact the viscoelastic behavior of cells, spheroids and organoids. These drawbacks led to the emergence of models that can better mimic *in vivo* settings. 3D culture is this kind of technique. Within 3D culture setting, the storage and liberation of growth factors or cell surface receptor ligands or matrix-degrading enzymes and their regulatory molecules is possible compared to 2D culture systems. In addition, the diffusion of substances through a 3D matrix environment seems to be different and is likely to be hindered. The geometry and topology of the 3D matrix environment farther alters the cellular phenotype and consequently cell function. In agreement with this, the migration of cells can be impaired by a dense 3D matrix scaffold that cannot be broken down enzymatically by cellular substances or mechanically through bond rupture by cellular forces. Therefore, choosing the most adequate cell culture techniques in the field of cancer research may permit a better comprehension of cancer biology and thus optimize radio- and chemotherapy or discover innovative therapeutic options (Aggarwal et al., 2009). 3D culture tests represent a major advantage as they can preserve organotypic functionality as extensively as feasible. A simple approach is to employ completely differentiated cells that are directly prepared from living tissues, since they are considered to be in a native phenotype (Zuppinger, 2019).

In the historical context, 3D cultures have been first utilized systematically for pharmaceutical testing in cancer biology, explained in a part due to the presence of cellular aggregates containing a hypoxic core that share many resemblances with avascular solid cancers (Inch et al., 1970). It has been consistently shown that solely 3D technologies using co-cultures are capable of mimicking pivotal features of phenotypic and cellular heterogeneity, and microenvironmental facets of cancer growth (Thoma et al., 2014).

#### 3.3.1 2D vs 3D Culture Conditions Affect the Cellular Mechano-Phenotype

The 2D vs 3D culture conditions have been seen to be different in cardiomyocytes. The mechanical properties of cardiomyocytes have been identified to be heavily reliant on the composition and organization of the matrix, and time in cell culture. Cells appeared to stiffen and relax less in the first 3–5 days in cultivation prior to achieving a plateau in their mechanical characteristics. After the fifth day, cells on aligned matrices tended to be stiffer compared with cells on unaligned matrices, and cells on fibronectin matrices seemed stiffer compared with cells on collagen matrices (Deitch et al., 2012). In contrast, cardiomyocytes are subjected to a plethora of biochemical, mechanical, electrical, and other types of irritants that result in adequate reactions and finely tuned alterations in gene expression (Zuppinger, 2019). In addition, they also sense the shear stress generate through the blood flow. All of which is also applicable to other cells types, such as cancer cells or endothelial cells.

The overwhelming number of biological tissues have the capacity to distort and adjust to their new environment when faced with defiance from physical forces. Cells change their perceptual response depending on the microenvironment, as the cellular response to micrometer and submicrometer scale columns/pillars is fundamentally different (Ghassemi et al., 2012). In addition, depletion of rigidity sensing modules can elevate the growth of cancer cells (Yang et al., 2020). Thus, the precise interplay between intracellular elements of cells and environmental cues is critical for regulating cellular mechanotransduction that guides cell growth and thereby prevents excessive growth of cancer cells (Yang et al., 2020). Cells clad on 2D or 3D microstructured surfaces are prone to match their form to that imprinted on the pre-patterned surface structure (Théry, 2010). The question still remains unanswered as to whether the surface disturbance caused by pore opening is the only factor contributing to the alteration of the migration behavior of cells on a porous membrane, or whether the pore edges should also be considered as a decisive factor in the regulation of cell migration and spreading on a porous membrane. Pore edges, which can provide increased vertical contact area for cell adhesion and migration, have not been previously investigated as a separate contributing factor in studies involving porous membranes. Additionally, there is a growing interest in utilizing microposts to mimic cellular microenvironments in terms of mechanical cues. These studies demonstrate how flexibility of microposts can regulate cell spreading and migratory behavior of cells through modulating substrate rigidity (Allahyari et al., 2020; Yang et al., 2017; Zhong et al., 2020). However, similar to the role of pores in porous membranes, pillar walls can be considered as potential gripping points for cells, and this topological aspect of microposts can influence cell migration and spreading in addition to the substrate rigidity effect (Bettinger et al., 2009; Irvine et al., 2001, Irvine et al., 2002). Therefore, it is important to study pore edges or pillar walls as a contributing factor for the aforementioned changes in cellular behavior, particularly on porous membranes which are employed in an ever-increasing number of experimental examinations.

#### 3.3.2 Structural Cues Impact Cell Migration

Moreover, during migration *in vivo*, cells generally prefer to squeeze through exceptionally narrow extracellular gaps, and in an effort to accommodate these intricate geometries, they extensively misshape (Théry, 2010; Lange and Fabry, 2013; Calero-Cuenca et al., 2018). Although the extent of distortion exhibited by cells or tissues can differ according to the time or nature of the tissue sampled, the majority of biological tissues act as nonlinear viscoelastic materials when subjected to physical stresses. A rather simplified meaning of viscoelasticity of biological matter is that the identical tissue behaves viscously and elastically when mechanically strained (Burstein and Frankel, 1968; Kucharová et al., 2007; Pajic-Lijakovic and Milivojevic, 2017). In general, biological matter, such as living tissues, have not commonly been investigated as non-linear viscoelastic materials. As mentioned before, pioneering work in mechanobiology treat biological tissues as purely and simplistic linear elastic materials, whereby Hookean behaviors is hypothesized when it is subject to mechanical deformation. As emphasized in this review, this view has changed, since biological materials have solely been seen to explore elastic characteristics when a temporal element is not included. However, when addressing time as an independent element in experimental setups, the curves displaying the reaction of biological matter to external mechanical deformation would become non-linear characteristics, when related to a control curve representing a “Hookean material” (Burstein and Frankel, 1968). This non-linear mechanical reaction of biological systems has lately been reexamined and proven by utilizing purified and *in vitro* reconstituted cellular compounds. It assists in elucidating why several biological materials, including cells, undergo stiffening when stretched to prevent large deformations and preserve the intactness of the tissue (Goldenberg and Goldhirsch, 2005; Storm et al., 2005; Pogoda et al., 2014; van Helvert and Friedl, 2016).

Therefore, a comprehensive framework has been established in which the response of biological materials to deformation is divided into, firstly, short-term linear-elastic behavior, which is an instantaneous, almost time-independent response, and secondly, time-dependent viscoelastic response. The latter can be considered at different time scales and structural levels, such as short-term subcellular, mid-term cellular, and long-term supracellular regimes (Thoumine and Ott, 1997; Bausch et al., 1999; Vicsek and Zafeiris, 2012; Pajic-Lijakovic and Milivojevic, 2017). Although the short- and medium-term regimes are in fact well recorded, the observation of a long-term supracellular viscoelastic regime is relatively less examined (Pajic-Lijakovic and Milivojevic, 2017). While individual cells are able to quickly reorganize cytoskeletal elements and react to stress (Mitrossilis et al., 2010), clusters of cells may take longer when reacting in a coordinated manner to environmental stress. Probably owing to the time delay caused by the demand for higher coordination of these cytoskeletal redistributions (Pajic-Lijakovic and Milivojevic, 2017). The advantage of 2D culture conditions are the high throughput and the capability of addressing intracellular structures impacting mechanical cues in more detail, due to the less working distance required and non-disturbed imaging quality through matrix scaffolds surrounding the cells. A major disadvantage is that the viscoelasticity can be altered through the viscoelasticity of the 3D matrix environmental scaffold that acts on cellular mechanical characteristics.

The cross-talk between human cancer cells, such as breast cancer cells, human endothelial cells and human tissue parenchyma, such as osteo-cell-conditioned extracellular matrix to mimic bone tissue, can be deciphered using 3D microfluidic tri-culture systems (Bersini et al., 2014). It has turned out to be useful in determining whether breast cancer cells migrate more efficiently into osteo-cell-conditioned matrices compared to non-conditioned matrices. These findings have enlightened the process of cancer cell dissemination to targeted tissues or organs.

In the following section, cellular components involved in regulating the viscoelastic response of cells and tissues are introduced together with their reaction to deformation and specific selected mechanisms that govern the dynamical processes of these cellular elements.

## 4 Regulation of Cellular Constituents, Focal Adhesion, Cytoskeletal and Nuclear Component Assembly, Restructuring and Turn-Over

The molecular mechanotransduction is founded on a linear propagation of steps. First, forces acting on cells and imposed by cells on the extracellular environment cause stresses and deformations that are perceived by a panel of specialized molecules termed mechanosensors (Box 1). These mechanosensors experience a force-dependent conformational alteration that modifies the biochemical functionality of the protein. Forces from the cellular environment are usually encountered primarily at the surface of the cell, where the force-generating cytoskeleton can also apply stresses as it comes into contact with various mechanical conditions. The adhesion complexes that connect cells to ambient tissues by focal adhesions and to other cells by adherens junctions have consequently been found to be key hubs in the transmission of forces (Leckband and de Rooij, 2014; Seetharaman et al., 2021; Sun et al., 2016). There are, however, a much wider spectrum of mechanosensors inherent in cells, comprising several structurally diverse families of force-sensitive ion channels (Kefauver et al., 2020) and receptors for biochemical ligands that react in a direct manner to force, such as notch (Stassen et al., 2020) and plexin (Mehta et al., 2020). In addition, forces at the cell periphery are propagated through the cytoskeleton to other cellular locations such as the nucleus (Cho et al., 2017), which also harbor mechanosensitive compounds and enable cellular reaction to external and intrinsic forces.

Mechanosensors operate using a number of common mechanisms through the force-induced conformational alterations that impact either molecular perceptual interactions or the activity of the protein. Forces directly amplify the protein-protein interaction of mechanosensors in that they enhance the lifetime of the bond (catch bond), in contrast to the majority of protein-protein interactions in which the lifetime reduces in response to the force (slip bond) (Zhu et al., 2019). In addition, forces are able to mutilate the form of interactions through protein unfolding or unmasking, which results in either the exposure of cryptic binding sites (del Rio et al., 2009; Yonemura et al., 2010) or the breakdown of binding motifs (Ehrlicher et al., 2011). The character of the cryptic site differs among a variety of mechanosensors, and forces involved can uncover proteolytic sites (Gordon et al., 2015; Mack et al., 2017) or motifs for the purpose of posttranslational processing (Sawada et al., 2006). Multiple membrane-associated mechanosensors are adjusted in response to force-induced alterations in membrane tension, for example, through regulating the gate operation of mechanosensitive ion channels (Saotome et al., 2018). In addition, cytoskeletal forces are also capable of stabilizing certain structural conformations of mechanosensors including integrins (Li and Springer, 2018). Mechanosensors frequently constitute broader multimolecular complexes with assemblies of different mechanosensors governed by various mechanisms, prototypical instances of these being focal adhesions and adherens junctions.

Mechanosensors do not function like ordinary on-off relays, instead their reaction involves different characteristics of the forces. Forces themselves can operate on distinct sections of the cell, and can have varying magnitudes, directions, and temporal characteristics, each resulting in a specific unique reaction and varying biological results. The intrinsic mechanisms of force transmission in an individual mechanosensor, and its organization inside the cell, dictate the capacity to discriminate between these various kinds of parameters. All of which is explained in more detail in the following sections.

What is the effect of the well-investigated plasma membrane tension? Membrane tension emerged as a crucial mechanical factor for cell locomotion, shape modification, and volume adjustment (Tao and Sun, 2015). An elevation in membrane tension is observed to augment the crawling speed of osmotically shocked *Caenorhabditis elegans* spermatocytes by restricting extraneous protrusions (Batchelder et al., 2011) and works as a widespread inhibitor of actin assembly, thereby aiding neutrophils to preserve polarity and restricting the pseudopod count to one (Houk et al., 2012). Membrane tension levels have also been demonstrated to direct the positioning of focal adhesions at the leading edge of mouse embryonic fibroblasts through inducing the buckling of actin filaments without the function of myosin II (Pontes et al., 2017). In addition, it can restrict actin assembly at the front edge of creeping neutrophils via a negative feedback circuit that engages phospholipase D2 (PLD2) and mammalian target of rapamycin complex 2 (mTORC2) (Diz-Muñoz et al., 2016). Each of these investigations has been conducted on stiff substrates. Thus, how would a softer substrate impact the membrane tension? In a recent investigation, membrane tension of hepatocellular carcinoma cells on soft hyaluronic acid and stiff polyacrylamide substrates has been assessed through pulling membrane tapes with optical tweezers (Mandal et al., 2019). The membrane tensions measured on soft 300 Pa hyaluronic acid media are somewhat equivalent to those measured on 30 kPa polyacrylamide media, and the magnitude of the measured membrane tension does not appear to correspond to the traction forces imposed by the cells on the media. This indicates that the forces generated by cells on their extracellular matrix are not substantially modifying the membrane tension (Mandal et al., 2019).

### 4.1 Viscoelasticity of Cellular Constituents

It is not quite clear whether cytoskeletal constituents of cells possess and inherent viscoelasticity or contribute in structural terms to the global viscoelasticity of cells. There are some features of cellular components that can contribute to mechanical characteristics of cells, including viscoelasticity. In order to withstand or deform without impacting the integrity of a cell or cluster, subcellular constituents are dynamically realigned throughout cell migration. In other words, to react to external mechanical cues, cells simply need to be able to detect them. The mechanism that enables cells to sense and react to physical stress from their microenvironment comprises the sensing of mechanical cues, termed mechanosensing, and their conversion into a biochemical reaction, referred to as mechanotransduction (Charras and Yap, 2018). The mechanotransduction process enables cells to be potentially able to react to mechanical challenges through adapting not only their stiffness, but also their viscoelastic response. In order to understand the aforementioned hypothesis a brief excurse in the canonical process of mechanosensing is provided in the following.

The cellular answer to physical signals provoked by mechanical stress involves a short- or medium-term adjustment of cell shape via cytoskeletal reassortment, usually entailing post-translational modification of scaffoldings, adhesion, polarity, and contractility associated proteins (Ren et al., 2009; Moore et al., 2010; Pandya et al., 2017). Beyond this, a long-term responsiveness can also be witnessed, fueled by changes in gene expression profiles that are subsequently reflected back in cellular performance, which is far more less explored compared to the canonical process.

In adherent cells, the detection and signal transduction of mechanical cues are intricate processes, and herein are briefly concerned with a “canonical” and somewhat simplified sensing mechanism (Jansen et al., 2017; Gauthier and Roca-Cusachs, 2018; Yap et al., 2018). Among the first proposed structures participating in mechanosensing are the focal adhesion sites, where integrin adhesion receptors attach the cell tethered to the extracellular matrix (Jansen et al., 2017; Gauthier and Roca-Cusachs, 2018). This engagement elicits the assembly of a set of proteins into focal adhesions that couple this integrin receptor to the cytoskeleton (Sawada et al., 2006; Moore et al., 2010; Schiller and Fässler, 2013; Fedorchak et al., 2014; Paluch et al., 2015; Petridou et al., 2017). Finally, the force encountered at the focal adhesions is transmitted into the cytoskeleton through a protein complex composed in parts of vinculin and talin-driven force transfers (Grashoff et al., 2010; A.; Kumar et al., 2016; Sawada et al., 2006; Yao et al., 2016). The cytoskeleton then back-couples into the focal adhesion to enhance the dynamic nature of this structure through myosin II-facilitated contractility, leading to substrate distortion in cells migrating into compliant surfaces and cell distortion and migration in cells migrating over stiffer substrates. When a cell is mechanically deformed, the cytoskeleton will transduce these mechanical loads from the microenvironment via filament linkages into the nucleus to alter the expression of genes and consequently biochemical signaling (Paul et al., 2008; Fouchard et al., 2011; Paluch et al., 2015). The recognition and translation of mechanical environmental stimuli into a cellular and molecular response is not only driven by linking focal adhesions and the cytoskeleton in a bidirectional manner, mechanosensing also entails the activation of mechanosensitive ion channels, such as Piezo1 (Gudipaty et al., 2017; Hung et al., 2016; Wu et al., 2017), or mechanically reactive nuclear pores and the subsequent activation of targeted transcription factors (Elosegui-Artola et al., 2017; Stanley et al., 2017; Kirby and Lammerding, 2018; Otsuka and Ellenberg, 2018).

The mechanical response of talin is pivotal to the intricate regulatory mechanisms that manage force transfer between the actin cytoskeleton and the extracellular matrix. The principal mechanism underlying the force transmission paradigm observed between F-actin and integrins is “focal adhesion coupling (referred to as clutch),” which characterizes reciprocal interactions between relatively static, ligand-bound integrins and centripetally flowing F-actin in the vicinity of cell borders (Elosegui-Artola et al., 2018). The linkages that convey force in this environment are highly dynamic, exhibiting rapid on and off rates. The stiffness of the extracellular matrix changes the rate of loading applied through these linkages, which modifies the internal kinetics. The importance of stiffer media is that they increase the traction force and thereby provide stability to the adhesions (Elosegui-Artola et al., 2018), along with the force generated through the substrate stretching (Sun et al., 2016). While these effects have been inferred to the focal adhesion clutch model of dynamic force transmission (Elosegui-Artola et al., 2018), emerging evidence has questioned this modeling paradigm and revealed a more intricate mechanism. Tension analysis over talin combined with actin dynamics identified three separate mechanisms of force transfer, but only one of them is dynamic (Driscoll et al., 2020). In newly created cell adhesions close to the cell edge, force transmission occurs through fast-flowing actin powered by rapid polymerization at the edge, as outlined in the clutch model. Nevertheless, when vinculin is being recruited and actin velocity drops, force transduction switches to a flow-independent transmission powered by myosin contraction. While this is coherent with the stability of vinculin-F-actin linkages under force, which are referred to as catch-bond properties (Huang et al., 2017), it is not in agreement with the short-lived linkages of the clutch model. Therefore, vinculin has more of a part to play in locking the moving actin filaments and forming stable junctions than in providing dynamic force transduction. What is important to note is that the homeostatic relationship among these mechanisms is governed by substrate rigidity, so that dynamic force transduction is actually more dominant on soft substrates, while flow-independent force transduction is prevalent on stiff substrates (Driscoll et al., 2016). A pivotal perspective involves the feedback between the forces and the sensing complex. Cells located on stiff interfaces or exposed to strain increase their adhesions and enhance the contractile force exerted by the cell, thereby altering its mechanosensation. For instance, highly contractile cells need fairly stiff supports to completely spread, whereas less contractile cells can spread fully on softer surfaces (Discher, 2005). In other words, cells can accommodate themselves to surroundings with extremely diverse mechanical characteristics.

While these are canonical mechanosensing mechanisms, the exact mechanism through which cells perceive and react to mechanical cues as they migrate as a collective is only beginning to be explored. *In vivo*, the fibronectin-based extracellular matrix of Xenopus, chick, or zebrafish embryos (Bajanca et al., 2019) is around 10 μm thick, and recent *in vitro* strategies indicate that single cells can scan their substrate to a depth of around 15 μm (Buxboim et al., 2010), in this respect, a mechanism similar to the aforementioned one suits ideally to the scanning of the extracellular matrix by individually migrating cells. Nevertheless, it is suggested that collectively migrating cells apply 10 times larger forces to the substrate. Therefore, the interval at which a migrating cluster of adherent cells perceives physical variations to its microenvironment is actually deeper than that recorded for individual cells (Tusan et al., 2017). But how this enhanced sensitivity is accomplished by the group of cells, or even whether this is the situation *in vivo*, remains to be further examined.

Since, poroelastic effects (see 8.1 for details) overlap with other mechanical responses of tissues and extracellular matrices, involving nonlinear elasticity, viscoelasticity, and viscoplasticity (Chaudhuri et al., 2020), analogous mechanisms pertain to the viscoelasticity of the cytoskeleton of cells (Mitchison et al., 2008; Hu et al., 2017; Mollaeian et al., 2018). However, there are two major dissimilarities between poroelastic effects of living cells and extracellular matrices. Firstly, the comparatively impermeable cell membrane prevents or delays poroelastic effects because of overall cell deformation, however, local contraction of the cytoskeleton can produce intracellular poroelastic actions and transient compressive gradients that are sustained for periods of biological concern (Moeendarbary et al., 2013). Secondly, the difference lies in the fact that covalent bonds between the filaments of the cytoskeleton are extremely few or absent. In this regard, moreover, motor proteins exert incidental, non-thermal forces on the filaments of the cytoskeleton (Guo et al., 2014), moving them faster than they would if driven by thermal motion only, with the net effect that the active cytoskeleton is more extensively fluidized than one lacking motors (Humphrey et al., 2002). Cellular viscoelasticity behavior can even be evident at the tissue level. For instance, rigor mortis, the stiffening and solidification of muscle after death, is due partially to the fact that the connections between actin protein filaments and myosin motor proteins in muscle fibers grow in both number and permanence as the living muscle hydrolyzes ATP, thereby allowing the actin-myosin connections to build and become dissociated quickly.

Nonlinear elasticity is also evident in cytoskeletal filament networks, such as actin, vimentin, and neurofilaments, however, the source of nonlinear elasticity in these structures may reflect a stronger input from entropic elasticity due to the semiflexible character of the filaments (Storm et al., 2005). In the following a possible role of these biological intracellular cytoskeletal, compartmental or nuclear structures may play a role in the viscoelastic characteristic of cells.

### 4.2 Viscoelasticity of Focal Adhesions

Similar to adherens junctions, focal adhesions comprise a number of mechanosensor proteins employing various mechanotransduction mechanisms. The conversion of mechanical impulses into biological events and their subsequent reaction can be broadly classified into two distinct phenomena. The first phenomenon is the active or passive reaction of cells to externally exerted forces, such as when fluid shear stresses act on the endothelium as a result of blood flow across the vasculature, or when gravity acts on joints, bone, or adipose tissue. These types of excitements provide the foundation of Wolff’s law for bone remodeling, the connection between modified blood flow and the onset of atherosclerosis, or the stress of expanding cancers (Yusko and Asbury, 2014). The second phenomenon is the cell and tissue reactions resulting from the forces produced directly by the cell, which are resisted due to the viscoelastic or active characteristics of the extracellular matrix or the ambient cells.

The transduction of biochemical cues frequently involves allosteric alterations in protein conformation or phosphorus regulation. Fluctuations in phosphorylation levels can directly affect the activity of a binding moiety or an active site within an enzyme, and they can cause conformational rearrangements in a manner akin to allosteric effects. Since mechanical stresses on a protein have also been found to modify its conformation, evolution efforts not surprisingly have yielded molecules that leverage force-dependent conformational modifications to convey and transduce mechanical inputs (Ingber, 1997). The model describing the structural basis for mechanotransduction is termed tensegrity model.

Cellular reactions to mechanical stimuli including flow, stiffness or viscoelasticity of the extracellular matrix, and tissue extension are related to the magnitude of the forces connected to these stimuli. The range of force magnitudes perceived by the cells and the susceptibility of the various mechanosensors in this range dictate how the cells react to the mechanical stimuli. Even though the molecular underlying mechanisms of susceptibility to force magnitudes have not been fully elucidated, multiple mechanisms whereby cells can derive this kind of information have been identified. A molecular concept for sensitivity to force magnitudes is that mechanosensors exhibit a critical threshold force for activation, such as the force necessary to uncover cryptic binding sites or the force regime where capture bonds are established. Moreover, this sensitivity can be finely tailored through the presence of stable intermediate modes for the force-induced conformations of specific mechanosensors. For example, single-molecule force spectroscopy of catch bonds identified three main modes (weakly, intermediately, and strongly bound) at a variety of force levels for fibronectin-integrin (Kong et al., 2009), vinculin-F-actin (Huang et al., 2017), and VWF-GPIb (Ju et al., 2013).

RhoA has been determined to fulfill a function in mechanotransduction that depends on the tension exerted on fibronectin-coated beads attached to the surface of the cell membrane (Matthews et al., 2006) and additionally tension on integrins has been seen to activate RhoA (X.-H. Zhao et al., 2007). Activation involves either activation of a GEF or engagement of a GAP. GEF-H1 and LARG have been revealed to be active in reaction to tension on integrins (Guilluy et al., 2011). Examination of the signaling pathways upstream of these GEFs indicated that LARG is activated through phosphorylation of the Src family kinase Fyn, whereas GEF-H1 is activated through the MEK/ERK pathway after focal adhesion kinase (FAK) activation (Guilluy et al., 2014).

Activation of GEFs that occurs in reaction to mechanical force on fibronectin-coated beads is a downstream reaction to stress-induced activation of kinases, specifically members of the Src kinase family and/or FAK. The activation of FAK takes place after integrin entanglement and establishment of focal adhesions (Burridge et al., 1992). Inhibition of tension on integrins by hampering myosin activity using blebbistatin diminishes activation of FAK (Pasapera et al., 2010). It has been demonstrated that tension on integrins facilitates integrin binding to the synergy site of fibronectin in addition to the RGD binding site in a manner that stimulates activation of FAK (Friedland et al., 2009). In agreement with this result, others have shown that tension on fibronectin uncovers the synergy binding site causing the α5β1 integrin to attach to it and also to the RGD binding site (Seong et al., 2013). It can be anticipated that FAK is clustered as a consequence of full integrin-fibronectin binding and that clustered FAK is trans-phosphorylated, thereby causing FAK activation. Consequently, tension can be applied to FAK to free it from the auto-inhibited conformational state (Zhou et al., 2015). In line with this, other focal adhesion proteins, including talin, are stretched due to mechanical tension applied to integrin-based adhesions (del Rio et al., 2009). The question is whether these other deformations proceed in concert with elongation and activation of proteins, including talin, vinculin, Src and FAK, or whether they follow one after the other.

Focal adhesions can be altered based on the turnover of focal adhesion proteins and the stretching of focal adhesion proteins that act as mechanosensors. There are several mechanisms through which focal adhesion proteins can be altered by a stretching force: the cryptic binding site, such as talin, and the cryptic phosphorylation motif, such as p130Cas. When talin connects integrins and actin, it transfers both cell-generated contractile forces and forces originating from externally applied loads across these constituents. Forces sampled through talin span from just a few to over 11 piconewtons (Austen et al., 2015; Driscoll et al., 2020; Kumar et al., 2016). Talin reactions to forces share four characteristics with key implications. Firstly, forces tend to keep the elongated conformation of talin stable (Khan and Goult, 2019) because the head and tail are kept separated through tension, thereby constraining the autoinhibition conveyed through head-tail interactions. Secondly, the coupling of talin with actin and with integrin displays catch-bond characteristics, which means that the binding strength increased in response to moderate forces (Owen et al., 2020) that additionally stabilizes the activated, committed conformation. Third, the force unwrinkles the helical bundles of the talin rod domain; which simultaneously breaks the bonds of proteins tethering the folded state and uncovers binding sites for others. Fourthly, the unfolding of the talin rod domain presents hysteresis, which means that the force necessary for unfolding is stronger than the force needed to refold it. When a rod domain unfolds due to a force of 10 pN, it fails to refold instantaneously when the force is less than 10 pN. Refolding demands much lower tension of approximately 1–3 pN (Yao et al., 2016). In this way, the basic physiological forces of around 5 pN on talin inside focal adhesions (Kumar et al., 2016) tend to keep the patterns of folded and unfolded talin rod domains stable (Yao et al., 2016). Consequently, these characteristics confers a mechanical memory to the talin (Goult, 2021).

The structure–function analysis of talin lead to a model for mechanotransduction. High affinity sites for vinculin had been charted on the talin rod, however succeeding structures of the respective domains exhibited that these sites remained masked (Papagrigoriou et al., 2004). This led to the general idea that mechanical unfolding of the talin domain is necessary for vinculin binding, which has later been corroborated using single-molecule biophysics (del Rio et al., 2009; Yao et al., 2015). This original scheme has been further extended when it was found that the Rap1 effector RIAM attaches to the folded R3 domain. In this case, the force dislodges RIAM and thereby enlists vinculin, which constitutes a smart mechanical toggle that alters the affinity for the two ligands (Goult et al., 2013; Lee et al., 2013; Vigouroux et al., 2020; Wang et al., 2019). The existence of 13 such domains across the Talin rod, which unfold at distinct forces, is fascinating and raises options for highly intricate force sensitivity, including time-dependent effects (Yao et al., 2016).

Force-independent cross-talk between talin and vinculin has also been noted (Atherton et al., 2020; Austen et al., 2015; Han et al., 2021; Kelley et al., 2020), even though such cross-talk involves the gradual elimination of autoinhibition of the two proteins. This fits with the hypothesis that these proteins need to interact initially in a non-mechanical fashion to establish the bonds for force transfer. When force is exerted, unfolding of the talin helix bundle domains uncovers vinculin binding sites, which bind and further act to stabilize the active conformations of talin and vinculin (Wang et al., 2021; Yao et al., 2015). Therefore, the force is also a stabilizer for the open state of talin and vinculin, which is retained even after a severe decrease in stress (Yao et al., 2015). The binding of vinculin also provides linkages to F-actin, which may increase the force on talin and promote greater force transfer (Kumar et al., 2016). Higher tension levels subsequently further enhance the forces on neighboring areas. Thus, there are several molecular pathways that, once talin is open and in tension, it sustains talin domains in an open conformation of high tension. Oppositely, ligands attached to folded talin-helix bundles act to stabilize this conformation and enhance the force necessary for opening, which depends on the expression and affinity of the ligands. Thereby, the closed configurations are made subject to a positive feedback loop. These mechanisms stabilizing open or closed conditions constitute a type of molecular memory. Consequently, these mechanisms significantly prolong the open and closed condition lifetimes for each molecule (Khan and Goult, 2019; Wang et al., 2019; Wang et al., 2021), an essential type of mechanosensitivity.

What role plays the interaction of DLC1 and talin? The tumor suppressor deleted in liver cancer 1 (DLC1) seems to be important due to its recruitment to focal adhesions (Kawai et al., 2004). In focal adhesion, DLC1 can interact with the mechanosensitive protein focal adhesion protein talin. Specifically, DLC1 is found to bind to the R8 domain of talin, thereby DLC1 is clearly localized in focal adhesions and activated, whereby it causes a reduction in the active RhoA level. Nevertheless, when tension is enough to stretch talin and open the R8 domain, DLC1 is liberated in a conformationally constrained manner and accounts for enhanced RhoA activity (Haining et al., 2018). The liberation and subsequent deactivation of DLC1 from stretched talin suggests an additional pathway by which mechanical tension imposed on integrin adhesions is capable of augmenting RhoA activity. Therefore, a potential negative feedback pathway may also be responsible, which could be relevant in restricting the focal adhesion size. It recognized that mechanical tension encourages the growth of focal adhesions via a RhoA-dependent pathway (Riveline et al., 2001). Tension at focal adhesions stretches their constituents such as talin, thereby enlisting additional binding partners (del Rio et al., 2009). Large adhesions, nonetheless, have been found to produce lower traction than small adhesions (Beningo et al., 2000), and FRET-based stress sensors have demonstrated that lower tension is transferred to constituents within large focal adhesions compared to small adhesions (Austen et al., 2015; Grashoff et al., 2010; Kumar et al., 2016). A negative feedback circuit needs to be in place to avoid additional growth of the focal adhesions as a response to the growing tension.

### 4.3 Cytoskeletal Viscoelasticity

**FIGURE 2:**
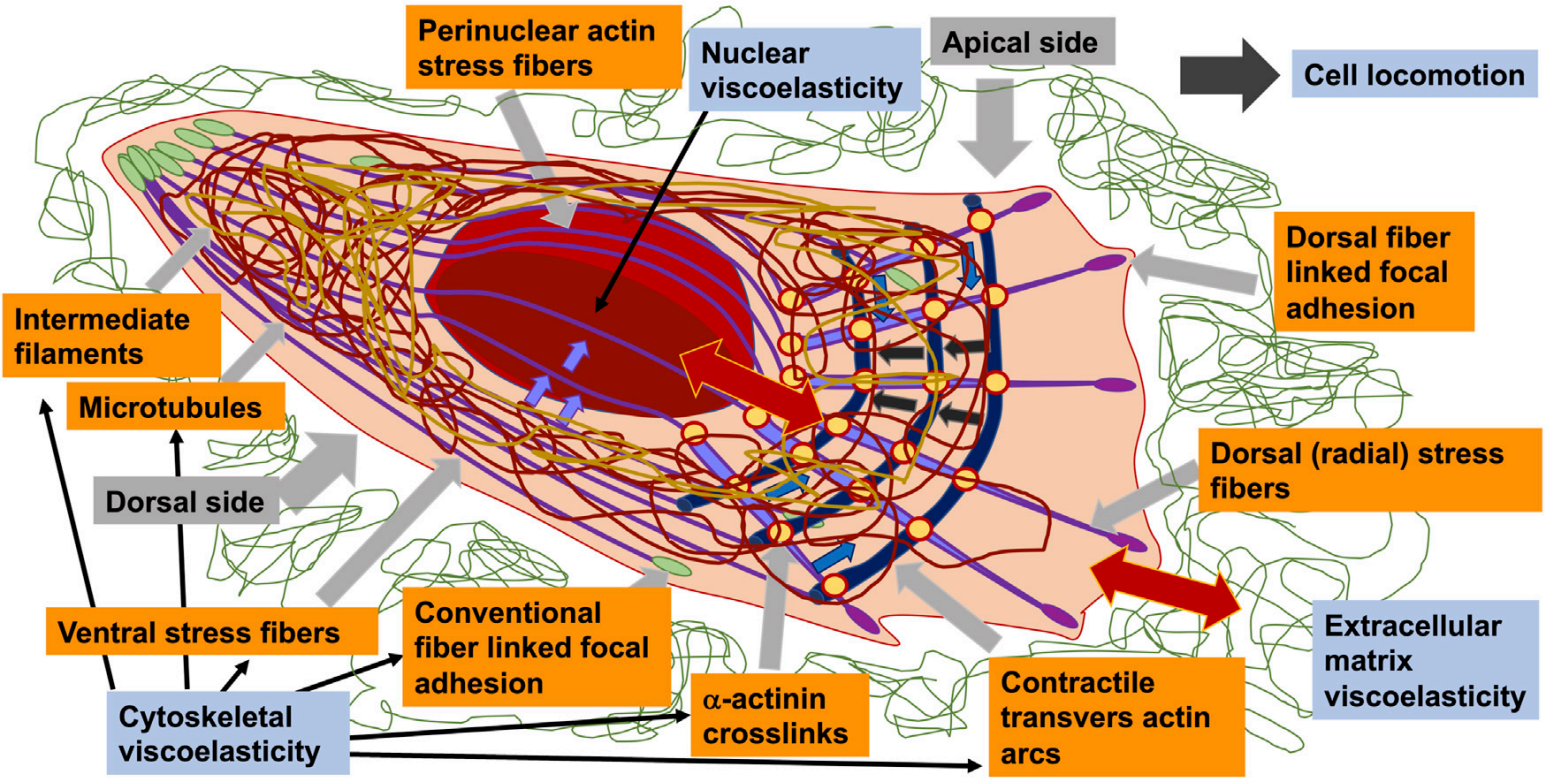
Possible viscoelastic features of cellular constituents, such as nucleus, cytoskeleton that interact through mechanotransduction processes with the viscoelastic characteristics of their natural environments, such as extracellular matrix scaffolds of connective tissues.

The cytoskeleton functions not merely as an integral regulator of molecular circuitry, but also as a mesoscale mechanosensor with its inherent level of regulation and dynamics. Specifically, the cytoskeleton has a critical involvement in the majority of suggested mechanisms for conveying the perception and transmission of mechanical signals from the microenvironment, and its constituents experience large alterations during cellular deformation in response to mechanical stress (Wen and Janmey, 2011). The basic elements of the cytoskeleton are biological polymers known as intermediate filaments, actin filaments and microtubules (Fletcher and Mullins, 2010; Stamenović and Wang, 2000). An equilibrium of the polymerization/depolymerization fraction and the extent of crosslinking of these structures governs whether a cell distorts or withstands deformation when exposed to mechanical stress (Figure 2). The degree of build-up and breakdown of these elements is regulated by a number of molecules. For instance, actin nucleation factors induce and elongate polymer filaments, capping factors cease the growth of filaments, and there are depolymerizing and detaching factors that break down these filaments (Stamenović and Wang, 2000; Wear et al., 2000; Carlsson, 2010; Reymann et al., 2012; Salbreux et al., 2012; Dang et al., 2013; Krause and Gautreau, 2014; Veltman, 2014).

The mechanical performance and characteristics of these three filaments have been characterized in various systems. Microtubules emerged as the more rigid part of the cytoskeleton, with a less rigid meshwork of actin filaments and a more soft meshwork of intermediate filaments (Fletcher and Mullins, 2010; Hawkins et al., 2010; Stricker et al., 2010; Wen and Janmey, 2011; Salbreux et al., 2012; Lopez and Valentine, 2015; Charrier and Janmey, 2016). Mechanical stress on the cytoskeletal reticulum has also been assessed in epithelial cells through flow analysis, and mechanisms monitoring the stiffening of the cytoskeleton have been put forward (Helmke et al., 2003; Žagar et al., 2015). Apart from the polymerization and passive mechanical characteristics of its constituents specifying the mechanical condition of the cytoskeleton, it has also been theorized that crosslinking factors have a part to play in defining the architecture of the cytoskeleton’s filaments and, as a result, their elastic condition (Brighenti and Vernerey, 2017; Harris et al., 2012; Kirschner, 1986; Lieleg et al., 2009). The activity of molecular motors for instance myosin II in conjunction with cross-links is also connected to the viscoelastic characteristics of the cytoskeleton (Paul et al., 2008; Murrell et al., 2015). The effective coordination between filaments, motors and cross-links is mechanically excited. By way of illustration, the engagement of myosin with actin fibers proceeds in a force-dependent fashion, the same as the contractile reaction of actomyosin to extracellular stiffness, as evidenced through cell elongation and real-time imaging (Mitrossilis et al., 2010; Fouchard et al., 2011). Actin assembly assays reveal that force-generating actin webs accommodate to external mechanical forces and that force reconnection between intracellular and extracellular forces improves the tightness and mechanical efficacy of newly ramified actin webs (Bieling et al., 2016).

What about the viscoelastic properties of cytoskeletal filaments? Actin networks cross-linked by filamin have been found to display dramatic macroscopic stress-hardening response. By imposing a prestress, the nonlinear stiffness is adjustable over multiple orders of magnitude, whereas the linear elasticity of the reticulation stays at a moderate level (Gardel et al., 2004). In contrast, for instance, to cross-linked actin/heavy meromyosin webs, in which the inclusion of heavy meromyosin leads to a very marked rise in linear reticular elasticity (Tharmann et al., 2007). In filamentous actin/filamin reticulations, the nonlinear response may be accounted for by the high flexibility of the discrete filamin molecules (Gardel et al., 2004; Gardel et al., 2006a). Moreover, in actin/filamin bundle reticulations, the branched and fused reticulation microstructure (Schmoller et al., 2008) could also be considered to account for the striking nonlinear viscoelastic response of the reticulation.

Contractile forces are extensively produced through the interaction of myosin II with actin filaments. GTP-bound RhoA upregulates myosin II activity through stimulation of Rho kinase (ROCK), that in turn increases phosphorylation of regulatory myosin light chain. This occurs in two ways, through direct phosphorylation of regulatory myosin light chain (Amano et al., 1996) and through the phosphorylation and consecutive blocking of the myosin light chain phosphatase (MYPT) (Kimura et al., 1996). Phosphorylation of myosin light chain increases the assemblage of myosin II into filaments and boosts its ATPase activity, which enhances the contractile force applied from myosin II to actin filaments. When myosin is arranged as filaments myosin, it acts as a prominent bundling protein for filamentous actin. Besides, ROCK phosphorylates and activates LIM kinase so that it can phosphorylate and restrain the actin-separating protein cofilin (Maekawa et al., 1999). Thereby, the actin filament stability is improved. RhoA stimulates also the continued assembly of actin filaments via its mDia effector, which represents an actin nucleating protein belonging to the formin family (Watanabe et al., 1999). Consequently, the RhoA signal transduction pathway is extensively accountable for a large part of intracellular force production in cells (Chrzanowska-Wodnicka and Burridge, 1996).

Cytoskeletal filament networks show viscoelastic behavior, since the disruption of the binding motif of filamin A that acts as an actin filament cross-linking protein. Additionally, the cryptic phosphorylation motif in β -catenin can be exposed by forces and thereby lead to viscoelastic behavior of the cytoskeleton. Moreover, the generation of catch bonds in α -catenin due to external stimulation can also cause elevated viscoelastic characteristics of cytoskeletal filament networks. Thus, this implies that coupling back between external and internal mechanical force may assist migrating cells to tailor their viscoelastic characteristics and migrate in physically confined microenvironments.

### 4.4 Nuclear Viscoelasticity

When a solid tumor expands due to cell proliferation, it generally gets denser, and the cancer cells are subject to a harsher physical environment. In part this is attributable to the deposition of more extracellular matrix, as well as cell proliferation resulting in tighter packed cells within the disposable tissue volume, and enhanced contractility of stromal cells (Paszek and Weaver, 2004; Paszek et al., 2005; Jaalouk and Lammerding, 2009; Yu et al., 2011). Cancer cells that metastasize are subjected to multiple forces, whether by invasion of individual cells or by collective cell migration (Friedl and Wolf, 2010). Squeezing across tight gaps in the extracellular matrix subjects cancer cells to substantial compressive and tensile forces, which can be adequate to induce transient break down of cell nuclei and liberation of chromatin, resulting in DNA damage (Denais et al., 2016; Raab et al., 2016). In addition to chemical cues that govern cell performance, mechanical forces can have a critical regulatory effect on cellular function. In response to various forms of mechanical forces applied to the cell membrane surface, multiple signaling cascades are excited. These include stretch-activated ion channels to activation of kinase cascades and Rho GTPases (Orr et al., 2006; Wang et al., 2009). In the end, multiple of such signaling pathways impact the transcription of genes.

#### 4.4.1 Role of Forces on Gene Expression

Small molecules and genetic interferences are frequently used to affect regulatory pathways, for instance pathways regulating calcium content or Rho GTPases (Loirand and Pacaud, 2014). The commonly used biochemical and genetic modifications cannot ensure the tight spatiotemporal monitoring of cell contractility. This complicates their applicability to discover how local elevation or attenuation of contractility might cause cellular or multicellular shape alterations. Optogenetics can be used to achieve reversible disturbance of intracellular biochemistry with subcellular resolution by expressing genetically engineered light-sensitive proteins. Optogenetics is a technique that has been utilized effectively to regulate the activity of ion channels, phospholipids, actin polymerization components and RhoGTPases (Bugaj et al., 2013; Deisseroth, 2011; Guglielmi et al., 2015; Idevall-Hagren et al., 2012; Levskaya et al., 2009; Rao et al., 2013; Valon et al., 2015; Wagner and Glotzer, 2016; Wu et al., 2009). Two optogenetic instruments that rely on monitoring the activity of endogenous RhoA to either up- or down-regulate the contractility of cells. These instruments elicit fast, spatial, and reversible alterations in traction forces, cell-cell forces, and the compaction of tissues. Thus, in addition, the changes in cellular forces are accompanied with translocation of the Yes1-associated transcriptional regulator (YAP), which is synonymously referred to as YAP1, indicating that optogenetics can be employed to govern mechanotransduction signaling pathways (Valon et al., 2017).

A candidate for regulating RhoA activity is the DHPH domain of ARHGEF11 (Rossman et al., 2005; Zheng et al., 2009), which fuses with CRY2-mCherry to form ARHGEF11(DHPH)-CRY2-mCherry, designated optoGEF-RhoA. To precisely govern the localization of this protein, two specific types of CIBN have been generated, one of which is directed to the plasma membrane (CIBN-GFP-CAAX) and one to the mitochondrial membrane (mito-CIBN-GFP). Through the application of an infrared RhoA biosensor composed of the rhotekin-binding domain (RBD) merged with infrared fluorescent protein (iRFP), it has been discovered that local enrollment of optoGEF-RhoA at the cell membrane corresponds to elevated activity of RhoA. Therefore, the optogenetic approach enables fast and reversible targeting of the catalytic domain of ARHGEF11 to the plasma membrane or mitochondria, which leads to supervised RhoA activity.

Additionally, it has been examined whether RhoA activation is accompanied with alterations in cell contractility after relocation of optoGEF-RhoA to the cell membrane or to the mitochondria. Hence, Traction Force Microscopy (Trepat et al., 2009) has been adopted to quantify the forces exerted by the cells on the subjacent soft collagen I-coated substrate (12 kPa, polyacrylamide) encountered throughout optogenetic activation and deactivation. To investigate the function of optoGEF-RhoA translocation toward the cell membrane, a MDCK cell line is genetically engineered that stably co-expresses optoGEF-RhoA and CIBN-GFP-CAAX. In order to determine the tension within a cell and among two neighboring cells, Monolayer Stress Microscopy has been utilized (Tambe et al., 2013). Similar to the traction forces, the cell tension grew in the exposed areas and stayed the same in the non-exposed areas. The growth in tensile force and tension has been maintained for a minimum of 40 min and, crucially, has been completely reversible. These findings demonstrate that mastering the subcellular localization of the catalytic domain of ARHGEF11 provides spatiotemporal inroads into the regulation of signal transduction and, consequently, cell contractility. The optogenetic enhancement of contractility has been coincident with the development of actin stress fibers (Valon et al., 2017). Reciprocally, cell relaxation has been associated with the vanishing of basal stress fibers. The pattern of spatial distribution of focal adhesions in the presence of alterations in contractility has been evaluated through co-transfection of optogenetic constructs and vinculin-iRFP. An augmentation of contractility failed to produce systematic modifications in the size or dispersion of focal adhesions. Optogenetic contraction and relaxation are hypothesozed to coincide with structural alterations in stress-generating and stress-sensing components of the cell.

To examine this hypothesis, the transcriptional regulator YAP has been selected, which has been reported to be translocated from the cytoplasm to the nucleus due to sustained elevated traction forces or increased substrate stiffness (Dupont et al., 2011; Elosegui-Artola et al., 2016). Therefore, it has been explored whether a sustained raise in cell contraction causes the translocation of YAP. For this purpose, MDCK cells are cotransfected with optoGEF-RhoA, iRFP-YAP, and CIBN-GFP-CAAX and exposed to pulsed illumination. Throughout this process, the intensity of iRFP-YAP within the nucleus has been assessed. The optogenetic enhancement of contractile forces has been accompanied with an enhancement of YAP in the nucleus. Consequently, optogenetic contraction and relaxation of cell contractility have antagonistic impacts on YAP translocation in the nucleus, suggesting that optogenetics can be employed to properly modify mechanosensitive transduction pathways and to interrogate potential transcriptional alterations. YAP is a major actor in the coordination of tissue growth, homeostasis, and cancer formation. It is controlled through two independent pathways. The first, entirely biochemical, incorporates the Hippo signaling pathway, while the second directly engages mechanosensitive signaling pathways that translate mechanical cues into biochemical cues.

#### 4.4.2 Role of Nuclear Characteristics on Cell Migration

Intriguingly, not merely the cytoskeleton and its elements account for the viscoelasticity of cells (Fischer et al., 2020). Apart from the cytoskeleton, other compartments of the cell, such as the biggest organelle of the cell, the cell nucleus contributes to the mechano-phenotype of the overall cell. It has also been revealed that the nucleus may be a constraining agent on the degree to what a cell would deform to pass through restricted cavities (Friedl et al., 2011; Calero-Cuenca et al., 2018; Fischer et al., 2020). The nucleus is enclosed by a nuclear membrane, beneath which is a dense meshwork of proteins referred to as the nuclear lamina, the principal constituent of which comprises the nuclear intermediate filaments, primarily made up of proteins referred to as lamins (Davidson and Lammerding, 2014; Fedorchak et al., 2014). Lamins are a vital part of nuclear-cytoskeletal interactions and mutations on these molecules affect the biochemical answer of cells to a mechanical impulse (Lammerding et al., 2004, Lammerding et al., 2005; Cupesi et al., 2010; Wolf et al., 2013; Cho et al., 2017). Lamin seems to directly function in the matrix-driven differentiation of tissues. High matrix stiffness values due to accumulation of collagen fibers cause high levels of lamin A and lead to the differentiation to bone, whereas low stiffness levels result in lower amounts of lamin and cause the differentiation toward fat tissue (Swift et al., 2013). Moreover, the cell nucleus serves a critical function in the progression of cell migration through constrained volumes, as it provides a mechanical barrier that impairs the amount of cell deformation that a migrating cell might encounter. Strictly stated, the nuclear lamina acts as a constraining element for the deformability of the nuclear core (Wolf et al., 2013; Calero-Cuenca et al., 2018). Whereas high levels of lamins provide a roadblock to deformation, lower levels permit a more flexible nucleus, but ultra-low levels of lamins restrict the survival of the cell (Lammerding et al., 2004; Cupesi et al., 2010; Ho and Lammerding, 2012). Among the mechanisms governing the density of the core lamina and the viscoelastic characteristics of the nucleus has recently been delineated through an elegant hybridization of microfabrication and quantitative biology techniques. Thereby, fast build-up of the actin nucleator Arp2/3 at the nuclear membrane of dendritic cells corresponds to instability of the nuclear lamina, which permits cells to deform their nuclei (Thiam et al., 2016), something that could be pertinent *in vivo*, where cells have to modify their viscoelasticity as they migrate through restricted gaps.

Cell transitions, such as EMT, lead to cytoskeletal alterations of cells (Yilmaz and Christofori, 2009) that can affect their extracellular matrix environment, since both the cytoskeleton of cells and the extracellular matrix are intrinsically coupled (Leggett et al., 2021). Consequently, cells are able to perturb the structure of the local matrix through deformation and remodeling in order to foster their migration and invasion.

#### 4.4.3 Viscoelastic Model of the Nucleus

The nuclear rheology relies on the time and length scales of the forced deformations that are determined by a variety of techniques ranging from substrate stretching (Lammerding et al., 2006), indentation (Schäpe et al., 2009; Krause et al., 2013), microneedle-based micromanipulation (Shimamoto et al., 2017; Stephens et al., 2019) and micropipette aspiration (Dahl et al., 2004; Guilak et al., 2021). This intricate viscoelastic reaction mirrors the wide range of both weak and strong interactions between lamins and chromatin (Dahl et al., 2005), which located at the nuclear envelope (Gruenbaum et al., 2005; Solovei et al., 2013) as well as in the nucleoplasm, and contributes to the stabilization of condensed chromatin regions inside lamin-associated domains (Guelen et al., 2008). Micromanipulation analysis yielded a common length scale of about 3 µm for the deformations, below which the elastic resistance of the nucleus is determined by chromatin, while the resistance to large deformations is governed directly through lamin A/C (Dahl et al., 2004; Stephens et al., 2019). In a similar way to isolated chromatin fibers, which stretch elastically (Cui and Bustamante, 2000), the rheological characteristics of nuclear chromatin fluctuate between predominantly elastic and predominantly viscous and are regulated via the nuclear envelope ligaments (Schreiner et al., 2015). The lamin A/C content varies more than the lamin B1 content between the cell types domiciled in the tissue to match the microelasticity of the tissue, thereby matching the stiffness of the nucleus with the extracellular stiffness (Swift et al., 2013). Concordant with the thin and low-density network of soft lamin filaments (Turgay et al., 2017), the lamina on its own, including both the A- and B-type lamina networks, imparts low mechanical strength on the nucleus (Panorchan et al., 2004; Banigan et al., 2017).

Micropipette aspiration can be used to determine the mechanical reaction of nuclei in intact cells across physiological length scales and loads. To assess the viscoelastic contributions of lamin A by itself, lamin-B1 by itself, lamin A and -B1 together, and lamin A phosphorylation levels, stable cultures of lamin knockout and lamin rescue mouse embryonic fibroblasts have been made. The mechanical function of chromatin has been analyzed with the aid of a compound pharmaceutical inhibitor of chromatin deacetylation, which reversibly causes chromatin decondensation. The integrated mechanical functions of lamins and chromatin have been dissected through RNA and protein profiling and transmission electron microscopy. A minimal linear viscoelastic model has been developed that examines the interrelated mechanical inputs of lamins and chromatin. A time scale is apparent of about 2 s that discriminates between two time-regimes that reveal distinct mechanical reactions of the nucleus to imposed stress. At short time periods, the nucleus expands elastically and effectively softens at long time periods. Effective nuclear stiffness is governed through lamin A and B1 and condensed chromatin. In cells expressing lamin A, decondensation of chromatin results in nuclear stiffening, possibly due to newly formed interactions with the nuclear envelope. In steady condition, the nucleus deforms viscously and is governed entirely by lamin A. The universality of the viscoelastic four-element model is not only shown for lamin expression patterns, phosphorylation levels, and chromatin condensation levels, but is also confirmed for nuclei of embryonic and pluripotent stem cells, whose lamin A and B1 levels and chromatin compaction are far reduced in comparison with fibroblastic cells. In the following the three-element models are presented and discussed. Creep test observations reveal complex viscoelastic reactions to applied loading that are a function the expression and phosphorylation of lamin, and the decondensation of chromatin. Cell nuclei across at each conditions display the following features. In the elastic reaction, the nucleus expands instantaneously at the moment the stress is imposed, similar to a spring. In viscoelastic stretching, the nucleus is viscoelastically sucked into the micropipette over a typical time scale. For viscous deformation, nuclear creep converges to a steady rate. This specific mechanical reaction has been seen at both low and high strain and is commonly used by mouse embryonic stem cells and induced pluripotent stem cells, although the organization of nuclear laminae and chromatin is quite dissimilar to that of mouse embryonic fibroblasts (Melcer et al., 2012; Schlesinger and Meshorer, 2019).

All pertinent three-element viscoelastic models consistently could not accurately account for the principal characteristics of the nuclear reaction to imposed forces under all constraints within the measured time interval. The minimum linear viscoelastic model that correctly reproduced the nuclear deformations under all constraints has been the four-element Burger model. Specifically, the Maxwell and Kelvin illustrations of the Standard Linear Solid model are in fact the only two three-element models that account for instantaneous deformation. Therefore, their usefulness in modeling the aspiration-creep compliance curves of micropipettes is analyzed and compared with Burgers model. The Burgers model yielded the highest R-squared values for quality of fit, not just because it is composed of four items. It also accounts for the viscous deformation of the nuclei at stationary state, in contrast to the standard Linear Solid models. Actually, both standard linear solid models exhibited weaker fits for nuclei expressing lamin B while lacking lamin A, due to their long-term low viscosity deformation characterized by high steepness while maintaining stiffness.

The response of the Burgers material enables the nucleus to accommodate the applied impacts through elastic elongation and relieve the sustained stresses through viscoelastic deformation to avoid cracking and breaking of the genome and lamella (Dahl et al., 2004). Natural and synthetic biomaterials consisting of cross-linked filaments frequently demonstrate nonlinear mechanics and, in particular, stiffening (Kang et al., 2009). To investigate nuclear performance under various loading levels, micropipette aspiration of WT and TKO cells is conducted with low (below 0.8 kPa) and high (over 1.6 kPa) aspiration pressure. At both pressure levels a Burgers reaction has been seen. Nuclear compliance is reduced with raising load. WT nuclei can be matched using higher stiffness terms and viscous terms, while the response time *τ* stays unchanged. The relationship between WT and TKO remained the same between low and high load conditions. Stiffness is defined as being governed by both lamin A and lamin B1, while viscosity is governed predominantly through lamin A (Wintner et al., 2020). Consistent with this, the viscoelasticity of chromatin has been determined using dynamic measurements in living cells, that is also viscoelastic modeled after the Langevin equation and an approximation of the harmonic potential encountered at each chromatin location (Vivante et al., 2020).

The viscoelastic characteristics of chromatin have also been measured by performing long-range coherence analysis of histone dynamics inside HeLa cells (Zidovska et al., 2013). The coherence has been found in the order of micrometers, which is larger than the typical dimension of a chromosome territory, and it has also been accounted for by the elastic characteristics of chromatin. When chromatin exhibits considerable elasticity, it can transfer local forces over large spans, even extending beyond the size of a chromosome area, which could lead to coherent movements of broad areas of chromatin. This has also been investigated theoretically, demonstrating the time-space crosstalk of chromatin loci (Lampo et al., 2016). Therefore, a coarse-grain model of chromatin has been proposed, where every two monomer segments are linked through springs, along with a hydrodynamic resistance in the nucleoplasm. This underlines the significance of the elasticity of the polymer in conjunction with the viscoelastic characteristics of the nucleoplasm.

## 5 Coupling Between Viscoelasticity and Cellular Motility

Mobility, growth, and homeostasis are regulated by viscoelastic or material properties of cells and tissues (Huber et al., 2013; Barriga and Mayor, 2019; Burla et al., 2019; Petridou and Heisenberg, 2019; Chaudhuri et al., 2020). Viscoelasticity permits living systems to maintain a fundamental architecture because of their solid-like features, while dynamically rearranging themselves in various shapes and configurations based on their viscous properties (Lecuit et al., 2011; Pegoraro et al., 2017; Petridou and Heisenberg, 2019) that is linked to the migratory capacity of cells (Petridou and Heisenberg, 2019). Cellular viscoelasticity affects several single-cell properties including shape, division, and motility and is mainly governed by the physical characteristics of the underlying cytoskeletal meshes (Pegoraro et al., 2017). Tissue-level viscoelasticity has been found to matter in collective morphogenetic events involving tissue folding, spreading, wound healing, and migration, and is mostly dictated by the interaction of cell-cell and/or cell-extracellular space relationships (Calvo et al., 2013; Barriga and Mayor, 2019; Petridou and Heisenberg, 2019).

Resembling the viscoelasticity of nonliving materials, the viscoelasticity of cells and tissues is a characteristic that emerges from the underlying architecture and is delineated by how macromolecules and cells interface (Bi et al., 2015, Bi et al., 2016; Broedersz and MacKintosh, 2014; Farhadifar et al., 2007; Huber et al., 2013; Pegoraro et al., 2017; Petridou et al., 2021; Pritchard et al., 2014). In nonliving materials, various theoretical approaches have long been employed to connect microscopic texture to macroscopic viscoelastic characteristics (Box 1), demonstrating that viscoelasticity can act as an emergent characteristic (Alvarado et al., 2017; Bi et al., 2015; Bi et al., 2016; Gardel et al., 2004; Kim et al., 2017; Petridou et al., 2021). Similarly, temperature, for instance, arises nontrivially from microscopic particle movement in statistical mechanics. How macroscopic viscoelasticity can be modeled through the interactions of the microscopic components of living cells and tissues is an outstanding question at the border between physics and molecular and cellular biology.

An interesting empirical finding is that the material characteristics of the microscale constituents of cell and tissue viscoelasticity, for instance, the components of the cytoskeleton and the cells, respectively, generally do not correspond to the macroscale material characteristics of cells and tissues (Alvarado et al., 2017; Broedersz and MacKintosh, 2014; Petridou et al., 2021). Macroscopic viscoelasticity often displays nonlinear variations that are not evident at the microscopic scale. However, experimental evidence has been provided for such examples like the stiffening reaction of the cytoskeletal reticulum (Gardel et al., 2004; Gardel et al., 2006b; Pritchard et al., 2014), phase transitions charges of cell motions (Mongera et al., 2018) or sudden alterations in tissue viscosity (Petridou et al., 2019) (Box 1). In the aforementioned contexts, the mechanical resistance of the individual microscopic constituents to forces is not sufficient to account for the macroscopic viscoelastic variations, so instead it is important to examine the overall interaction scheme among the constituents. For example, experimental measurements of the viscoelasticity of cells and tissues typically involve applying an external force to the system, for instance employing a micropipette or a magnetic field (D’Angelo et al., 2019; Serwane et al., 2017), for a specific time period in which the deformation of the cell or tissue system is overseen. Parameters like elastic modulus, viscosity and yield stress (Box 1) can be derived from these experiments (Forgacs et al., 1998; Bonn et al., 2017).

At the nuclear scale, a link between the mechanical characteristics of the nucleus and cancer metastasis has recently begun to evolve. The nucleus, the largest cellular organelle, is quite stiff in normal cells, but once it becomes less elastic, it can be more readily reshaped and ceases to act as an interfering barrier when the cell undergoes metastasis across narrow capillaries (Contu et al., 2018; Denais and Lammerding, 2014).

### 5.1 Viscoelasticity Drives Cell Migration and Invasion of Single Cells

The capacity of eukaryotic cells to withstand deformation, conduct intracellular trafficking, and alter their shape during locomotion relies on the cytoskeleton, an interconnected reticulation of filamentous polymers and regulatory proteins. Some recent efforts have indicated that both internal and external physical forces can function through the cytoskeleton to affect local mechanical characteristics and cell performance. The mechanics of eukaryotic cells are dominated by the cytoskeleton, a composite polymer network that embraces the whole cell and affords the cell both the stability necessary to resist external forces and the versatility to wriggle through tissue spaces (Fletcher and Mullins, 2010). The mechanical characteristics of single cells seem to determine their mode of migration, the directionality of the movement, movement dynamics and migratory capacity including invasion path length. The mechanical properties of cells additionally contribute to their force exertion on their extracellular matrix environment, such as causing the alignment of extracellular matrix fibrils, extracellular matrix degradation by release of enzymes, such as matrix metalloproteinases, or cross-linking of fibrils by cell-derived extracellular matrix proteins, such as fibronectin or fibrinogen.

The viscoelastic reactions of cancer cells to the stiffness of collagen matrices have also been determined through analysis of intracellular rheology with optical trapping, and overall cell stiffness through the deformation analysis of cells, when released from the collagen matrix and pushed through a microfluidic channel (Wullkopf et al., 2018). In this study, alterations in both time-dependent intracellular viscoelasticity and total cell stiffness are detected in several cell lines. Comparing several cancer cell lines, it is concluded that cancer cells with high invasiveness can modify their stiffness in reaction to matrix stiffness, whereas less invasive cells do not react to stiffness.

### 5.2 Hallmarks of the Protrusive-Type of Single Cell Migration

Are cellular protrusions viscoelastic? To answer this question the viscoelastic characteristics of cellular protrusions has been explored using an optical tweezer setup (Khatibzadeh et al., 2013). The force-length diagrams connected with protrusions exhibit nonlinear proportions (Khatibzadeh et al., 2013). This finding is ascribed to the rupture of the bonds between the plasma membrane and cytoskeleton with subsequent flux of membrane lipids into the protrusion, and to additional viscous events due to slippage between the plasma membrane and cytoplasm encountered during the formation of a protrusion (Waugh and Bauserman, 1995; Hochmuth, 2000; Marcus and Hochmuth, 2002; Borghi and Brochard-Wyart, 2007). The existence of viscous interferences and bond breaks manifests itself as hysteresis in the reverse-pull examinations. The W_loss_ in control cells is near that onserved in human neutrophils (Xu and Shao, 2008). This analysis suggests the cytoskeleton dependence of energy loss in protrusion generation, as indicated in the contraction of the hysteresis circuit and the significant decline in W_loss_ in control cells following discontinuation of F-actin. The increased energy efficiency of protrusion generation in latrunculin-A treated cells is likely to be the outcome of less cytoskeletal-membrane connectivity in conjunction with the reduction in protrusion viscosity.

The Standard Linear Solid model matches the force-length diagrams in cells with intact F-actin and F-actin-destroyed cells, which leads to the suggestion of the viscoelastic response of the protrusions. To explore the viscoelasticity of the protrusions even further, the force relaxation of the protrusions has been investigated. For this purpose, in a reverse-pull procedure, protrusion pulling has been halted at the end of the pulling procedure, leading to an instantaneous relaxation of the protrusion force in a cell with intact F-actin. Force relaxation persisted to the point of reaching an equilibrium level prior to pushback. The presence of both force relaxation and hysteresis phenomena in these protrusions suggests the viscoelastic response of the protrusions (Xu and Shao, 2008). The force relaxation curve has been fitted with the Standard Linear Solid model. The reduced levels of protrusion viscosity seen with F-actin disruption are in agreement with previous findings showing the dose-dependent diminishing impacts of F-actin breakdown on cytoplasmic viscosity (Marion et al., 2005; Tsai et al., 1994). The lower viscosity and more fluid response of the cytoplasm in F-actin-perturbed cells may be due to the lesser counteracting influences of the cytoskeleton microfilaments on the motion of the cytoplasm. Thus, the generation of longer protrusions before membrane-cytoskeleton disconnection in F-actin-perturbed cells and cholesterol-enriched cells may be attributable to their accelerated growth during the formation of the protrusion as a consequence of their lower viscosity levels. This result is in agreement with the simulations predicted by the Standard Linear Solid model, according to which protrusions with lower viscosity coefficient values are linked to longer lengths at the same pulling force. By the same means, protrusions with lower stiffness levels, seen in cells with F-actin breakage and cholesterol-enriched cells, are accompanied by longer protrusions for the identical force. Higher stiffness values of the protrusions either in the early phase of protrusion generation or towards the end of protrusion elongation, as induced for example by cholesterol depletion, cause shorter protrusions according to the Standard Linear Solid model. Consequently, the plasma membrane and cytoskeleton reciprocally participate in the viscoelastic response of the cellular protrusions. The effects of the mechanical characteristics of the plasma membrane are quite small compared to the actions of the cytoskeleton; however, unexpectedly, modulation of membrane constitution yielded significant modifications in the mechanics of protrusion.

**FIGURE 3:**
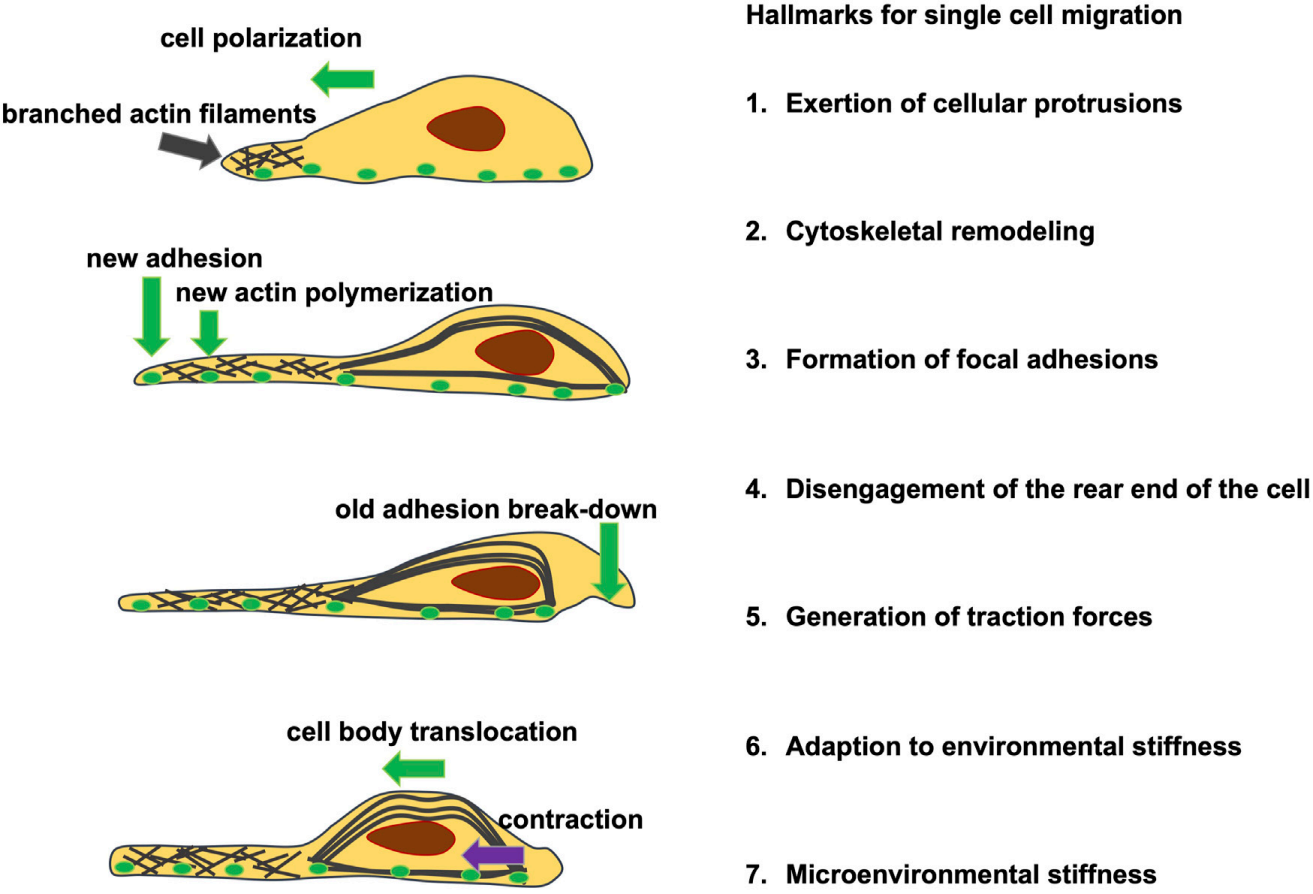
Hallmarks of single cell migration.

The movement of cells can be subdivided into motility and migration. Motility is a spontaneous, undirected movement, whereas migration involves a directed movement occurring in reaction to a cell attracting or repelling agent (Kwon et al., 2020). However, many manuscripts today fail to take this into account and even confuse it. Cell movements extend from the uncoordinated rippling of cell boundaries to the migration of individual cells (Ridley, 2003) and to the collective movements of contiguous groups of cells (Friedl and Gilmour, 2009). Single-cell migration in the mode of protrusive migration allows cells to relocate to and between tissue compartments, a distinct process that is a key driver of inflammation-related leukocyte migration (Friedl and Weigelin, 2008). Specifically, cell migration is controlled by alterations in the cytoskeleton and the forming of focal adhesions. Cell migration comprises the subsequent set of events: firstly, protrusion of the leading edge, secondly, creation of focal adhesion at the front boundary and dislodgement at the trailing boundary, and thirdly, motion of the cell body (Murphy-Ullrich, 2001; Lindberg et al., 2008). In the course of migration, actin is polymerized at the leading boundary protrusion and repeatedly depolymerized at the trailing boundary. Polymerization and bundling of F-actin results in stiffening of the cells, whereas depolymerization softens the cells (De La Cruz and Gardel, 2015). Following actin polymerization at the protuberance at the cell front, adhesions are formed close to the front edge. The adhesions ripen by dynamic cross-linking of F-actin. The adhesions then dissolve at the trailing end when the connection between F-actin and integrin is disrupted. The physical interplay between F-actin and integrin delivers the pulling force required for cell migration. Finally, the hallmarks of “exertion of cellular protrusions,” “cytoskeletal remodeling,” which includes actin polymerization and cross-linking, “formation of focal adhesions,” “disengagement of the rear end of the cell” and “generation of traction forces” can be stated for the individual cell migration (Figure 3).

The development of technologies to assess cellular elasticity, such as AFM and micropipette aspiration, indicates that elasticity and motility of cancer cells are mutually correlated, and elasticity has been acknowledged as a biomarker for the invasive potency of cancer cells (Wagh et al., 2008; Krause and Wolf, 2015). The relationship between cell elasticity and metastatic capacity has been established; however, several reports contradict one another (Kwon et al., 2020; Mierke, 2014; Mierke, 2019; Mierke, 2020). For instance, highly metastatic ovarian HEY A8 cancer cells are softer than non-malignant ovarian epithelial cells, and the migratory potential of HEY A8 is coupled to the restructuring of the actin cytoskeleton (Xu et al., 2020). Contrary, stiff breast MDA-MB-231 cancer cells have displayed excellent migratory capacity within dense culture matrix settings (Messica et al., 2017). The features of adhesion towards the extracellular matrix of cancer cells deviate from those of normal cells and are also linked with invasive and metastatic capacity of cancer cells (Khalili and Ahmad, 2015). Adhesion strength is commonly diminished in cancer cells, and the changes vary according to cell type and oncogene (Zou et al., 2002; Spangenberg et al., 2006). Adhesion strength is found to be heterogeneous in metastatic cells under stromal-like settings due to their enhanced sensitivity to Mg^2+^- and Ca^2+^-driven breakdown of focal adhesions (Fuhrmann et al., 2017). When the cells adhere heavily to the extracellular matrix, their migration is abolished.

Despite this wide variety of migration modes, there seems to be a general consensus that all demand (to different extents) the below inputs: firstly, cell polarization, restructuring of the cytoskeleton, and generation and exertion of forces founded on the interaction between actin polymerization and contraction of actomyosin scaffold. Secondly, cell-cell adhesion and linkage through adherens-junction proteins that are connected the actin cytoskeleton. Thirdly, control through by chemical and physical cues. The fundamental functions realized by these various determinants endow cells with the capacity to generate forces, to adhere possible also in different ways to one another and to a substrate, and to be sensitive to mechanical and chemical cues. Nevertheless, it is still not fully understood how these fundamental characteristics are implemented during the migration of single cells. However, there exists an active self-regulation of the cytoskeleton. Assembly and disassembly of cytoskeletal scaffold and architectures are governed through multiple accessory proteins (Lauffenburger and Horwitz, 1996; Ridley, 2003). These regulating proteins constitute a reaction reticulum with several feedback mechanisms that enable the structures of the cytoskeleton to react to outside mechanical irritants (Marée et al., 2012, Marée et al., 2006). In addition, cytoskeletal structures, such as focal adhesions underneath integrins seem to fulfill a critical task in the spatiotemporal guidance of these regulatory proteins (Schwartz and Shattil, 2000).

Stem cell differentiation is guided by microenvironmental stiffness and thereby cells adapt to the stiffness of the substrate on which they are cultured and adhered to (Discher, 2005), which represents a hallmark of cell adhesion and migration. This hallmark can be termed “Adaption to environmental stiffness.” In addition, cells move towards stiffer or softer environment (Pelham and Wang, 1997), which leads to another hallmark of cell migration. It can be stated as “Microenvironmental stiffness.” All of these findings may also be transferable to cancer cells in the malignant progression of cancer.

## 6 Regulation of Transcription Leads to Changes of the Cellular State

There is strong compelling support for bidirectional mechanical signaling across the nucleus and the cytoskeleton. The mechanical characteristics of the nucleus have been the focus of mechanical analyses of cells for a while, especially when mutations in lamin A/C appeared to underlie a number of genetic diseases with a mechanochemical foundation (Zwerger et al., 2011). The concept of mechanically linking the nucleus to the cytoskeleton has long been presented (Graham and Burridge, 2016). Electron microscopic analysis of cytoskeletal assemblies indicated that they appear to surround and be linked to the outer nuclear membrane (Capco et al., 1982). Tension applied to the cell surface results in nuclear distortion, corroborating that tension is transferred to the nucleus through the cytoskeleton (Gundersen and Worman, 2013). Moreover, the position of the nucleus varies according to cell type and is determined by interactions of the cytoskeleton (Gundersen and Worman, 2013). Great efforts have been made to pinpoint the proteins that link the nucleus to the cytoskeleton. Through a combination of techniques, the linker of nucleoskeleton and cytoskeleton (LINC) complex has been revealed (Crisp et al., 2006; Wang et al., 2009). The principal constituents of the LINC complex comprise members of the Nesprin protein family (Rajgor and Shanahan, 2013). They include transmembrane proteins that span the outer nuclear membrane and connect to SUN proteins in the intermembrane cavity between the outer and inner nuclear membranes. The SUN proteins span the inner nuclear membrane and connect to the nuclear lamins and other proteins, including emerin. As they elongate into the cytoplasm, the nesprins attach directly or indirectly to the actin, microtubule, and intermediate filament cytoskeletons (Rajgor and Shanahan, 2013). Imposing tension on isolated nuclei through tugging on the nesprins resulted in the stiffening of the nucleus, validating that the nucleus is mechanosensitive and reacts to tension conveyed over the cytoskeleton and the LINC complex (Guilluy et al., 2014). How can the nucleus, as a fairly stiff intracellular organelle physically linked to the cytoskeleton, influence mechanotransduction and facets of cell behavior, including cell migration? It is known that the tension imposed on the nucleus influences the transcription and the distinguished phenotype of the cells, which is shown below.

### 6.1 Separation of the Link Complex

There are two different strategies that can be implemented to separate the nucleus from the cytoskeleton: total excision of the nucleus (enucleation) and separation of the LINC complex through expression of dominant-negative KASH domains, dominant-negative SUN proteins, or exhaustion of LINC complex constituents (Crisp et al., 2006; Stewart-Hutchinson et al., 2008; Lombardi et al., 2011). The expression of dominant negative KASH domains, which can compete with Nesprin for the interaction with SUN proteins, causes altered mechanical phenotype of the cells (Stewart-Hutchinson et al., 2008; Lombardi et al., 2011). Rheological measurements showed reduced stiffness of the cytoplasm of transfected cells (Stewart-Hutchinson et al., 2008), and changed force transduction through the cell and reduced deformation of the nucleus upon reaction to a surface force applied to the cell (Lombardi et al., 2011). Alterations in the perinuclear architecture of actin stress fibers have been observed. Moreover, disruption of the LINC complex in this manner also reduced the speed of cell migration and the directional persistence of cellular motility. The directional persistence may be due to altered cell polarity resulting from perturbation of the centrosomal/nuclear axis, which has been noted in reaction to the expression of dominant-negative LINC complex constructs (Lombardi et al., 2011). Reverse nuclear motion and centrosome realignment in cells migrating into a scratch wound is inhaled by the dominant-negative KASH domains (Luxton et al., 2010).

The nuclear lamina, which forms the basis of the inner nuclear membrane, is linked to the cytoskeleton through the LINC complex and is accountable for the majority of the stiffness and form of the nucleus. Deletion of the gene lamin A (LMNA^−/−^) showed that this lamin is especially relevant for these mechanical characteristics (citations...) (Broers et al., 2004; Dahl et al., 2004; Hale et al., 2008; Lammerding et al., 2004, Lammerding et al., 2006). In a similar manner to the expression of dominant-negative KASH domains, deletion of the lamin A gene impacts not exclusively nuclear mechanics, but cytoskeleton, cell polarity, and cell migration as well (Hale et al., 2008; Lee et al., 2007). Expression of lamin A mutant constructs similar to those involved in muscle diseases also recapitulated these findings (Hale et al., 2008; Folker et al., 2011; Zwerger et al., 2013). Even though no alterations in stress fiber organization occurred in the Lmna^−/−^ cells, their focal adhesions seem to be shorter (Hale et al., 2008). The RhoA activity has been found to be decreased Lmna^−/−^ cells (Hale et al., 2008). RhoA activity has been seen to be reduced in cells that express a laminopathic Lamin A mutant, however, no alterations in the focal adhesions have been observed. This could be due to the fact that RhoA activity remained over a critical level of threshold.

### 6.2 Removement of the Nucleus From Cells (Enucleation)

Expression of compounds of the dominant-negative LINC complex or their genetic removal are accurate tools to examine the contribution of the connections linking the nucleus to the cytoskeleton. A much coarser attempt is the total excision of the cell nucleus. This is a crude approach but it has the benefit of destroying all links to the nucleus, whereas cutting the LINC complex can still keep intact other types of interactions of the cytoskeleton to the nuclear envelope. Large-scale enucleation strategies for cells maintained in culture have been established in 1972 (Prescott et al., 1972). Enucleated cells, known as cytoplasts, have been noted to harbor multiple organelles and can last for hours or days, according to the cell type.

When techniques for enucleation first emerged, there has been just no examination in mechanotransduction. The question of whether the nucleus has any influence on the migratory behavior of a cell arose. It has been established that cytoplasts are able to migrate on glass, demonstrating that ownership of a nucleus is not a requirement for cell migration (Goldman et al., 1973). This finding has been validated in which the migratory properties of cell fragments without nuclei have been investigated (Shaw and Bray, 1977). In the same way, very small membrane-bound fragments of fibroblast cytoplasm (microplasts) exhibited several motile behaviors, including membrane rippling, filopodia elongation and retraction, and membrane blebbing behavior (Albrecht-Buehler, 1980). Investigating this phenomenon in more detail, such fragments have been seen to adopt either a non-polarized symmetric disk-like shape or a polarized arrangement. In the nonpolarized condition of the morphology, they exhibited no migration, while in the polarized condition they displayed persistent directional migration (Verkhovsky et al., 1999). Specifically, the directional migration in the non-polarized fragments can be induced by exerting a mechanical force toward one side. These previous investigations showed that a wide variety of cell types can perform efficient migration even in the absence of a nucleus. The possible role of the nucleus in the 3D migration of cells or the extent to which the nucleus participates in cellular mechanotransduction has not been analyzed. However, it has been pointed out that the nucleus has a major contribution to 3D migration (Wolf et al., 2013). Employing cytoplasts from fibroblasts or endothelial cells, it has been seen that they can migrate on two-dimensional (2D) surfaces (Graham et al., 2018). n addition, cytoplasts have been found to recognize gradients of growth factors and extracellular matrix and to undergo chemotaxis and haptotaxis. When examining migration in 3D collagen gels, cytoplasts exhibited minimal net migration. Nevertheless, the cytoplasts showed the ability to extend protrusions into the ambient 3D matrix without migration (Graham et al., 2018). Why are cells without nuclei able to migrate so well on 2D surfaces but so limited in 3D collagen matrices? In addition to the difference in dimensionality, another experimental distinction is that migration on two-dimensional surfaces is usually studied on very stiff substrates, whereas migration in 3D matrices uses relatively soft substrates. An explanation for the lower migration of cytoplasts in three dimensions arises from their lower capacity to migrate effectively on or in soft supports (Graham et al., 2018).

The impact of enucleation on the migration velocity of intact cells has been imitated by blocking myosin II activity with the inhibitor blebbistatin (Graham et al., 2018). Blebbistatin treatment of intact cells postponed the peak migration speed to stiffer substrates, indicating that enucleation may impair myosin activity and cell contractility. A similar effect of myosin blocking has increased the cell migration rate on soft substrata (Ulrich et al., 2009). This effect of blebbistatin on cytoplasts implies that excision of the nucleus impairs the overall contractility and mechanotransduction of the cells. Moreover, it has been shown that cytoplasts can only weakly contract collagen matrices and displayed decreased traction force on underlying medium in comparison to cells (Graham et al., 2018). Cytoplasts exhibited reduced stiffening due to pulling on magnetic fibronectin-coated beads. Thus, it can be concluded that excision of the nucleus decreased cell contractility and mechanotransduction (Graham et al., 2018).

What is the reason of reduced RhoA activity in cytoplasts? This needs to mirror either reduced GEF or enhanced GAP activity. This question has not yet been clarified. A possibility is that nuclear elimination depletes specific GEFs, such as ECT-2 and NET-1, that are localized in the nucleus. The extent to which these GEFs account for the total level of RhoA activity in cells is not clear. Another option is that a stiff nucleus connected to the actin cytoskeleton is a major driver of the total tension in the cell. As tension enhances the activities of multiple GEFs and conversely reduces GAP activity, reducing tension by excising the nucleus in this model is expected to lead to a decrease in RhoA activity. This is corroborated from the finding that cells missing lamin A have a soft nucleus, lower tension, and lower RhoA activity (Hale et al., 2008). In a similar manner, it has been inferred that the separation of the nucleus from the cytoskeleton due to the disconnection of the LINC complex leads to a reduction in the active RhoA (Thakar et al., 2017). Apart from the LINC complex and enucleated cells, the DNA itself can serve as a biomaterial scaffold (see above).

### 6.3 Entanglement of Viscoelasticity, EMT and Governance of EMT Transcription Factors

The entanglement of cells is required for cell movement and viscoelasticity seems to be a prominent player. The analysis of viscoelasticity is still focused on cell-cell adhesions rather than on the mechanical characteristics of individual cells. The latter seems to demand still more intensive research effort. However, it is well-known that the EMT relies on the deformability of cells. To account for the viscoelastic properties of cells, a three-dimensional model has been built integrating the viscoelastic characteristics of the cytoskeleton and the membrane-cortex compound (Karcher et al., 2003). Apart from cellular viscoelasticity, the magnitude of the ambient viscoelasticity impact transcends cell response, and more than one research effort has demonstrated that extrinsic mechanical irritants result in alterations in gene expression. At the transcriptional and posttranscriptional levels, adherens junctions proteins have been identified to be functionally modified by transient and reversible EMT. Nevertheless, the mechanisms underpinning these alterations in cadherin levels and the interaction of environmental mechanics with EMT-related transcription factors are still just starting to be grasped. Transcription factor Zeb, Twist, and Snail families are EMT transcriptional regulatory agents that govern cadherin mRNA levels. Twist expression has been revealed to be mechanically adjusted during gastrulation of *Drosophila*, with exogenous compressive forces elevating the ectopic expression of twist (Farge, 2003; Desprat et al., 2008). Intriguingly, the subcellular homing of Twist has also been reported to be mechano-regulated in 3D-cultured MCF10A and Eph4Ras cell lines. The nuclear localization of twist can be determined when MCF10A and Eph4Ras cells are grown on rigid materials, nonetheless, within soft materials twist is primarily localized inside the cytosol (Wei et al., 2015). The EMT transcriptional regulator slug, which is a member of the snail family, has also been predicted to be mechanically modulated. A vimentin-facilitated mechanism of the mechanical stimulation of slug has been suggested (Liu et al., 2015a). The expression of E47, which is also an EMT transcription factor, and the nuclear targeting of SNAIL and its expression are also decreased during the impairment of the actomyosin contractility (Lee et al., 2011; Lee and Nelson, 2012). The underlying mechanism whereby mechanical cues are brought internal to direct the expression of these transcription factors is still an unresolved concern. Yap1, a compound of the Hippo signaling complex, is a prominent converter of mechanical environmental cues into biochemical pathways (Dupont et al., 2011; Calvo et al., 2013). Moreover, the Yap1 activity has been coupled to EMT, since Yap1 can largely interference with transcription factors that foster the transition of EMT. For instance, the EMT transcription factor ZEB1 turned into a transcriptional activator once it started interfering with YAP1 in more aggressive cancers (Lehmann et al., 2016).

Specifically, YAP and WW domain-containing transcriptional regulator 1 (TAZ), synonymously referred to as WWTR1 (together YAP/TAZ), are transcriptional regulators that react to various mechanical stimuli, encompassing extracellular matrix stiffness, cell form, and shear stress (Panciera et al., 2017). Although there is recent evidence for a type of crosstalk between YAP/TAZ and Notch during angiogenesis (Neto et al., 2018), the specific nature of the crosstalk’s involvement is awaiting further exploration. Additional specifics have been elucidated in other tissues. During myogenesis, for instance, the initiation of contractions triggers the relocation of YAP to the nucleus, where it propels the expression of Jag2 and ensures Notch activation (Esteves de Lima et al., 2016), which is connected to the continuation of the progenitor cell stock necessary for regeneration (Bröhl et al., 2012), whereas YAP/TAZ adjusts the expression of Notch-inhibitory ligands within epidermal stem cells (Totaro et al., 2017). The segmentation clock, a molecular oscillator that controls the correct segmentation of developing somites, combines YAP and Notch to link mechanical signals to molecular cues and synchronize cell performance across the segmenting tissue (Hubaud et al., 2017).

**FIGURE 4:**
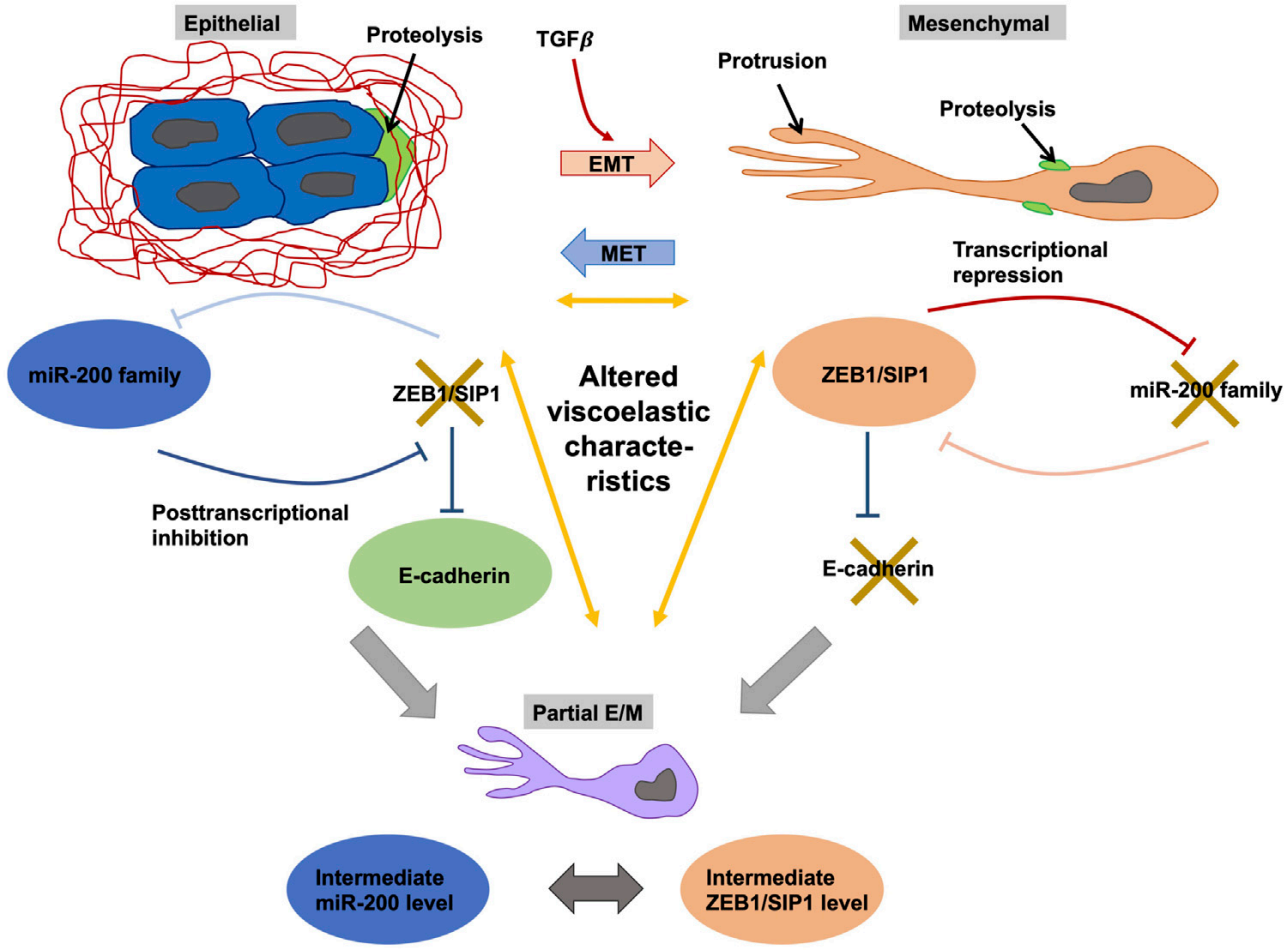
Interplay of transcription factors and mechanical characteristics in EMT and partial EMT.

However, TGFβ interaction with SMAD proteins is critical for the activation of SNAIL, SLUG, and TWIST on a transcriptional level (Figure 4). The stability of this TGFβ-SMAD complex has been revealed lately to be conveyed through Yap1. It is not yet clear how Yap1 can be targeted toward the nucleus and it has been proposed that force-driven opening of nuclear pores seems to be a major mechanism of Yap1 nuclear translocation (Elosegui-Artola et al., 2017) and possibly also of other transcription factors. These instances of interaction between mechanotransducers and EMT transcription factors highlight just a single of the many feasible mechanisms that migratory cells can utilize to convert mechanical cues into a molecular and cellular answer. These kinds of specific interactions can be the basis of other emergent features of collective migration that are related to cell-cell interfaces. Cell-cell adhesion has been revealed to be necessary for collective plithotaxis, a mechanism that imparts an “innate” directionality to cell monolayers (Trepat and Fredberg, 2011). Whereas the involvement of these canonical transcription factors in the control of EMT *in vitro* and *in vivo* is well settled, the involvement of mechanical interactions is comparatively less comprehended. Whether environmental viscoelasticity acts to modify EMT by influencing other facets of this intriguing event, such as apicobasal polarity, cadherin turnover, and MET, is just becoming unraveled.

### 6.4 Role of Viscoelasticity in the Epithelial-To-Mesenchymal Transition of Single Cells

Cellular viscoelastic characteristics are likely to fluctuate according to the biological state of the cells, for example, during the EMT transition in cancer, and thus viscoelasticity has the potential to be a beneficial physical biomarker (Nguyen et al., 2020). EMT represents an initial stage for individual cells and has originally been portrayed and defined as a process in which cells of a stalled epithelium shed their apicobasal polarity and cell-cell adhesion characteristics. Eventually, they become individual cells and migrate between tissues with a mesenchymal phenotype. On the length scale of the adherens junctions, a hallmark of this “canonical” EMT is a severe decrease in type I cadherin (E-cadherin) and, in a few cases, an augmentation of type II cadherins or the expression of type I cadherins with decreased adhesion strength, such as N-cadherin (Ruiz and Guenthert, 1996). Transcription factors of the Zeb, Snail, and Twist families are well-known as canonical upstream transcriptional regulators of this switch of cadherin (Figure 4) (Ansieau et al., 2014; Puisieux et al., 2014; Goossens et al., 2017).

This definition has been extremely informative in the initial phases to uncover the fundamentals of EMT, though most of this detail stems from experiments made in 2D *in vitro* settings and fails to consider the various tactics that cells and cell groups employ to migrate through divergent environments *in vivo*. The investigation of EMT has revealed that EMT may no more be handled as a linear, unidirectional event that switches the motility of single cells, but is instead now considered as a more dynamic event by which cells or sets of collectively migrating cells can accommodate themselves to the physical demands of their microenvironment. Several cues are known about the EMT. Firstly, EMT seems to be a reversible event and cells can also perform mesenchymal to epithelial transition (MET) (Jo et al., 2009; Kim et al., 2017). Secondly, apart the fact that EMT does not merely foster motility, it additionally aids cells to keep their stemness (Downing et al., 2013). Thirdly, migration can also take place even when cells display high amounts of E-cadherin (Li et al., 2016). Fourthly, EMT not only enables individual cell migration, but also aids later in collective epithelial and mesenchymal migration (Campbell and Casanova, 2016). It can be hypothesized that the plasticity of individual and collective migration may be conducted by providing cells with tunable viscoelasticity features that then lead to alterations of their adherens junctions. The adherens junctions alterations take place at a transcriptional and/or posttranslational regime, through alteration of expression and breakdown dynamics of adherens junctions proteins (Barriga and Mayor, 2015). Moreover, these alterations have been linked to the new general network for EMT (Brabletz et al., 2018; Campbell and Casanova, 2016; Mierke, 2019; Nieto et al., 2016). Finally, the classical view on cell migration that is based on the hypothesis that cells can either migrate in a collectively manner as epithelial cells (Friedl and Gilmour, 2009; Rørth, 2009), or perform an EMT and migrate in a single manner as mesenchymal cells (Thiery et al., 2009). This traditional division into two distinct migration phenotypes is essentially based on the classical view of EMT as a dual binary choice entailing the switch from a completely epithelial to a completely mesenchymal state (Hay, 2005), and on the idea that a true mesenchymal cell migrates singly through the extracellular matrix (Acloque et al., 2009; Nieto, 2011). However, this classical view has been challenged in developmental biology because the rich diversity of migratory incidents that arise during animal development defy the simplicity of these definitions. Firstly, not only are there numerous intermediate situations in which migrating cells exhibit a mixture of epithelial and mesenchymal characteristics, although it is apparent that these are present many more times *in vivo* than heretofore assumed (Nakaya and Sheng, 2008; Shook and Keller, 2003). Secondly, it is clearly evident now that mesenchymal cells migrate frequently, demonstrating the level of coordination and cooperation that is attributed to collectively migrating cells (Scarpa and Mayor, 2016; Theveneau and Mayor, 2011). To account for these general observations, the distinction between individual and collective cell migration has progressed away from highly rigorous to more comprehensive or loose definitions (Rørth, 2012; Theveneau and Mayor, 2011). In line with this, any effort to categorize migration events and thus derive parallels between various systems presently demands the application of strict definitions with a large number of exclusions or the stamping of new and not very well defined notions such as that the cells migrate together one at a time (Friedl and Gilmour, 2009; Rørth, 2009) or cells perform a pseudo EMT (Pastor-Pareja et al., 2004) or partial EMT (Figure 4) (Tripathi et al., 2020).

Therefore, a different perspective can be proposed. It can be hypothesized that collective and individual as well as epithelial and mesenchymal cells have distinct and independent properties that come together to divergent degrees, not only at distinct migratory events but even at various time points within a single migratory effort. Correspondingly, it is suggested that *in vivo* cell migration phenotypes cannot be divided into distinct and mutually exclusive morphological classes, but rather should be considered as a general continuum of morphological diversity that can be accomplished by a combination of different and complementary mechanisms (Campbell and Casanova, 2016).

Cancer cells can themselves alter their viscoelastic properties to counter the microenvironment through adaptation of adherens junctions throughout collective invasion. To perform this adaption, cancer cells need to employ multiple strategies, including the phenomenon that they can switch between two morphological phenotypes, such as epithelial shape and mesenchymal shape or even display intermediate forms in the course of breast cancer invasion. Moreover, cancer cells are hypothesized to intrinsically regulate viscoelastic characteristics in order to modify their cell state transition rate and efficacy of cell movement. However, there is quite a lot more research required to confirm this hypothesis experimentally or refine it theoretically through the development of models.

In specific, these cancer cells are transformed through EMT in a metalloprotease-dependent fashion and in the next step the transcription factors contribute to the transition of cadherins to further restrengthen EMT. Firstly, secreted MMP-3 metalloprotease cuts the extracellular domain of E-cadherin that lowers the adhesion strength between cell-cell junctions. Secondly, migration of the cells is based on the activation of Rac and the EMT transcription repressor Snail that lowers additionally the amount of E-cadherin in the cells (Radisky et al., 2005). This facilitates collective migration in liquid mode with fewer stringent cell-cell contacts typically conveyed through N-cadherin or L1CAM (Yano et al., 2004; Gavert et al., 2007). EMT is reversible in cancer cells, although MET has been suggested to assist mesenchymal circulating cancer cells in the development of secondary tumors (Kim et al., 2017; Li et al., 2010). Moreover, impairment of the EMT-driven transcription factors Prrx1 (Ocaña et al., 2012) or Twist (Tsai et al., 2012) may result in the activation of the MET switch. In this regard, MET endows cancer cells with robust adhesion that permits them to cease migration, aggregate, proliferate, and grow more aggressive.

In this perspective, it seems evident that although single-cell migration demands a peak of fluidity and a minority of cell-cell adhesion strength, collective migration proceeds in an optimal condition in which cell clusters can migrate either as monolayers of epithelial cells or as highly dynamic mesenchymal clusters. These optimal settings are a function of the microenvironment and come about when cells achieve the proper equilibrium of cell-cell adhesions and fluidity in a machinery imparted by a customizable EMT regimen. *In vivo*, the strength of cell-cell adhesion is not invariably adequate to sustain the collectivity of mesenchymal migratory clusters, and complementary mechanisms aid cells to migrate in a collective manner. Mutual attraction and restriction have been found to retain collectivity throughout collective mesenchymal migration out of the neural crest. In neural crest cells, mutual recruitment is generated through chemotaxis toward C3a released from the neural crest, which simultaneously expose the receptor C3aR (Carmona-Fontaine et al., 2011). In contrast, entrapment is imparted at least by the proteoglycan versican (Szabó et al., 2016). The concert of matching cell-cell adhesion with other environment-related and endogenous determinants permits cells to reduce their level of cell-cell adhesion to a baseline level to achieve fluidity and to migrate over exceptionally harsh constraints while maintaining collectivity. The following describes the viscoelasticity in multicellular processes, such as collective cell migration.

## 7 Viscoelasticity in Multicellular Processes

### 7.1 Collective Migration

Investigations employing *in vitro* and *in vivo* systems revealed that collectively migrating cells are able to utilize type-I cadherins in their adherens junctions to move within a collective mode through epithelia (Nelson et al., 2006; Wolf et al., 2007; Ewald et al., 2008; Czirók et al., 2013; Shamir and Ewald, 2015). It has been seen that collective migration is also feasible for mesenchymal cells. For example, N-cadherin-facilitated adherens junctions, permit neural crest cells inside clusters to assembly transient and flexible adhesions to migrate at an accelerated rate of adjacent interplay and fluidity, whereby the collective mode is maintained (Theveneau and Mayor, 2012; Theveneau and Mayor, 2013).

#### 7.1.1 Viscoelasticity Drives Cell Migration and Invasion

Random cell migration pertains to the intrinsic capacity of cells to migrate, often referred to as cell motility. Random walks can be ubiquitous be identified in biology (Berg, 1984). In specific, the movement of cells without symmetry-breaking gradients has long been characterized using random walk statistics (Wu et al., 2014). This basal random cell migration state opposes directional cell migration, in which cells migrate in the general direction of a chemical or physical signal. Contrary to Brownian particles, randomly moving cells display directional persistence, which means that they are far more probable to maintain motion in the previously adopted direction than to alter it, even when this direction is picked at random in an isotropic setting. The directed cellular migration can be analyzed by advection diffusion theory (Simpson et al., 2017).

*In vivo*, cancer cells are confronted with directional signals that are both soluble, such as gradients of growth factors, and non-soluble, such as the orientation of collagen fibers. Based on the number and strength of such signals, the cells migrate randomly or in a targeted manner. Moreover, these mechanical signals may also trigger the functions of cells, such as proliferation (Esmaeili Pourfarhangi et al., 2018). In this context, it is ambiguous whether cell cycle progression impacts cancer cell migration and whether this impact is distinct in random or in directional migration. Directional migration is referred to as alignotaxis, topotaxis or contact guidance, which are used synonymously (Esmaeili Pourfarhangi et al., 2018).

3D migration is not random. Both 2D and 3D migration exhibit a non-Gaussian exponential distribution of mean cell velocity, mainly due to cell-to-cell fluctuations (Wu et al., 2014). In contrast to the 2D scenario, 3D cell migration is inherently anisotropic: speed profiles exhibit varying rates and self-correlation processes in multiple directions, so that the classical persistent random walk (PRW) model of cell migration is insufficient. The inclusion of cell heterogeneity and local anisotropy in the PRW model can forecast 3D cell motility across a large range of matrix densities, thereby identifying density-independent nascent migration characteristics. This analysis also illustrates the unanticipated rugged link between cell speed and persistence of migration across a broad array of matrix densities (Wu et al., 2014).

**FIGURE 5:**
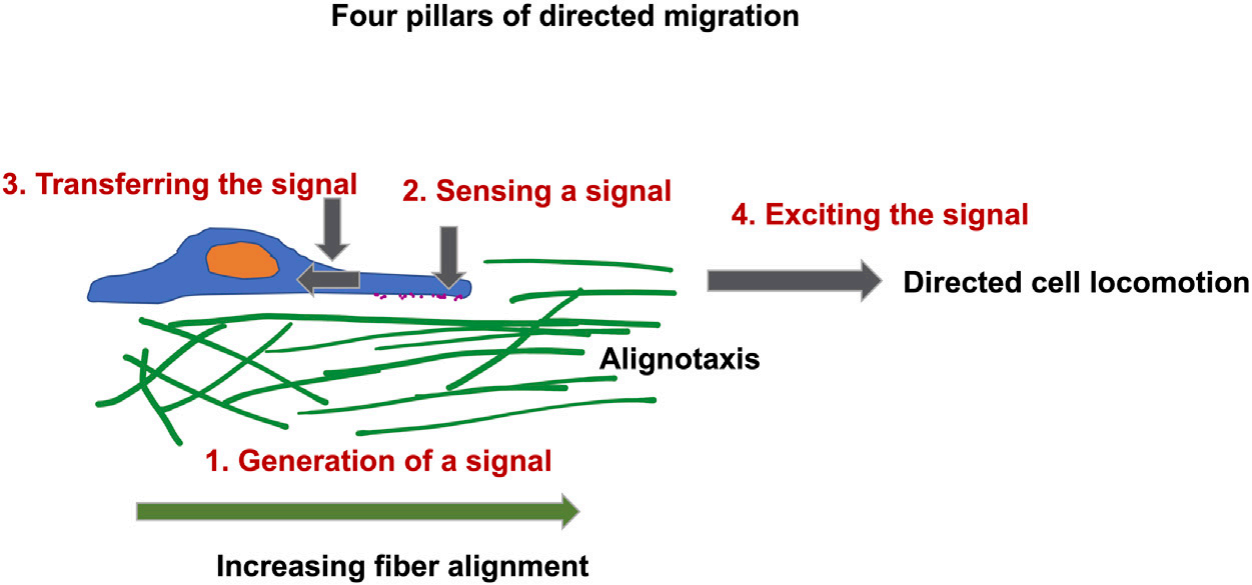
Four pillars of directed migration of cells.

Different types of migration-inducing cues lead to different migration modalities. However, during all types of directed migration the same four pillars of directed migration can be found (Figure 5). In specific detail, directed migration of cells has been found to be governed by a range of external incentives, varying from gradients of soluble (known as chemotaxis) to bound (known as haptotaxis) molecules. In complement to molecular gradients, mechanical property gradients (duro-/mechanotaxis), electric field gradients (electro-/galvanotaxis), and ambient topology pattern distortions (ratchetaxis) have also been found to be capable of directing cell migration. Because cells migrating *in vivo* are subjected to a more challenging milieu consisting of a convoluted set of biochemical, biophysical, and topological hints, it is highly improbable that cell migration is governed by a single type of gradients. As many molecular actors are implicated in the cellular answer to these affecting signals, they are frequently been recycled and act as sensors or transmitters of both biochemical and biophysical events. Xenopus neural crest cells are introduced and compared with the performance of other cell types to argue about the significance of organizing cell guidance mechanisms into different categories. In addition, it is highlighted that although the examination of single affecting cues is instructive, the real harsh reality is that cells migrate through a kind of “mixotaxis” in which they incorporate and orchestrate multiple interventions through common molecular effectors to assure the resilience of the directed cell locomotion (Barriga and Theveneau, 2020). Is the concept of mixotaxis universally applicable to all cell types?

Identifying a way to induce directional cell migration is straightforward. An external signal that the cells can translate needs to be physically arranged. Cells can thereafter employ this input to create a forward-backward polarity that permits directional locomotion following this input. There are multiple signs, including chemical, electrical, mechanical, and topological, that can be ascribed to accomplish this task in supervised and oversimplified experiments (Capuana et al., 2020; Zhao et al., 2006; Zhu et al., 2020). Since living systems are not designed to accurately follow distinct specifications in a timed manner that are controlled through by external checkpoints, under *in vivo* conditions, migrating cells are generally subjected to multiple inputs that are not hierarchically engineered and may even counteract to one another. Nevertheless, the migratory behavioral reaction of cells to such convoluted surroundings is yet consistent with logic. Moreover, any polarity evidence produced might not be as orderly orchestrated as it is in an *in vitro* array. In addition, certain cells can exhibit specific migratory patterns whereas their adjacent tissues may not. Therefore, collaboration, orchestration, and/or rivalry may arise between directionally migrating cells and the operations of their adjacent cells. Moreover, the same piece of input can lead to differing answers in diverse cell populations within the selfsame time frame, indicating that directional cues are not borne by the input itself but are instead produced as a consequence of the interference between cells and a particular input signal or series of signals. Thus, this can be analogized to how geneticists consider phenotype to be a consequence of the interactions between a genotype and an organism’s surrounding local environment. For cells to be prepared to engage in directed migration, it depends on two simple facts: First, cells need to drive themselves, and second, they require the establishment and maintenance of forward-backward polarity. In other words, all inputs need to be slightly incorporated by a cell for directional performance to result. Additionally, in clusters of cells, intercellular exchange can result in new characteristics, such a cluster of cells may behave differently than a solitary cell in a similar setting (Theveneau et al., 2010). Therefore, uncovering the mechanisms that govern directed cell migration in all its intricacy may have myriad ramifications for how to understand the complicated morphogenetic processes at play. Beyond that, a focus on a more integrative model of directed cell migration approach could aid in developing efficient routes to prevent cancer metastasis, enhance wound healing, or assist in new techniques for *ex vivo* organ sampling in the framework of regenerative medicine.

#### 7.1.2 Role of Cell-Cell-Junctions in Collective Migration Mode

Viscoelasticity can be tailored during collective migration of cells, whereby the adherens junctions play a key role. Intracellular pathway signaling activities are generally heterogeneous across the community of cells. The signal transduction process of population cells, normally random, is orchestrated through matching the fluctuating external inputs to their natural frequency (Tay et al., 2010) or through cell-cell exchange to achieve synchronization of their answers (Sun et al., 2012). For instance, collective calcium reactions in a densely packed cell population have been shown to occur when cells engage in gap junction communication in response to ATP pacing, resulting in more rapid, more synchronized, and highly corralled reactions relative to individual cells (Sun et al., 2012).

Inside the crowded cellular settings of biological tissues, the local tissue structure (Box et al., 2019) and the locomotion and deformation dynamics of single cells (Pan et al., 2016) produce propagating physical events that influence intracellular signaling pathways. The physical nature of the cellular microenvironment impacts long-term cellular behaviors related to cell fate, encompassing the polymerization of cells (Asnacios and Hamant, 2012; Copos and Mogilner, 2020), cell division (Bosveld et al., 2012; Gudipaty et al., 2017), and cell differentiation (He et al., 2018). However, the regulatory implications of mechanical stimuli on short-term cellular behaviors, including immune reactions, have not yet been elucidated extensively. Abnormal stiffness of the extracellular matrix has been demonstrated to elicit intracellular architectural alterations at the cytoskeleton-membrane interface, which in turn inhibits transient signaling replies to target-specific pharmaceuticals (Frangogiannis, 2016). The fibroblast-collagen matrix can release soluble cytokines that engage intracellular signaling transduction pathways when subjected to external mechanical force (Wells and Discher, 2008). These findings imply that dynamic mechanical inputs that arise from intercellular communications can drive intracellular responses. How mechanical signals aid cell adjustment to the constantly evolving chemical milieu and the relationship between collective cell performance and optimal tissue performance are still unclear.

Recently, this hypothesis has been explored. At a given frequency of addition of specific drugs to individual cells, chemical perturbation is converted into dynamic intracellular mechanical signals through RAC1-facilitated induction of dynamic cell-cell junctions and cytoskeletal rearrangements that act in synergy with chemical cues to favor collective signaling behaviors (Che et al., 2021). Perhaps the reverse is also conceivable: When the mechanical dynamics of the environment, including viscoelasticity, are altered, the cells may be able to react.

Beyond the hypothesized impact of intracellular structures to the overall viscoelastic character of cell, cell cluster and tissues, the cell-cell junctions and their coordination during collective cell migration may fulfill another crucial task. Emerging advances are broadening and expanding knowledge of the impact of cellular viscoelasticity throughout individual cell migration and how these characteristics can be adapted by cells to travel actively in restricted volumes. However, during collective cell migration, such mechanisms must be aligned not merely to confer the same directionality and velocity of migration to cells within the cluster, but also to permit the cluster to alter its viscoelasticity and meet the physical constraints of the microenvironment as a supracellular entity. To orchestrate these operations, cells within a migrating cluster have to link mechanically and build up efficient transmission channels to pass key messages from their surrounding environment across the cluster (Etienne-Manneville, 2011; Inaki et al., 2012). Cell-cell junctions are central in brokering these activities, and the fine-tuning of cell-cell adhesion strength has been linked to the fluidity of collectively migrating cells (Kuriyama et al., 2014; Cai et al., 2016) as well as their resistance to deformation as they move through constrained microenvironments (Cai et al., 2016). Adherens junctions and gap junctions are both fundamental in performing cell-cell communication by providing mechanical linkages, signaling and diffusion of signals via the channels. Moreover, the composition of adherence junctional complexes and the linkage to cytoskeletal filaments may provide different viscoelastic characteristics of the cells. Both adherens junctions and gap junctions react to mechanical stimuli and may therefore both be involved in the viscoelastic nature of the cells, cell clusters and tissues. Myosin-dependent contractility of actin cytoskeleton contributes to the organization and reinforcement of adherence junctions at intercellular contact sites (Krendel et al., 1999; Leckband and de Rooij, 2014). The activity of gap junctions builds a specific element of the adhesion complex, the adhesome, that couples the intercellular forces in the course of a collective migration of cells (Bazellières et al., 2015) and connexins, which represent the key elements of gap junctions and are known to act as mechanosensitive molecules (Bao et al., 2004) and may also contribute to viscoelasticity. Even though cells can be mechanically and molecularly connected through adherens junctions, gap junctions, and tight junctions (Tsukita et al., 2001; Kishikawa et al., 2008), the focus below lies on the most prominent adherens junctions as drivers of cell coupling and viscoelasticity during collective migration.

There is growing knowledge that the preferred migration mode *in vivo* seems to be the collective migration mode of cells. In collective migration, cells employ adherens junctions to mechanically link one another, which is critical for intercellular signaling processes that govern collective performance and possibly contribute to viscoelastic properties of cells. Adherens junctions perform a key function in facilitating the mechanical feedback loop within migrating cell clusters and their local environment via the coupling of force perception and transmission within the cluster and the transfer of tractions to the migrating substrate (Cavey and Lecuit, 2009; Harris et al., 2014; Kishikawa et al., 2008; Sluysmans et al., 2017). Hence, clusters may utilize various techniques to adapt their viscoelastic characteristics through precisely regulating their adherens junctions when migrating in their natural microenvironments toward altered microenvironmental mechanical cues.

#### 7.1.3 Fine Tuning of Cellular Viscoelasticity by Intracellular Factors

The nature of collective migration of cells has been seen to rely to varying degrees on the density (Gudipaty et al., 2017; Tlili et al., 2018) and motility (Bi et al., 2016; Hayer et al., 2016) of cells and intercellular adhesion (Benjamin et al., 2010; Vedula et al., 2012). For instance, in a confluent cell monolayer, an augmentation of cell motility can lead to a transition from solid to liquid (Hayer et al., 2016; Malinverno et al., 2017), whereas a breakdown of intercellular links can result in random uncorrelated cell movements (Benjamin et al., 2010; Vedula et al., 2012).

Apart from intrinsic characteristics of cells, extrinsic signals, including geometric confinement (Camley et al., 2014; Doxzen et al., 2013; Li and Sun, 2014; Lin et al., 2017; Segerer et al., 2015; Tanner et al., 2012; Vedula et al., 2012), chemical factors (Harris et al., 2012) and electric field (Cohen et al., 2014) can additionally impact dynamic properties of cells. Migrating cells *in vivo* are frequently constrained geometrically by the surrounding environment, such as extracellular matrix or other cells. Typical cases are the invasion of cancer cells within the porous peritumoral stroma (Friedl et al., 2012) and the migration of border cells within the ovaries of *Drosophila* (Montell et al., 2012).

The main physical constraints faced by migrating cells *in vivo* are adhesion (friction), boundary, rigidity of migrating substrates, shear flow of extracellular liquids, topology and density of the ambient tissue or extracellular matrix scaffold (Charras and Sahai, 2014). To address these physical constraints of a 3D microenvironment, individual cells quickly change their viscoelasticity to recontour and “squeeze” or withstand deformation (Mueller et al., 2017; Petrie and Yamada, 2012). Nonetheless, to modify their viscoelastic characteristics and face their physical migratory microenvironment as a supracellular entity, cells within migratory aggregates must orchestrate the machinery that accomplishes such transformations.

How can mechanical signals modify cell-cell adhesions? The closest and most direct interplay between mechanical force and Notch signaling takes place during the receptor activation phase, which is a sequence of cleavage events to liberate the NICD from the membrane. During this event, a concealed peptidase binding site in the extracellular negative regulatory domain of Notch is uncovered through a conformational unfolding process step (Gordon et al., 2015; Chowdhury et al., 2016; Morsut et al., 2016). Unfolding is triggered through a traction force exerted on the receptor, which is imparted through ligand endocytosis following receptor binding. This endocytic traction force is reliant on dynamin, epsin, and actin (Park et al., 2016; Seugnet et al., 1997). Epsin directs ubiquitylated ligands for clathrin-facilitated endocytosis and participates in bending of the plasma membrane (Langridge and Struhl, 2017). Dynamin creates a helical multimer that provides the force to clamp the vesicle, while actin is indispensable for producing enough force to propel endocytosis of the ligand-receptor group (Meloty-Kapella et al., 2012). The capacity of the ligand-receptor pair to resist traction relies on its molecular affinity and load-carrying capability. Curiously, the Notch ligands jagged 1 (Jag1) and delta-like ligand 4 (Dll4) exhibit distinct mechanical characteristics. The attachment of Jag1 and Dll4 to Notch1 results in catch binding, which is a type of binding that strengthens when placed under tension. The tension necessary for the activation of Notch is distinct for the two ligands, such as 4 pN for Dll4 and 12 pN for Jag1, and it has been speculated that this is attributable to an enhanced binding rate of Dll4 (Luca et al., 2017). These two ligands possess different functions, including angiogenesis, inner ear development and differentiation of the airway (Benedito et al., 2009; Petrovic et al., 2014; Stupnikov et al., 2019). The differential force magnitudes needed for Notch activation thus might enable discrimination between ligands.

Is there a direct interplay between cytoskeletal or contractile force, the activation of Notch receptors and finally cell locomotion? There exists a direct interplay between cytoskeletal or contractile forces and Notch activation. Blocking nonmuscle myosin II to decrease contractility in signal transducing cells diminishes the activation of Notch *in vivo* (Hunter et al., 2019). This decline is irrespective of the appearance of ligands or receptors on the cell surface and summative with the action of dynamin or epsin blocking, suggesting separate inputs of contractile and endocytic forces (Hunter et al., 2019).

Like intracellular forces, external forces also seem to exert an activating impact on Notch. Specifically, the timing of streaming-induced activation of Notch indicates a direct involvement of Notch in the perception of shear forces, as NICD levels rise as soon as 30 or 60 min after the initiation of shear forces (Masumura et al., 2009; Fang et al., 2017). However, this fast initiation may be inhibited through vascular endothelial growth factor (VEGF) receptor 2 (VEGFR2, synonymously referred to as KDR) blockade, which may reflect the engagement of a mechanosensitive complex and also a canonical VEGF-driven downstream signal transduction pathway (Lawson et al., 2002; Masumura et al., 2009; Coon et al., 2015). Rapid activation, nevertheless, continues to enable transcriptional to posttranslational control point regulation of Notch ligands (Shimojo et al., 2008). Indeed, the mechanoresponsiveness of the Notch transmembrane domain has been shown to be restrained by knockout of Dll4 or hindrance of endocytosis, implying that regulation of Dll4 could govern this mechanoresponsiveness (Polacheck et al., 2017). Evidence suggests that the mechanosensitive channel Piezo1 may coordinate the activity of Ca^2+^-sensitive ADAM10 in interfering with the cleavage-driven activation of Notch through Ca^2+^ ions, but decoupling this enhanced S2 cleavage from enhanced ligand generation is difficult (Caolo et al., 2020). Because of these reasons, the evidentiary support for direct notch activation through shear stress or strain is still inconsistent. A lot of interferences between Notch and mechanosensors are indirect effects. It involves the mechanoregulation of ligand or receptor synthesis and the interaction between Notch and mechanosensors.

### 7.2 Spheroid and Tumeroid Biology and Transition of Cell Collections

Realignments are frequently characterized as either passive, occasioned from an external stress on the tissue scale, or active, for instance initiated and governed through an anisotropic dispersion of molecules, including myosin or cadherins, at cell-cell contacts (Guirao and Bellaïche, 2017). Active cell outline fluctuations are the pivotal components for fluidizing tissues to flow at the tissue scale when under stress, in *in vitro* (Marmottant et al., 2009) and *in vivo* settings (Mongera et al., 2018). Experiments on embryonic tissues (Serwane et al., 2017), multicellular spheroids (Marmottant et al., 2009; Guevorkian et al., 2010), or cell monolayers in the presense or absence of an underlying substrate (Harris et al., 2012; Vincent et al., 2015) proposed that tissues can be characterized as viscoelastic fluids. Nevertheless, there is a continuing dispute about the microscopic source and magnitude of the viscoelastic relaxation time *τ* and whether Madin-Darby dog kidney monolayers act primarily as liquids or solids (Vincent et al., 2015; Tlili et al., 2018).

In qualitative terms, flow modeling revealed that the cell deformation and rearrangement rate fields are strongly related in the absence of cell division and apoptosis (application of the pharmacological drug mitomycin). This is consistent with a simple Maxwell viscoelastic fluid model in which an elastic intracellular constituent is connected in sequence with a viscous intercellular constituent representing cell redistributions (Tlili et al., 2015). The overall deformation rate represents the strain rate, otherwise known as the velocity gradient. Specifically, this is the total of the coarse-grained cell deformation rate and the intercellular topological alteration rate, which is termed rearrangement rate. The change in relaxation behavior potentially provides a pathway to gradually shift from a developing, fluid tissue to a mature, firm and solid tissue (Mongera et al., 2018). Encoding memory of overall tissue flux in the cellular form is a conduit for transmitting knowledge from the tissue to the cellular level and, conversely, may affect intracellular signal transduction (Jain et al., 2013).

#### 7.2.1 Spheroid and Tumeroid Biology

Commonly employed culture models include spheroids, which are well-formed aggregates of cells. As cancer model systems, tumor spheroids are capable of accurately replicating the core characteristics of solid human cancers. Spheroids and organoids are both 3D structures consisting of multiple cells. Although these terms are synonymously referred to, there are clear dissimilarities between them. Organoids comprise 3D stem cell models derived from either embryonic or adult stem cells. This kind of organoid can display self-regulatory capability, phenotypic characteristics of the organ from which they are cultured, and ambient physiological modeling via genomic changes. An organoid is a compilation of organ-specific cell types derived from stem cells or organ precursors that self-organize similarly to *in vivo* through cell sorting and spatially constrained lineage commitment (Lancaster and Knoblich, 2014). However, the multicellular tumor spheroid model has been first introduced in 1971 and acquired by culturing cancer cell lines in nonadherent environments (Sutherland et al., 1971). Tumor spheroids are a cancer stem cell expansion model; tissue-derived tumor spheroids and organotypic multicellular spheroids are generally harvested through mechanical disassembly and sectioning of tumor tissue (Weiswald et al., 2015). In principle, higher-order self-organization occurs in organoids than in spheroid cultures, and the first are more strongly reliant on a matrix for their emergence. In the following spheroid and tumeroid cultures have been frequently employed to analyzed and decipher the phenomenon of transitions in cell collections.

#### 7.2.2 Role of Viscoelasticity in Jamming to Unjamming Transition of Cell Collections

**FIGURE 6:**
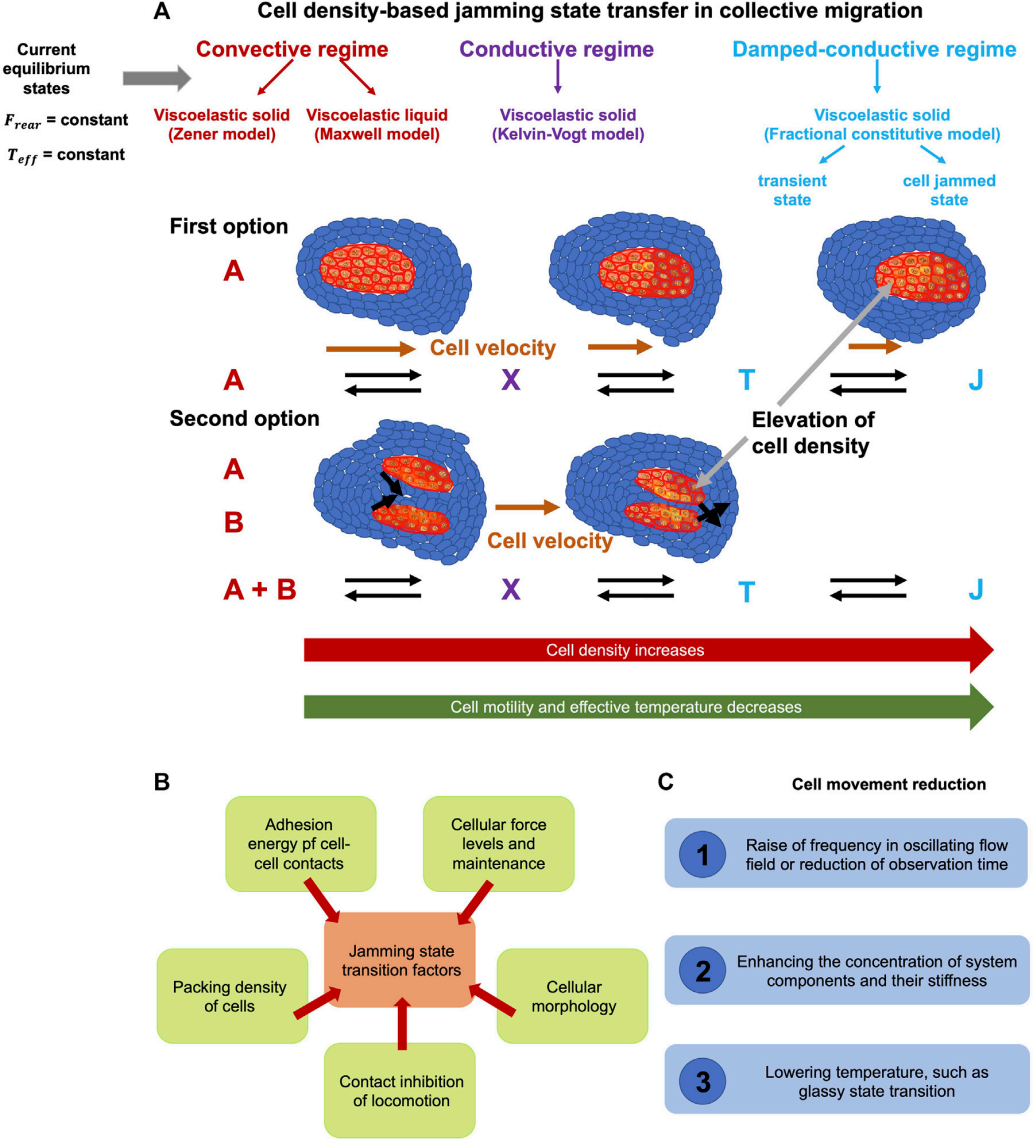
Cell-density driven jamming state transfer in collective migration of cells. Viscoelastic stages, jamming to unjamming transition and migratory capacity of cells. **(A)** There exist five viscoelastic states within three distinct physical regimes. **(B)** The jamming to unjamming transition factors. **(C)** Three classical parameters may also lead to a decrease in cell locomotion.

The viscoelasticity of multicellular arrays relies on the wandering cell constitution and the velocity of its transformation, which proceeds by the transitions from the wandering to the resting cell condition and conversely (Pajic-Lijakovic and Milivojevic, 2019a; Pajic-Lijakovic and Milivojevic, 2019b). These crossings have been regarded as cell jamming state transitions due to disturbances (Angelini et al., 2011; Bi et al., 2016; Garcia et al., 2015; Nnetu et al., 2013; Oswald et al., 2017; Park et al., 2016). Multiple interdependent elements affect the jamming phase transitions such as: firstly, an elevation in the packing density of cells (Henkes et al., 2011; Nnetu et al., 2013), secondly, the adhesion energy of cell−cell contacts (Bi et al., 2016; Garcia et al., 2015), thirdly, amount of cellular forces and maintenance of these forces (Garcia et al., 2015), fourthly, cellular morphology (Garcia et al., 2015; Park et al., 2016), fifthly, contact inhibition of locomotion (CIL) (Zimmermann et al., 2016) (Figures 6A,B). The impairment of cell motility affects the condition of viscoelasticity and, subsequently, the cell jamming phase transition. In spite of substantial research aimed at investigating cell jamming, the process is still not completely comprehended from the viewpoint of rheology. Nevertheless, the mechanism(s) whereby this jamming condition occurs are linked to the viscoelasticity (Pajic-Lijakovic and Milivojevic, 2019b).

The classical material-science modification of the state of viscoelasticity is induced by the decrease of the agitation of system elements. The motion may be limited firstly, by raising the frequency in the oscillating flow field or reducing the observation time, secondly, by enhancing the concentration of system components and their stiffness, and thirdly, by lowering the temperature, such as the glassy state transition (Pajic-Lijakovic and Milivojevic, 2021a) (Figure 6C). The apparent enhancement of the stiffness of the system components can be derived from the intensification of the field of interactions which are instantiated through the excluded volume concept (Neal et al., 1998). The rheological reaction of coacervate-based systems has been examined in the oscillating shear field at rising frequency, and four regimes have been identified. The first regime is a low-frequency terminal regime that can be described through the Maxwell model (a viscoelastic liquid). The second regime represents a middle-frequency plateau regime that can be explained through the Kelvin-Voigt model (a viscoelastic solid). The third regime represents a higher-frequency transition regime (a viscoelastic solid). The fourth is a high-frequency jamming regime (a viscoelastic solid) (Figure 2) (Liu et al., 2010; Schroyen et al., 2020). There are minimally two or perhaps even three regimes (Stamenović, 2006). The final regime is typified by intense and disordered motion of the system elements and by in-chain reciprocal interactions, which leads to substantial energy dissipation. Plateau regime is equivalent to intense entropic forces generated due to interchain dynamics (Pajic-Lijakovic and Milivojevic, 2021a). The transition regime is connected to diminished mobility of the system components and explains local conformational alterations of the chain moieties influenced strongly through the entanglement relaxation time (Liu et al., 2006). The main features of the jamming condition are: firstly, migration of the system elements is largely damped, such that the diffusion parameter approaches zero, secondly, relaxation time goes to infinity, and thirdly, storage modulus G′(ω) and loss modulus G″(ω) meet the requirement G′(ω)/G″(ω) = const > 1 (Hunter and Weeks, 2012; Pajic-Lijakovic and Milivojevic, 2019a).

The density-facilitated cell jamming state transition in the course of collective cell migration has been broadly examined (Angelini et al., 2011; Bi et al., 2016; Garcia et al., 2015; Nnetu et al., 2013; Oswald et al., 2017; Park et al., 2016). Nevertheless, the relevant variation of the condition of viscoelasticity has not been incorporated. To fill this loophole, a systematic theoretical approach from a rheological point of view is offered. Specifically, it has been seen that there occurred the density-induced switch from a convective toward a conductive mechanism of collective cell migration (Angelini et al., 2011). Consequently, this transition alters the stage of viscoelasticity. In agreement with this, the velocity correlation length has been inferred to become dependent of the cell velocity (Garcia et al., 2015). In specific, the correlation length firstly raised with cell velocity for cell velocities less than about 1 μm/min and secondly declined with cell velocity for cell velocities greater than 1 μm/min. These two tendencies of cell movement comply with distinct states of viscoelasticity. An even more advanced elevation of the packing density of cells evokes a decrease in cell mobility which materializes in subdiffuse cell migration that initiates the anomalous character of energy dissipation (Nnetu et al., 2013). Cell monolayers seem to act as amorphous solids at decreased cell speeds (Garcia et al., 2015). Each viscoelastic condition per regime ought to be typified by a suitable stress-strain constitutive model. For instance, the collective migration of cells causes the generation of stress, stress relaxation, and the buildup of remnant stress (Pajic-Lijakovic and Milivojevic, 2019b, Pajic-Lijakovic and Milivojevic, 2020). The stress level ranges from normal including compressive and tensile to shear (Pajic-Lijakovic and Milivojevic, 2019a, Pajic-Lijakovic and Milivojevic, 2020). Normal stress is concentrated mainly within a core area of migrating cell clusters amassed during their motion through the crowded surroundings (Pajic-Lijakovic and Milivojevic, 2019b; Pajic-Lijakovic and Milivojevic, 2021a) and during clashes of migrating cell faces (Nnetu et al., 2012). Normal intrinsic stress accumulation is accountable for enhancing cell packing density (Trepat et al., 2009) and lowering cell motility (Pajic-Lijakovic and Milivojevic, 2021a), and subsequently, for the transition to the stagnant jammed state (Pajic-Lijakovic and Milivojevic, 2021b). The theoretical analysis incorporates: the organization of the established regimes through cell velocities, the degree of modification of the cell packing density and viscoelasticity on a supercellular scale. In the following, the emphasis is placed on an effort to elucidate the density-driven development of viscoelasticity and the state transition of cell jamming, and to highlight the factors governing the dynamics of long-term redistribution of cells, along with their interconnections.

#### 7.2.3 Impact of Stress on Viscoelasticity

Collective cell migration encompasses stress generation, stress relaxation, and intrinsic stress build-up (Pajic-Lijakovic and Milivojevic, 2019a, Pajic-Lijakovic and Milivojevic, 2020). The allocation of normal, including compressive and tensile, and shear residual stresses has been quantified for multicellular 2D systems conducted under *in vitro* settings (Nelson et al., 2006; Serra-Picamal et al., 2012; Tambe et al., 2013). Thereby, the maximum stress is in the range of 100–150 Pa (Tambe et al., 2013). Normal residual stress build-up leads to an augmentation of the density of cell packing (Trepat et al., 2009). An elevation in cell packing density acts to trigger a decline in cell velocity (Angelini et al., 2011; Nnetu et al., 2013; Tlili et al., 2018) and, building on this, a shift in the condition of viscoelasticity.

Two specific scenarios of augmented packing density of cells can be assumed, one within migrating clusters, denoted A, caused by normal residual stress accumulation during their migration through dense environment and the other produced by colliding velocity surfaces, denoted A and B (Pajic-Lijakovic and Milivojevic, 2019b). Cell separation is disregarded on this temporal scale. Both settings can result in the switch from the migratory to the dormant and stalled cell state (Pajic-Lijakovic and Milivojevic, 2019a) (Figure 6). A and B signify distinct conditions of cell migration within a convective regime, X signifies cell migration occurring within the conductive regime, T signifies the transient condition of the cell encountered within the damped-conductive regime, and J signifies the condition of cell engorgement or jamming also experienced in the damped-conductive regime (Figure 6). Systems have to pass through a transitory mode before entering the perturbation mode, such as the jamming state (Baumgarten and Tighe, 2017). The fundamental mode of migration for the specific cell conditions marked A and B is convective, whereas the transient condition X represents cell movement through a conductive type of mechanism. Therefore, the three regimes are experimentally detected in collective migration of cell monolayers (Angelini et al., 2011; Nnetu et al., 2013; Garcia et al., 2015). The transient and perturbative (jammed) conditions comprise portions of this damped-conductive regime. For each regime, cell velocity, which is the degree of alteration of the cell displacement field evoked through collective cell migration, packing density of cells, and the respective constitutive stress-strain relationship define the regime. Conversions from convective to conductive modes and from conductive to damped-conductive modes are initiated through normal residual stress build-up with a concomitant rise in cell packing density (Pajic-Lijakovic and Milivojevic, 2021b). However, feedback crossovers from the damped-conductive to the conductive regime and from the conductive to the convective regime are induced through contact inhibition of locomotion (Zimmermann et al., 2016; Alert and Trepat, 2020). Contact inhibition of locomotion is accountable for the disorganization of multicellular systems and the attenuation of cell-cell adhesion interaction sites (Mayor and Carmona-Fontaine, 2010). Density-driven transfers from one regime to another may be handled as viscoelastic phase transformations.

Raising the packing density of the cells lowers the effective volume per individual cell and intensifies cell-cell interactions on this way. The elevation of the cell packing density decreases the free volume, V_f_, but the individual cell volume remains nearly constant throughout the collective cell migration. The jamming condition arises when the free volume is equivalent to the exclusive volume. This requirement reinforces the contact inhibition of locomotion, which can induce decongestion or unjamming (Alert and Trepat, 2020). The principle of excluded volume has been established in physics to characterize multibody interactions in a variety of soft systems of polymers and soft and stiff particles (Neal et al., 1998). The excluded volume is related to the interaction field and has been formulated from the second virial coefficient (Neal et al., 1998). This principle has been extended to dense cell populations to specify the minimum effective volume per individual cell equivalent to the jamming condition and, building on this, the peak cell packing density.

Significant efforts have been devoted to debating the density-driven condition transformation of the cell jamming phenomenon occurring throughout the collective 2D cell migration. Cell jamming is a corollary of the decrease in cell motility induced due to normal residual stress build-up. Inherent stress accumulation impacts firstly, packing density of cells, secondly, cell-cell adhesion energy, thirdly, magnitude of cellular forces, fourthly, shape of cells, fifthly, contact inhibition of locomotion, and sixthly intricate interrelationships. The impairment in cell mobility affects the stage of viscoelasticity. However, the jamming phase switch is elusive from a rheological point-of-view.

Moreover, the gap needs to be filled through the clarification of the linkage between either viscoelasticity and packing density of cells or viscoelasticity and migratory capacity of cells. This density-driven development of viscoelasticity keys in accounting for multiple crossovers among five viscoelastic modes obtained in three regimes: firstly, convective regime, secondly, conductive regime, and thirdly, damped-conductive regime. The convective regime contemplates two conditions of viscoelasticity in function of the amount of cell speed and the degree of cell-cell adhesion interactions. Increased cell velocities and faint cell-cell adhesion interactions (indicative of inferior cell packing efficiencies) yield distinct fluid-like characteristics delineated by the Maxwell model, whereas slower cell speeds and firmer cell-cell adhesion interactions (indicative of confluent multicellular regimes) assure solid-like characteristics delineated by the Zener model. The switch from convective to conductive mode is paralleled by a marked decrease in cell migration induced by the rise in normal inherent stress build-up. It is this phenomenon, referred to as the “plateau” mode in the vicinity of the system perturbation, such as cell jamming, that has been delineated through the Kelvin-Voigt model. A continued enhancement of cell packing tightness results in an anomalous character of energy dissipation throughout the collective cell migration as a feature of the damped-conducting mode regime. This regime incorporates two states of viscoelasticity, namely the cell transient condition and the jamming condition characterized through the fractional constitutive model. The process of viscoelasticity development is implemented by multiple current equilibrium conditions. Each equilibrium condition has been thermodynamically defined in terms of constant factors: on the one hand the Helmholtz free energy of cell restructuring and on the other hand, the effective temperature. The density-induced decrease in cell motility disrupts the equilibrium condition by lowering the effective temperature. The subsequent equilibrium condition accompanied by a new condition of viscoelasticity is determined for the new levels of effective temperature and Helmholtz free energy of restructuring events. Complementary experiments are needed to firstly relate the normal residual stress accretion to the variation of cell packing density, secondly reveal the cell number density and damping coefficient for the jamming condition of diverse cell types, thirdly identify the appropriate ingredients for the suggested constitutive models, fourthly outline the time evolution of the viscoelastic modification from the convective regime to the damped-conductive regime within several 2D and 3D experimental settings, and fifthly locate the required time span for the cells to pass through the unjamming transformation.

## 8 Viscoelasticity of Cells Varies due to Disease State and Progression

There is agreement that there exist differences in viscoelastic characteristics of cells in the various diseases, such as cancer, fibrosis and inflammation. It seems to be likely that there are also pronounced differences of disease models, when the cells are cultured in 2D vs 3D. The viscoelastic value of cells is no static number, it is rather dependent on time and is hence dynamically regulated. It is rather suggested to correlate with the progression of the disease, including the malignant progression of cancer. Moreover, the viscoelastic response can also be implemented, when investigating the reaction toward specific drug treatments, since it has been shown that 2D cell culture-based drug screenings turned out to be ineffective in predicting whether a drug treatment helps to treat cancer and its malignant progression. Even the chemo- or radiotherapy approaches cannot be predicted due to altered tumor microenvironment and the broad heterogeneity of cancer types.

### 8.1 Viscoelasticity in Cancer

**FIGURE 7:**
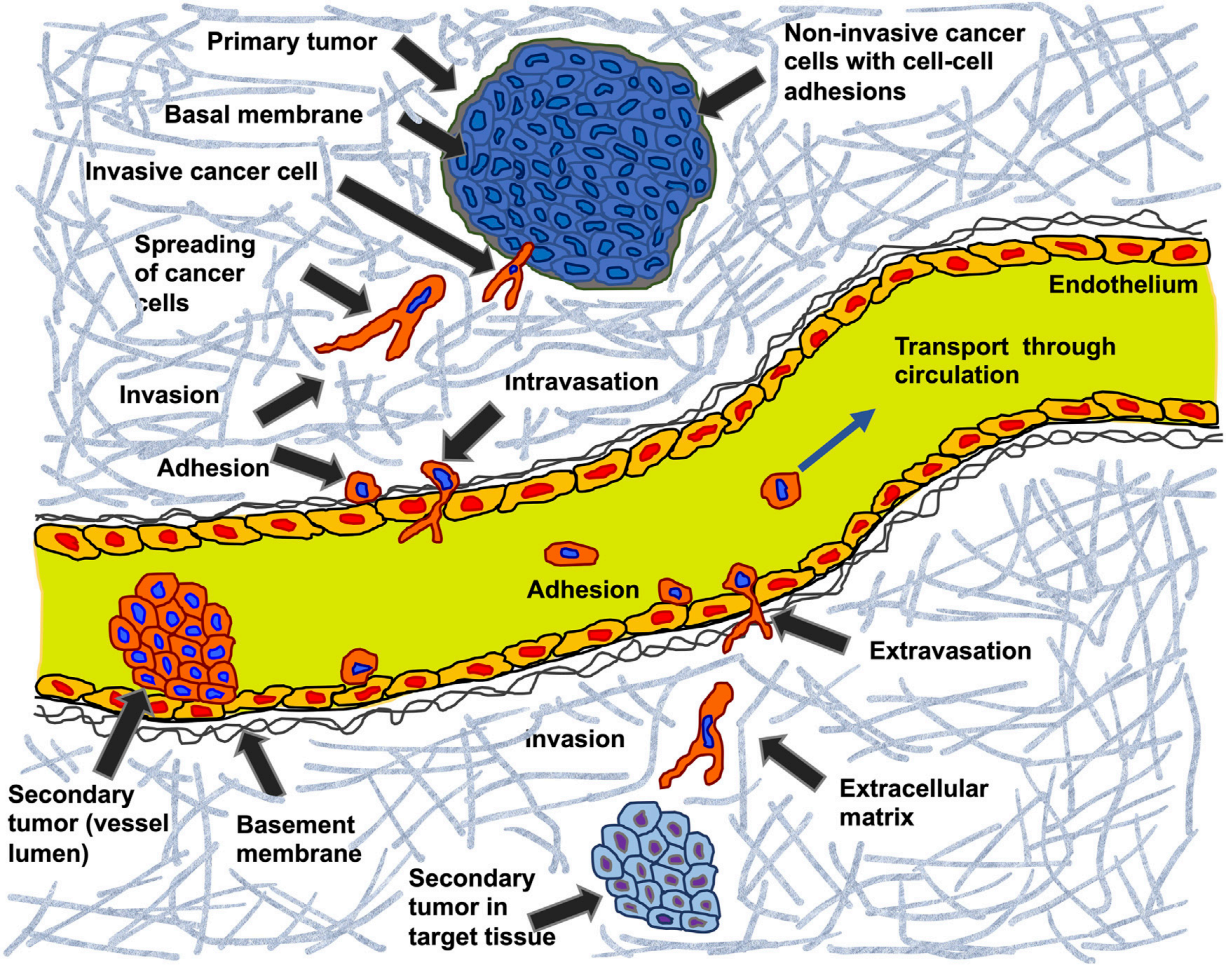
Cancer metastasis is based on an accurately regulated and sequential order of events.

The transition of cancer cells from a benign phenotype to an invasive or metastatic identity entails both biological features, such as upregulation, downregulation, or inhibition of the expression of specific genes and tumor markers (Simpson et al., 2005), and physical features, such as structural alterations of cells and tissues that cover molecular and scaffold structures (Hall, 2009; The Physical Sciences - Oncology Centers Network, 2013; Wirtz et al., 2011). In the last two decades, mechanical modifications of the phenotype of cancer cells have emerged as an integral piece of what has been seen as role of focus in cancer disease progression (Kumar and Weaver, 2009; Mierke, 2014, Mierke, 2019). From the liberation of cells from a primary solid tumor into the bloodstream via intravasation, as circulating cancer cells to their extravasation into surrounding tissues and their settlement in a new targeted organ, there are pronounced changes in the mechanical characteristics of the cells, particularly their adhesion and inherent rheology (Figure 7) (Kumar and Weaver, 2009). Comprehending cancer from the viewpoint of biomechanics may offer an alternative avenue to assess the outbreak or advancement of the disease. For instance, the rise in cell deformability corresponds in a direct way to the development of a transformed phenotype from a non-tumorigenic, benign cell towards a tumorigenic, malignant cell. The reduction in the amount of actin in the cytoskeleton and its overall structure is linked directly to the modifications in the biomechanical characteristics of the cells (Ketene et al., 2012). However, elasticity by itself is not typical of the entire mechanical nature of the cell. Cells exhibit viscoelasticity, i.e., they react in a time-dependent manner to an imposed force, which is due to interior frictional interfaces between the cell constituents and the organelles. In a 3D environment there are additional frictional interfaces between the cells and their microenvironment, which also has frictional interfaces within its own scaffold. Hence, it is expected that the viscoelastic characteristics of cells are altered by a 3D matrix scaffold. In line with this, when a 3D matrix is additionally cross-linked which leads to bundling of fiber and less friction, the matrix viscoelasticity is reduced and the material is more elastic which in turn increases the motility of cancer cells. The viscoelastic character of the cells renders them heavily reliant on the rate of deformation. The elastic modulus of breast cancer cell lines appeared to be governed by the loading rate during indentation (Li et al., 2008). However, to date, there have been several investigations on the viscosity of cancer cells using AFM. Analyses of cancer cell viscoelasticity can also provide a baseline for drug screening and therapy, since drug diffusion into cancer cells is partially a function of cell viscosity as medication molecules navigate their passage through the viscous cytoplasm (Kalwarczyk et al., 2011). A recent experimental study indicates that metastatic cancer cells rigidify upon active invasion and are completely imbedded in collagen inside a 3D environment (Staunton et al., 2016). This demonstrates the critical importance of determining the viscoelasticity of cancer cells in their suspended form while in the circulation of blood vessels during the invasion event. These findings suggest that the elastic modulus decreases significantly as the metastatic capacity of a cell increases from control human breast MCF-10A epithelial cells to non-invasive human breast MCF-7 cancer cells to highly invasive human breast MDA-MB-231 cancer cells (Fischer et al., 2017; Nematbakhsh et al., 2017). It implies that breast cancer cells soften as they progressively increase in invasiveness. Moreover, highly invasive breast MDA-MB-231 cancer cells can exert higher forces, as indicated by the absolute fiber displacement of 3D collagen fiber networks, compared to non-invasive breast MCF-7 cancer cells or control breast MCF-10A mammalian epithelial cells (Fischer et al., 2017).

Besides the investigation of single cells, cell spheroids seem to provide specific features that may help to understand what is happing in a solid tumor in more detail. For instance, spheroids can actually outgrow and produce their intrinsic extracellular matrix, and cell adhesion molecules, notably cadherins, such as E-cadherin, can arise to tether cells inside the spheroid. Several prior research efforts have centered on the specific impact of compressive stresses applied externally to the solid tumor (Helmlinger et al., 1997; Delarue et al., 2014), which had only a minor impact on spheroid growth. Fewer findings are available to support comprehension of the mechanical response of such spheroids, however recent modeling has centered on the flow of interstitial fluid inside the spheroids (Delarue et al., 2013) and has demonstrated that poroelastic active models can completely characterize them (Dolega et al., 2021). When low quantities of collagen (in this case between 0 and 0.03 mg/ml) have been utilized, cells have been observed to reorganize themselves effectively to create a more condensed structure (Abidine et al., 2021), where collagen has been employed as a surrogate (Delarue et al., 2013; Elosegui-Artola, 2021). This led to several responses that can be attributed to the viscoelastic character of collagen (Vader et al., 2009; Iordan et al., 2010; Aermes et al., 2020). In fact, collagen has improved viscous characteristics at low frequencies and presents extremely small moduli that are typically a few tens or hundreds of Pa (Aermes et al., 2020).

How can poroelasticity and viscoelasticity be distinguished from each other? Poroelastic and intrinsic viscoelastic effects are both energy dissipation mechanisms, whereas the poroelasticity depends on the flow and viscoelasticity is flow independent. Poroelasticity has been demonstrated in connective tissues such as articular cartilage, which is comprised of a load-bearing and dissipative element over a wide-band spectrum of loading frequencies. Thus, cartilage has a heterogeneous structure consisting of a dense solid matrix with collagen fibrils and proteoglycans and a fluid (Mow et al., 1992). Here, the liquid is the largest component (approximately 60–85% of the wet weight) and is critical in swelling the interfibrillar cavity (approximately 30% of the overall water) and the extrafibrillar cavity (Torzilli, 1985; Maroudas et al., 1991; Mow et al., 1992). Cartilage dehydrates and rehydrates as a result of pressure-induced exudation of liquid through the solid matrix at ordinary loading rates *in vivo*. The time-dependent characteristics of cartilage are based on paired mechanisms involving the solid matrix and fluid flow. The mechanisms have been typified as poroelasticity and intrinsic viscoelasticity, leading to effective and persistent broadspectrum dissipative characteristics (Fulcher et al., 2009; Lawless et al., 2017; Nia et al., 2011, Nia et al., 2013).

Prior investigations have yielded indications of poroelasticity and intrinsic viscoelasticity of cartilage, although the respective relational contributions of the two remain ambiguous. The dissipation and response due to poroelasticity is based on the frictional interaction between the solid and the fluid, and hence relies on the flow (Nia et al., 2011, Nia et al., 2015). Poroelastic-driven dissipation is dominant at relatively small length scales (approximately 5–6 μm) under oscillatory loading (Nia et al., 2011; Nia et al., 2013). Intrinsic dissipation powered by viscoelasticity is connected with a retardation caused by molecular friction and reorganization of a solid matrix (Nia et al., 2011; Nia et al., 2015) and is consequently independent of flow (Mak, 1986; June et al., 2009; Lai and Hu, 2017). The intrinsic viscoelasticity of cartilage has been analyzed with macroscopic compression experiments (Mak, 1986; Fulcher et al., 2009; June et al., 2009; Lawless et al., 2017) and measured with small magnitude shear loads (Henak et al., 2016). Although a handful of investigations have assessed poroelasticity (Nia et al., 2011; Nia et al., 2013) and intrinsic viscoelasticity of cartilage (Fulcher et al., 2009; Lawless et al., 2017) individually across a broad frequency spectrum, their relative contributions have not been separated. It is further challenging to use previous results to decouple mechanisms because the test length scales of approximately 5–6 μm for poroelasticity, which is locally determined, versus about 5 mm for intrinsic viscoelasticity, which is determined across the entire thickness, are polarized. As a consequence, the depth-dependent heterogeneous structure of cartilage, encompassing collagen direction and diameter, cannot be accurately benchmarked.

Dissipation caused by poroelasticity is length dependent, whereas dissipation caused by intrinsic viscoelasticity is not. This disparity permits us to discern the respective contributions of the two. The decoupled dissipation mechanisms have shown that intrinsic viscoelastic dissipation provides persistent wide-band dissipation at all length scales, while additional poroelastic dissipation raises the overall dissipation at small length scales. In order to model the poroelastic tissues, the poroelastic constitutive model has been developed in 1956 (Biot, 1956a; Biot, 1956b) and extended to accommodate oscillatory behavior (Cheng et al., 1991; Perrinez et al., 2009). A poroelastic material is characterized as a biphasic continuum consisting of a porous elastic matrix and a penetrating pore liquid. Volumetric deformation of the solid matrix causes fluid flow inside the material. Inversely, a liquid penetrated into the material effects a deformation of the matrix scaffold.

### 8.2 Viscoelasticity in Wound-Healing

Apart from the overall tissue inflammation, viscoelasticity can also occur on smaller length scales. For example, viscoelasticity itself can act as a characteristic indicator for the activation of leukocytes (Zak et al., 2021). The elastic and viscous properties of leukocytes evolve in parallel and have a well-defined proportion when measured dynamically using a micropipette (Zak et al., 2021). Thus, viscoelasticity represents a mechanical specification that is intrinsically unique to each cell type. Using microfluidic system the flow behavior of red blood cells can be analyzed in a microcirculation-mimicking framework of PDMS microchannels that exhibit a channel width similar to cell size (Tomaiuolo et al., 2011). In a 3D microenvironment the immune cells can be embedded within the 3D matrix scaffold and survive longer without the excessive addition of growth factors. Subsequently, the viscoelastic characteristics of the immune cells are be maintained on longer time intervals and thus the wound-healing process can be modeled in a more physiological manner.

For proper tissue and wound repair, it is critical that the viscoelastic, physical, and biodegradable characteristics of the scaffolds can be precisely adjusted to optimize cell performance, such as cell attachment, proliferation, and infiltration, all of which are essential to the healing process (Sharma et al., 2016). For the latter process, the motility of the immune cells is of critical importance that relies on their capacity to transmigrate out of endothelial blood vessels and invade through the extracellular matrix toward the sites of inflammation/injury. Apart from immune cells, fibroblasts and organ-specific epithelial cells are recruited to the sites of tissue injury in order to close the wound. By release of matrix cross-linking or matrix degrading substances, these cell type can have an impact on the mechanical phenotype of matrices, which in turn alter the viscoelastic response of embedded cells, such as fibroblasts, epithelial cells and endothelial cells.

For example, reestablishment of lung homeostasis after wounding necessitates efficacious wound healing through the epithelium. Among the mechanisms of epithelial wound healing in the lung are the dissemination and migration of cells into the injured zone and thereafter cell proliferation. It can be hypothesized that the mechanical characteristics of cells vary proximal to the wound margin and therefore may yield evidence to guide cell migration (Wagh et al., 2008). In the case that the epithelium is injured, it is critical to promptly rebuild the barrier’s intactness in order to avoid additional infection or deterioration. The normal repair mechanism of airway epithelial cells, which has been examined in cell cultures (Savla et al., 1997; Desai et al., 2004), in animals (Park et al., 2006), and finally in humans (Heguy et al., 2007), comprises a coordinated cascade of sequential episodes that involve formation of a temporary matrix, dedifferentiation of proximal secretory and ciliated cells, swift attenuation and spreading of these cells, immigration into the injured region to cap the surface, and, subsequently, proliferation and differentiation of cells to restore the functionality of the epithelium (Holgate, 2004; Hackett and Knight, 2007). The extremely early phases of epithelial restoration, which include cell spreading and migration, emerge within hours of wounding, and these events require significant tremendous reshaping of the cellular structure. Moreover, the remodeling event in epithelial cells entails the concerted locomotion of a layer of cells rather than the migration of single cells.

The dynamic rearrangement that proceeds during cell spreading and migration is based on the generation of directional polarity of migrating cells and the elongation of protrusions in the path of migration (Lauffenburger and Horwitz, 1996; Ridley, 2003; Kole et al., 2005). These protrusions can be in the shape of wider lamellipodial projections or extended and slender filopodia. This is closely followed by a repetitive sequence of processes in which the protrusions build adhesions with the substrate, posterior adhesions are broken down, and contractile mechanisms draw the cell anteriorly. An essential component of these events is the tightly managed reorganization of cytoskeletal structures at distinct locations inside the cell. Actin filament remodeling, adhesion site generation and turnover, and actomyosin contraction are governed in some part by members of the Rho family of small GTPases (Van Aelst, 2002; Holgate, 2004). Therefore, it has been physically revealed previously that an unbalance in the activity of RhoA or Rac1 can interfere with normal repair mechanisms in airway epithelial cells (Desai et al., 2004). Due to its salient involvement in spatially regulated actin-myosin contraction events, which are indispensable for cells to produce the forces essential to pull ahead in cell migration, either an increase or a decrease in RhoA activity induced alterations in actin partitioning and led to substantially slower epithelial damage repair.

It is possible to alter membrane tension by changing phospholipid composition or by osmotically swelling cells (Raucher and Sheetz, 2000). They found that the lamellipodial expansion rate decreased with increasing membrane tension. They hypothesized that membrane tension levels may govern the rate of actin polymerization throughout lamellipodial expansion. Relative to cells adhering to a rigid substrate, cells adhering to softer substrates display fewer organized actin reticulations and fainter focal adhesions, and produce fewer tension (Felsenfeld et al., 1999). It has been proven that polymerization of microtubules toward the adhesion sites augments under elevated stress when force is exerted on the cell body with a microneedle or when the substrate is uniaxially stretched (Kaverina et al., 2002). It has been consistently illustrated that mechanical stretch and compression events pronouncedly reduce the motility of cells and wound sealing within airway epithelial cells (Savla et al., 1997; Savla and Waters, 1998). Yet, there is limited knowledge about how locally mechanical characteristics change throughout cell migration (Rotsch et al., 1999; Nagayama et al., 2001; Kole et al., 2005; Laurent et al., 2005). Therefore, it can be hypothesized that local mechanical characteristics alter as respiratory epithelial cells migrate, perhaps either as a cue to somehow concert the cell migration or in reaction to structural reorganizations occurring within the cells.

The central part of a migrating fibroblast initially stiffened in comparison to the central part of a stalled cell, however, then the stiffness in the central part is pronouncedly reduced in the course of cell migration (Nagayama et al., 2001). When migrating and stalled fibroblasts are compared, intracellular mechanical properties have been revealed employing microinjected fluorescent microbeads (Kole et al., 2005). In fact, after 4 h, the cells at the border of an injured cell layer are not so much deformable and hence stiffer than resting fibroblasts.

### 8.3 Viscoelasticity in Fibrosis

Viscoelasticity is a mechanical property of multiple cell types, cell spheroids, tissues and entire organs. Among the tissues, the liver and the lung have frequently been chosen as model systems, as the both organs are quite prone for inflammatory diseases, including fibrosis. Fibrosis, which is the development of scar tissue as a consequence of overly impaired wound healing, is associated with a substantial increment in the accumulation of extracellular matrix. Inflammation itself is known to be an essential driver of fibrosis. During the process of fibrosis, the motility of fibroblasts in particular is an important driving factor.

*In vivo* magnetic resonance elastography measurements have demonstrated that the prevalence of liver inflammation and fibrosis leads to increased stiffness values (Huwart et al., 2006; Sinkus et al., 2018). This alteration can also render embedded cells, such as fibroblasts more elastically and less viscoelastic, which in turn consequently elevates their migratory capacity. However, disease coexistence (Mueller, 2010) and major deformation error bias in elastography measurements (Capilnasiu et al., 2019) may confound diagnosis even more. In recent decades, a number of rheological assays have been performed to accurately define liver tissue, with uniaxial deformation (either by loading a small specimen or by indenting the entire organ) and shear being the most widely applied. Both oscillatory shear and uniaxial deformation response experiments indicate that the liver presents quasi-linearity at low strains, with nonlinear performance emerging at higher strains (Gao et al., 2010; Liu and Bilston, 2000; Tan et al., 2013). In addition, loading/unloading experiments show that hysteresis effects occur (Jordan et al., 2011), where the reaction is frequency sensitive (Liu and Bilston, 2000; Miller, 2000). Multifrequency soft tissue assessments of shear modulus G* reveal a fractional order reliance on angular frequency (Sinkus et al., 2018). Other biomechanical features of the liver have also been studied, including relaxation (Chatelin et al., 2011; Liu and Bilston, 2002) and creep (Wang et al., 1992).

Measurements of liver tissue rheology have resulted in a number of biomechanical models. Hyperelasticity is frequently implied, with polynomial, exponential, and logarithmic shapes adopted for compression and strain measurements (Chui et al., 2004; Gao et al., 2010). The general results imply that the exponential, logarithmic, and power law models provide greater versatility in covering the various ranges of stress-strain curves. To study viscoelasticity, cyclic deformation or relaxation assays generally are required. Specific investigations utilized relaxation (Liu and Bilston, 2002), shear oscillations (Nicolle et al., 2010; Nicolle et al., 2013) or cyclic indentation probing (Jordan et al., 2011) over a broad spectrum of frequencies, to obtain a more comprehensive view of the biomechanical response of the liver. Of these models, the Kaye–Bernstein-Kearsley-Zapas model has been recommended because it takes into account the entire time history and has been confirmed for small-amplitude oscillatory shear and strain gradients (Nicolle et al., 2010, Nicolle et al., 2013). Optionally, viscoelasticity could be simulated through the implementation of a Maxwell element. A sophisticated differential model with ten different model parameters has been examined in terms of relaxation behavior at four strain levels (Liu and Bilston, 2002). A Maxwell-based model with 13 different variables has also been presented to evaluate the viscoelastic response of the liver under a variety of uniaxial preloads, frequencies, and strain rates (Ayyildiz et al., 2015). Large preloads (20%) and shear strains (5%) have been simultaneously utilized in the experimental protocol there. Nevertheless, the findings concentrate on the impacts of preload, strain rate, and frequency on the normal force and torque reaction (Tan et al., 2018). A comprehensive 3D model has been presented that can characterize the tissue reaction under different kinds of deformation and frequencies. Cross analysis of uniaxial preloads, such as 1–20%, shear strains of 1–50% and frequencies ranging from 0.5–2 Hz has been proposed (Tan et al., 2013), highlighting the rate-dependent nonlinear viscoelastic characteristic of the liver. The measurement protocol exhibits a strain softening phenomenon, which is counteracted by suggesting a new error standard that permits a certain level of flexibility in adjusting the linear inputs to the models. This type of analysis and the process of model fitting results in the determination of simplified constitutive models that contain the key ingredients required to accurately represent the aforementioned characteristics of the liver subjected to both combinatory deformation and a variety of frequencies. Specifically, this is apparently among the first liver investigations to examine combined large uniaxial and shear stresses at multiple frequencies, and suggests a 3D nonlinear viscoelastic model with the ability to account for the large-amplitude oscillatory response over a wide array of preloads and frequencies (Capilnasiu et al., 2020).

For modelling, it is hypothesized that bovine liver specimen are stress free and isotropic, when they are in the reference configuration. The monitored torque characteristics are fit to a viscoelastic adjustment of three hyperelastic models typically employed in soft-tissue mechanics to compare their adequacy for sculpting the dataset. These are the polynomial model (a modified type of the Mooney–Rivlin model, which is referred to as vMR*), which is the simplest model, the viscoelastic Ogden (vOG) model, which is based on the strain-energy function, and the viscoelastic exponential (vEXP) model, which is the isotropic exponential Fung-type model based on the strain energy function. The latter two models are similarly appropriate, as the two models are better at accounting for the nonlinear tendencies (Capilnasiu et al., 2020).

The mechanical features of lung parenchymal tissue represent elastic and dissipative elements, as well as they exhibit a highly nonlinear response. The mechanical response of lung tissue is based on a macroscopic phenomenon that is itself evoked by interactions of the microscopic elements. The mechanics of the pulmonary system are governed through the individual contributions of and the intricate interference among its major compounds. On the one hand, the lung tissues display a quasi-static mechanical behavior. On the other hand, there exist computational models that indicate how smooth nonlinear stress-strain response can emerge by a percolation-like event, where the sequential homing of collagen fibers with elevating strain renders them to progressively involved in performing the load-bearing function from elastin. The viscoelastic nature of lung tissue has been measured (de Hilster et al., 2019a). While the airway characteristics can be well presented through resistive and inertive properties (Hantos et al., 1992), the contribution of the lung tissues in the entire mechanical properties of the lung is more intricate, since there is a complex interference between the extravascular proteins, fibers, cells, surface film layer, and interstitial fluids (Bates et al., 2007; Faffe and Zin, 2009; Suki and Bates, 2011). The *in vitro* rebuilding of diseased and non-diseased states of the lung can be successfully performed employing hydrogels (de Hilster et al., 2019b).

Fibrosis and elevated stiffness are features of multiple solid cancers and encourage growth of the tumor. Cultivation of mammary epithelial cells on soft and rigid collagen matrices, respectively, reveals that with enhanced stiffness, epithelial tubulogenesis is reduced, contractility is augmented, and proliferation is amplified (Wozniak et al., 2003; Paszek et al., 2005; Provenzano et al., 2009). It has also been illustrated that growth in a more stiff ambient environment fosters an invasive phenotype (Provenzano et al., 2006). Many of these hallmarks are due to the enhanced activity of RhoA in cells when cultured in a rigid setting.

Attenuation of p190RhoGAP has been implicated as an additional mechanism for enhanced RhoA activity in settings of mechanical tension, such as in fibroblasts derived from patients with idiopathic pulmonary fibrosis (Monaghan-Benson et al., 2018). The etiology of idiopathic pulmonary fibrosis is unclear, but increased levels of TGFβ are an important contributor to fibrosis, and there is ample support for an influential role of RhoA in this disease and other kinds of fibrosis. Examination of the signaling mechanisms that increase RhoA activity revealed that p190RhoGAP activity has been suppressed in fibrotic fibroblasts and in reaction to TGFβ (Monaghan-Benson et al., 2018). Mechanism investigation identified that the expression of Rnd3/RhoE, an activator of p190RhoGAP, has been depressed through TGFβ. A hallmark of fibrotic tissue is its enhanced stiffness, which results from the accumulation of surplus extracellular matrix. Increased stiffness augments RhoA activity (Wozniak et al., 2003) and GEF-H1 has been associated with it (Heck et al., 2012). Remarkably, both Rnd3 expression and p190RhoGAP activity are reduced in fibroblasts attached to rigid supports, indicating that this signaling route also accounts for increased RhoA activity in cells subjected to a rigid environment (Monaghan-Benson et al., 2018). Since TGFβ activity is also induced upon stimulation by elevated mechanical tension and growth on stiff media (Wipff et al., 2007), this points to a beneficial feedforward pathway engaging Rnd3 and p190RhoGAP.

## 9 Final Remarks and Future Perspectives

The exploration of viscoelasticity is still a major issue and carries an enormous potential besides cell stiffness to serve as a reliable and possibly as a universal biomarker for cellular migration and invasion. It may also harbor the potential to be adaptable both the migration of individual cells and a collection of cells. In specific, the adaption of the viscoelastic characteristics of migratory cells through EMT-regulated adjustments of adherens junction seems to be critical for providing efficient collective migration through tissues that offer physically divergent structural and mechanical architectures. Moreover, it can be stated that viscoelasticity of the migratory microenvironment is crucial to induce cell migration, engineer a material that enables the efficient migration of cells, and/or to regulate migration through the process of durotaxis. Thereby, new advances strategies highlight possible mechanisms facilitating the transfer of mechanical cues into the cells, including the expression of traditional transcriptional controllers of EMT, and in turn their impact on the alteration of the viscoelastic phenotype of migrating cells and their local microenvironments. To understand these processes in a more precise manner the formation of hallmarks for the individual cell migration may be beneficial.

In addition, it has to be accounted for that molecular signal transduction causes remodeling events at various cellular length scales, such as plasma membrane, cytoskeleton, organelles and nuclear components, These cellular remodeling events alter the tissue including its viscoelastic characteristics, and this new viscoelasticity of the tissue environment can now act on a long-range timescale to impact cellular, molecular, and viscoelastic characteristics of a neighbor tissue, such as a mechano-molecular feedback circuit timing the various processes, including the development of tumor microenvironments. Since tissue interferences are seen at a chemical scale through the secretion of molecules, it would be promising to investigate the interaction of viscoelasticity and secreted molecules in the regulation of single and collective cell movements. The involvement of these types of mechano-molecular feedback interferences seem to be highly crucial in the advancement for the forming and engineering of organ-analogous structures, such as organoids, in the field of mechanics-based cancer or inflammation research. Thereby, it is even crucial to combine the analysis of molecular elements, viscoelastic variables of cells and their local microenvironment (Poh et al., 2014; Wrighton and Kiessling, 2015; Mierke, 2019).

Consequently, viscoelasticity represents a general characteristic feature for the vast majority of biological constituents and the majority of cells and tissues that experience a single force or multiple mechanical forces. Finally, the requirement of multidisciplinary studies combining biophysical and biochemical variables seem to be critical to obtain a knowledge of growing intricate living biological systems.

For future studies focusing on viscoelasticity and other cell mechanical characteristics, the biophysical probing techniques need to be comparable and hence an experimental study on the comparison of different biophysical techniques is highly needed to further explore the phenomenon of viscoelasticity and to develop a road-map on how to conduct viscoelasticity measurements and in what type of microenvironment. Ultimately, the future perspective is the investigation of viscoelasticity on various length scales ranging from single molecules to entire tissues or on different time scales in order to fully probe viscoelasticity.
